# Microbial and geochemical architecture of an active Scotian Slope cold seep

**DOI:** 10.3389/fmicb.2026.1709097

**Published:** 2026-02-23

**Authors:** Elish Redshaw, Gamra Oueslati, Unyime U. Umoh, Natasha MacAdam, Patricia Granados, Jeremy N. Bentley, Narges Ahangarian, Robbie Bennett, Venus Baghalabadi, Martin G. Fowler, Adam MacDonald, Casey R. J. Hubert, G. Todd Ventura

**Affiliations:** 1 Department of Earth Sciences, Saint Mary's University, Halifax, NS, Canada; 2 Nova Scotia Department of Energy, Halifax, NS, Canada; 3 Centre for Environmental Analysis and Remediation, Saint Mary’s University, Halifax, NS, Canada; 4 Natural Resources Canada, Dartmouth, NS, Canada; 5 Department of Pharmacology, Dalhousie University, Halifax, NS, Canada; 6 Applied Petroleum Technology (Canada) Ltd., Calgary, AB, Canada; 7 Geomicrobiology Group, University of Calgary, Calgary, AB, Canada

**Keywords:** ANME, archaea, biomarker, cold seep, methane, porewater, salt diapir, Scotian Slope

## Abstract

**Background:**

Deep marine cold seeps occurring along the seabed of continental margins are identified by their oasis-like ecosystems, which are largely fueled by the chemical energy of the venting fluids. Seep site 2A-1, situated at ~2,500 m water depth on the Scotian Slope of the North Atlantic was discovered in 2021. The seep hosts a large mussel encrusted, carbonate mound with biogenic methane bubbling up from a single vent. The emitted biogenic methane is primarily sourced from ~1 km below the seafloor within the basin bedrock that resides directly above the crest of an underlying salt diapir.

**Methods:**

A 600-m long transect composed of six push cores was collected across the seep structure. Downcore porewater ions and lipidomic profiles of 24 predominantly archaeal in origin lipid classes were tentatively identified and quantified across the transect.

**Results:**

The resolved lipidomes comprised of intact polar lipids, core lipids, core lipid degradation products, and photosynthetic pigments. These data were compiled as two-dimensional heatmaps to spatially examine vertical and lateral changes in the subsurface geochemical and microbiological architecture of the seep. Microbially mediated metabolic zones of elevated heterotrophy, denitrification, microbial sulfate reduction, and anaerobic methane oxidation were then mapped across the seep structure based on an integrated analysis of porewater geochemistry, bulk organic matter and its carbon isotope compositions, lipidomic diversity and biomarker proxy patterns.

**Discussion:**

Increased lipidomic diversity is shown to exist within the seep particularly at boundaries of high lateral geochemical gradients. Biomarker lipid proxies and porewater gradient changes indicate a microbial community dominated by ANME-1 and -2/−3 archaea that is mixed with, but also surrounded by, an envelope of microbial sulfate reduction.

**Discussion:**

Spatial changes in the stratified system highlight the complex interplay of micro- and macro-seepage and provide insights into the seep’s evolution and impact on microbial dynamics across the carbonate structure.

## 1 Introduction

Ocean floor cold seeps expel low temperature fluids that are often rich in reduced carbon (including gas to liquid range hydrocarbons) and sulfur gases. The highly reduced fluids sustain unique ecosystems that are distinct from the surrounding ocean floor (Levin, 2005; Suess, 2020; Cochran et al., 2022). Globally, these features release ~600,000 mt of oil and 20–50 Tg ·year^−1^ of methane gas into the marine environment, making them prominent sources of oil and gas to the oceans (Hovland et al., 1993; Kvenvolden and Cooper, 2003; Kvenvolden and Rogers, 2005). Cold seep fluid migration is driven by a variety of geophysical processes including plate and salt tectonism (Barnes et al., 2010; Cartwright, 2011; Talukder, 2012; Chowdhury et al., 2024) and sediment overburden and compression (Suess, 2020).

These systems host diverse microbial and macrofaunal communities that convert available volatile hydrocarbons into living biomass (Orphan et al., 2001; Teske et al., 2002; Knittel et al., 2005; Orcutt et al., 2010; Kleindienst et al., 2012; Niu et al., 2017). The process follows predictable depth dependent, energetically derived redox and diffusion limited gradients (Reeburgh, 2007) that are based on the availability of terminal electron acceptors and donors in the sedimentary environment. However, for cold seeps, excessive redox compression from the ebullition of reduced fluids can disrupt the formation of biogeochemical stratification (Borowski et al., 1996; Regnier et al., 2011). These systems also have accelerated rates of microbial activity that lead to compressed diffusion gradients (Sun, 2023; Zhong, 2025). In such cases, aerobic heterotrophy (Equation 1), closely followed by denitrification (Equation 2), may co-occur with ferric iron (Fe^3+^) and manganese oxides (Mn^4+^) mediated reductive pathways as well as alongside microbial sulfate reduction (MSR; Equation 3) leading to fully anoxic conditions in relatively shallow sediment depth.

$$C_{6}H_{12}O_{6}+6O_{2}\to 6CO_{2}+6H_{2}O$$ (1)

$$C_{6}H_{12}O_{6}+4NO_{3}^{-}+4H^{+}\to 6CO_{2}+N_{2}+6H_{2}O\;$$ (2)

$$2(CH_{2}O)+SO_{4}^{\;2-}\to 2\mathrm{HC}O_{3}^{-}+H_{2}S$$ (3)

The reduced fluids of cold seeps can be enriched in hydrocarbons such as methane (CH_4_). Much of this CH_4_ is removed by the aerobic and anaerobic oxidation of methane (AOM, Equation 4; Hinrichs et al., 1999; Boetius et al., 2000; Orphan et al., 2001), which produces the largest energy yield along the redox tower by consuming >90% of the methane within the shallow sulfate-containing sediments (Reeburgh, 2007; Knittel and Boetius, 2009; Boetius and Wenzhöfer, 2013).

$$CH_{4}+SO_{4}^{\;2-}\to \mathrm{HC}O_{3}^{\;-}+HS^{-}+H_{2}O$$ (4)

In these environments, sulfate mediated (S-AOM) activity is not only concentrated in the sulfate–methane transition zone (SMTZ), which marks the point where sulfate and methane concentrations overlap (Barnes and Goldberg, 1976) as mediated by a syntrophic consortium of anaerobic methane oxidizing archaea (ANME) and SRB (i.e., Boetius et al., 2000; Hinrichs et al., 1999; Kellermann et al., 2012). They can also extend into shallower sediment ranges that are fully dominated by MSR (i.e., Treude, 2014; Sultan et al., 2016; Riedinger, 2017). It is now known that AOM is driven by many additional electron acceptors (i.e., Haroon et al., 2013; He et al., 2018; Welte et al., 2016; Zhao et al., 2024) including, but not limited to NO_3_^−^, Fe^3+^, Mn^4+^, and humic acids. Within these realms AOM is a significant microbial process that generates large amounts of dissolved inorganic carbon (DIC; Jing et al., 2020).

Three anaerobic methane oxidizing archaea (ANME-1, −2 and −3) groups of the orders methanomicrobiales and methanosarcinales are involved (Boetius et al., 2000; Orphan et al., 2001; Blumenberg et al., 2004; Niemann et al., 2006; Niemann and Elvert, 2008) and appear to gain energy exclusively from AOM (Knittel and Boetius, 2009). ANME-1 and -2 occur in consortia with SRB from the *Desulfosarcina*/*Desulfococcus* (DSS) group (Boetius et al., 2000; Orphan et al., 2001). ANME-3 occurs with SRB related to *Desulfobulbus* spp. (Niemann et al., 2006). How AOM spatially exists within such zones of active methane seepage and redox compression is not completely resolved. But their presence alongside other metabolic strategies for organic matter oxidation (Equations 1–3) gives rise to highly energetic systems whereby the expelled DIC results in the formation of seep carbonates that further stabilizes the resulting deep seafloor bioherms. In this way, seep environments necessarily yield distinct lipid classes that can be directly associated with fluid seepage (Boetius et al., 2000; Orphan et al., 2001).

The role of seafloor cold seeps in modulating biogeochemical cycles and their complex microbially mediated processes remains an area of active research. One means of further constraining these systems is through the use of environmental lipidomics, which represents the whole-scale study of lipids within a defined ecological system. As the structural components of cellular membranes, lipids contribute a significant portion of preservable organic matter to Earth’s soils and sediments (i.e., Langworthy et al., 1983; Kohl and Rice, 1999). The abundance and diversity of lipidomes within the natural environment, coupled with their ability to persist over geological time scales enables their use for reconstructing present and past marine environments. Intact polar lipids (IPLs) can in part be treated as the products of living cells, which follows the expectation that upon senescence, the polar headgroups of these compounds will rapidly hydrolyze to form more stable core lipids (CLs; White et al., 1979; Harvey et al., 1986; Sturt et al., 2004). Limits to this include recalcitrant headgroups that persist within sedimentary environments over long intervals of time and CLs that comprise a portion of the membranes within living organisms. Nonetheless, IPLs, and to a lesser degree CLs, are chemotaxonomically unique and are specific to their environment (Yunker et al., 2005; Rossel et al., 2008; Pitcher et al., 2011), which lends to their use as biomarkers for the study of material cycling within marine environments (Rütters et al., 2002; Rossel et al., 2008; Schubotz et al., 2009; Elling et al., 2017). Distinct lipid biomarker signatures can also be assigned to each of the three ANME groups based on various lipid ratios and stable carbon isotope compositions (Blumenberg et al., 2004; Niemann and Elvert, 2008; Rossel et al., 2008). In this regard, environmental lipidomics can be used to reconstruct the microbial population (Schippers and Neretin, 2006; Lipp et al., 2008; Lipp and Hinrichs, 2009), community dynamics (Cao et al., 2015; Lv et al., 2022), and metabolite availability or preference (Kellermann et al., 2012; Wegener et al., 2016) for past and present environments. As such, lipidomic studies help elucidate the fundamental metabolic zones found within cold seeps with biomarkers being capable of resolving three ANME (−1, −2, −3) classes.

**Figure 1:**
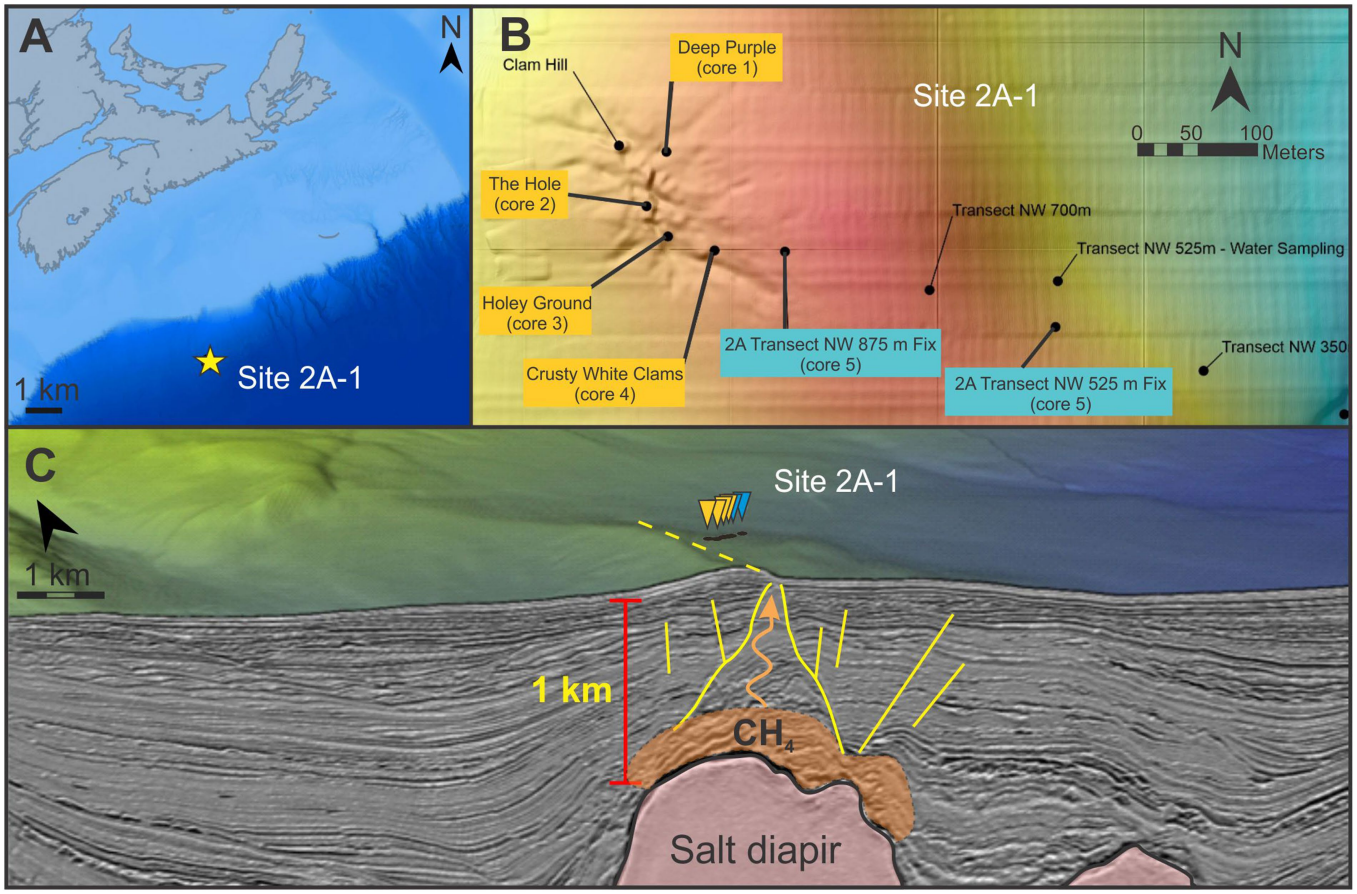
**(A)** Location of site 2A-1 on the Scotian Slope. **(B)** Push core transect sample locations used in this study (modified from Bennett and Desiage, 2022). Yellow and blue labels indicate seep and ambient sediments, respectively. **(C)** A 3D seismic bathymetric map and seismic crossline of the region. Yellow lines mark radial faults overlying the salt diapir. The orange region traces the generalized region for the seep’s primary methane source (with an orange arrow illustrating the gas migration pathway; Chowdhury et al., 2024). Images provided courtesy of Nova Scotia Department of Natural Resources and Renewables.

In this study, lipid biomarkers, alongside porewater and bulk geochemistry, are used to resolve the biogeochemical architecture of newly discovered cold seep 2A-1, which is located at ~2,500 mbsf along the Scotian Slope of Atlantic Canada (Figure 1). The geochemical reconstruction follows an ~600 m-long push-core transect that intersects the seep center and extends into ambient sediments thereby traversing a wide range of geochemical conditions. As such, these data were interpolated into 2D heat maps to better understand the spatial changes and microbial process operating in the shallow seep sedimentary environment.

## 2 Materials and methods

### 2.1 Sample collection

Cold seep 2A-1 is located 400 km off the south-eastern coast of Nova Scotia at a water depth of ~2,500 m (Figure 1; Campbell, 2019; Campbell and Normandeau, 2019; Bennett and Desiage, 2022). The seep sits directly above a fault splay, which is part of a larger network of crustal faults extending out from an underlying salt diapir whose crest sits ~1 km below seafloor (Figure 1). In 2021, the Atlantic Condor, outfitted with the Marine Environmental Observation, Prediction and Response Network’s (MEOPAR) Modular Ocean Research Infrastructure (MORI), undertook a remotely operated vehicle (ROV) coring survey to the Scotian Slope of Atlantic Canada. The ship was equipped with a Triton XLX ROV operated by Helix Robotic Solutions Ltd. (Houston, Texas). The massive carbonate mounds of the seep were largely encrusted with thick bivalve communities (Supplementary Figure S1). Gas seepage was observed within the mussel covered sediment of a carbonate depression, called The Hole (Bennett and Desiage, 2022). The microbial methane emitted from The Hole was determined to be sourced near the crest of the deeply buried diapir (Figure 1; Supplementary Figure S2; Chowdhury et al., 2024). A six-push core northwest-southeast trending transect was collected (Supplementary Figure S2; Table 1; Bennett and Desiage, 2022) that included four cores spanning a large portion of the seep structure and two additional cores collected within ambient sediments ~125 m and ~500 m southeast of the seep structure (Figure 1). Push cores penetrated a maximum depth of 40 cm below the seafloor (cmbsf). Core names were assigned based on the surficial characteristics of the coring location (Table 1). Upon collection, the push cores were immediately subsampled at 2-cm thick intervals down to 20 cmbsf and at 4-cm thick slices until the bottom of the core was reached. The sediment slabs were wrapped in pre-combusted aluminum foil and stored onboard the ship at −80 °C. All samples were kept frozen at that temperature until being further processed at the Organic Geochemistry Laboratory located at Saint Mary’s University (SMU).

**Table 1:** Push core sample summary.

Core number	Core name	Core depth (cmbsf)	Location (Lat. Long.)	Water depth (m)	Classification	Site characteristics
1	Deep Purple	28	42.163275, −62.372022	2,686	Seep	White and purple microbial mats
2	The Hole	20	42.162698, −62.372356	2,687	Seep	Authigenic Carbonates with gas hydrates and gas bubbles
3	Holey Ground	24	42.162353, −62.302762	2,688	Seep	Carbonate crater structure with bivalves
4	Crusty White Clams	38	42.162198, −62.371378	2,688	Seep	Mussel, bivalve and tube worm bed
5	NW 875 m	40	42.162180, −62.370351	2,686	Ambient	Normal marine sediments
6	Midpoint	36	42.161334, −62.366394	2,693	Ambient	Normal marine sediments

### 2.2 Bulk organic and inorganic carbon analyses

Total organic carbon (TOC) and particulate inorganic carbon (PIC) were measured across the seep transect. Approximately 2.5 g of frozen sediment was dried at 35 °C for a minimum of 12 h. The weight difference between the frozen and dried samples was recorded as a secondary measurement of sediment porosity. Once dry, sediment samples were treated with 5 mL of 6 N hydrochloric acid (HCl) and left to decarbonate for 24 h. Acid treated samples were neutralized by repetitive washing with de-ionized water until they reached a pH of 7. Neutral samples were again dried at 35 °C for 12 h before being stored at 4 °C. Sample PIC was estimated by the recorded weight differences between original dry sediment and dried neutralized sediments. The TOC measurements were collected using a Perkin-Elmer 2,400 Series II CHNS/O Elemental Analyzer (EA) programmed for CHN analysis at the Centre for Environmental Remediation (CEAR) at SMU. The EA was calibrated using Cyclohexanone 2,4-dinitrophenylhydrazone [Organic Analytical Standard (OAS), Elemental Analysis Lot No. BN330953] and cysteine (OAS, Perkin Elmer, Lot No. 090M1244V) was used as the conditioner. A matrix-matched, silty soil standard (Organic Analytic Standard, Elemental Microanalysis Ltd. Certificate No. 133507) was also measured to ensure an accurate reading of the sediment samples. For the analysis, 10 mg of neutral sediment was weighed into a foil tin and injected into the EA with Ar gas as the carrier.

### 2.3 Stable and radiogenic carbon isotope analyses

Thirteen decarbonated seep sediment samples from 4 cores were additionally selected for bulk organic stable carbon isotope measurements analysis. Samples were processed by the Isotope Science Laboratory at the University of Calgary, Alberta, Canada. Measurements were collected using continuous flow-elemental analysis-isotope ratio mass spectrometry coupled with an Elementar Isotope CUBE® elemental analyzer. Stable isotope ratios are expressed using delta notation (δ^13^C_TOC_) in part per mill (‰) difference between the sample and the ‘Vienna Peedee Belemnite’ formation for carbon (Craig, 1957). Four samples were also used for radiocarbon analysis to obtain the age and down-core sedimentation rates for ambient sediments outside the seep structure. Radiocarbon measurements were provided by the André E. Lalonde AMS Laboratory at the University of Ottawa. Samples were prepared following the methods outlined in Crann et al. (2017) and Murseli et al. (2019). Measurements were obtained using an Ionplus AG MICADAS (Mini Carbon Dating System) and reported in Δ^14^C yr. BP (BP = AD 1950).

### 2.4 Porewater analysis

Approximately 40 g of frozen sediment was placed into a cleaned polycarbonate centrifugation tube and left for up to 2 h to defrost. The sediment was then centrifugated for 10 min at 2500 rpm. The resulting supernatant porewater was pipetted out of the tube and passed through a 0.45-μm filter to remove residual suspended sediment particles. The volume of recovered porewater was recorded as the primary measure of sediment porosity. Alternating ~4 cm sediment intervals were assigned for anion and cation analyses. Anion measurements were obtained by ion chromatography. An additional mix of cation and anion concentrations were collected by photometry. The exception to this division was the 0–2 cm surface sediment interval, which was analyzed using both methods.

Ion chromatography was used to measure F^−^, NO_2_^−^, DIC (merged signal of HCO^−^ and CO_3_^2−^), SO_4_^2−^, and NO_3_^−^ concentrations. Filtered porewater was diluted in HPLC grade water [1:5] to a total injection volume of 600 μL into a Thermo Scientific Dionex Aquion Chromatography Conductivity System equipped with an anion-exchange column and a DS6 Heated Conductivity Cell fitted to an AERS 4-mm suppressor pump and a Dionex AXP auxiliary pump operating at a flow rate of 1.00 mL·min^−1^. The system was further equipped with two in-line guard cartridges: Thermo Scientific 9 × 24-mm Dionex InGuard Ag and Thermo Scientific Dionex InGuard Sodium (Na^+^) prep cartridges. The mobile phase was 25 mM NaOH in HPLC grade water. Sedimentary porewater ion concentrations were calculated based on an external calibration curve using an anion standard (Thermo Scientific Dionex Seven Anion Standard II)/carbonate stock (2000 mg·L^−1^ Na_2_CO_3_)/HPLC water [2:1:1, v/v/v] with standard mixture dilutions of 0.5, 1, 2, 5, 10, 20, and 50 ppm.

A Hanna Instruments HI-83300 Multiparameter Photometer was used to measure porewater ammonium (NH_4_^+^), Fe^2+^, Mn^2+^, and phosphate (PO_4_^3−^) concentrations. Prior to analysis, the photometer was calibrated through a series of absorbance measurements (420, 466, 525, 575, and 610 nm) to reach within an acceptable range of 0.02 abs. A 0.5–4 mL aliquot of filtered unreacted porewater was allocated to each ion. For NH_4_^+^, 1 mL of porewater was diluted in 9 mL of Hanna Instruments Ammonia Reagent A. Iron, Mn^2+^, and PO_4_^3−^ used varying amounts of porewater diluted in Milli-Q water to reach a total volume of 10 mL. Following the baseline read of the unreacted sample, the appropriate indicator solutions associated with NH_4_^+^, Fe^2+^, Mn^2+^, and PO_4_^3−^ were dissolved in the porewater sample. Concentration readings were re-calculated to account for individual dilution factors. The concentrations of Fe^2+^, Mn^2+^, and PO_4_^3−^ were only collected where porewater volume recoveries were large enough to allow for an accurate measurement.

### 2.5 Diffusion flux

Fluid discharge was largely gas seepage with a 1.48 Mg·y^−1^ discharge rate measured for The Hole (Chowdhury et al., 2024) and non-determinable, low seepage rates for the rest of the coring sites. Radiocarbon age data of the seep gas was used to trace the carbon source of the CH_4_ to an initial ocean DIC circulation cell with a flow rate of ≤1.25 m·y^−1^ driven by the salt diapir’s heat chimney effect (Chowdhury et al., 2024). The low fluid discharge suggests porewater ion concentrations are unlikely reflective of advective fluid flow such that sediment porewater diffusion fluxes can be calculated using Fick’s first law of diffusion under assumed steady state conditions (Equation 5). Diffusion flux (J_sed_) was therefore calculated from the sediments porosity (*φ*), diffusion coefficient (D_sed_), and the concentration gradient (∂C/∂x). Concentration gradients can indicate upward and downward material fluxes. For ions with multiple gradients, the upward- and downward-fluxes were averaged for the core. The diffusion coefficient for each ion was then calculated from sediment tortuosity and the ions diffusion coefficient in free solutions of seawater (D_sw_; Supplementary Table S1; Equation 6). Tortuosity (*θ*) is the mean ratio between the real length of the diffusion flux pathway and the straight-line distance and can be estimated from the sediment’s porosity (Equation 7).

$$J_{\mathrm{sed}}=-\phi \cdot D_{\mathrm{sed}}\cdot \frac{\partial C}{\partial x}$$ (5)

$$D_{\mathrm{sed}}=\frac{D^{\mathrm{sw}}}{\theta^{2}}$$ (6)

$$\theta^{2}=1-\ln(\phi^{2})$$ (7)

Diagenetic processes are time dependent. The sampling methods used in this study do not allow for this variable to be accurately calculated, sediment depth is instead employed as a means of modeling processes over time (Schulz, 2000). Results were further compared to approximal CH_4_ headspace analysis taken from adjected cores at sampling locations (Chowdhury et al., 2024).

### 2.6 Lipidomic analysis

Total lipid extracts (TLEs) were collected for 76 frozen sediment samples through modified Bligh and Dyer (MBD) extractions as outlined in Bentley et al. (2022) and Ahangarian et al. (2026). An aliquot representing 3% of the samples TLE was injected into an Agilent 1260 infinity ultra high-performance liquid chromatography-quadrupole time of flight mass spectrometer (UHPLC-qToF-MS) operated in reverse phase and with fitted with an electrospray ionization (ESI) source. The UHPLC was fitted with a ZORBAX RRHD Eclipse Plus C18 column (2.1-mm × 150-mm × 1.8-μm) with an Agilent Guard Column maintained at 45 °C using a flow rate of 0.300 mL·min^−1^. Two mobile phases and gradient elution were used for the separation. Mobile phase A (MeOH/formic acid (FA)/ammonium hydroxide (NH_4_OH) [100:0.04:0.10] v/v/v) ran 100% for 10 min and would then be progressively mix with mobile phase B (propan-2-ol (IPA)/FA/NH_4_OH [100:0.04:0.10] v/v). Mobile phases mixed on a linear gradient for an 85-min run. Lipids of interest were identified by their elution times, molecular ions [M^+^], and respective fragmentation patterns in Agilent Technologies MassHunter 10.0 Software. Integrated peak areas of the [M + H]^+^, [M + NH_4_]^+^, and [M + Na]^+^ adducts were summed in order to quantify targeted compounds (Wörmer et al., 2015; Bentley et al., 2022). Concentrations were calculated relative to the C46:0 internal standard and injection dilution. Quantitation is presented as μg·g^−1^ of sediment to normalize each lipid to the extracted sample sediment volume. Lipid concentrations were calculated relative to the C46:0 internal standard and reported in ug·g^−1^ sediment weight.

### 2.7 Statistical analysis

Principal component analysis was carried out using the “factomineR,” a package in RStudio (Version 4.3.2). The Kaiser-Meyer-Olkin (KMO) test of sampling adequacy and Barlett’s test of sphericity were employed to ensure the datasets suitability for PCA. A KMO score above 0.6 (and a *p*-value >0.05 for Barlett’s test) indicated that PCA was an appropriate approach for factor analysis. A scree plot was generated to determine the number of principal components to retain to capture the most significant variance. Results are visualized using the “factoextra” package.

Simpson’s Index (Simpson, 1949) measures community diversity by estimating the probability that two randomly selected individuals from a community belong to the same species with diversity (*D*) based on the proportion of individuals (*p*) of a given species (*i*). The proportion of individuals (*p_i_*) based on the number of individuals in the *i*^th^ species (n*_i_*) and the total number of species within the data set (*N*; Equations 8, 9). The results of the Simpson’s Index are often expressed as the complement (1-*D*) that ranges from 0 to 1 (with 1 representing infinite diversity).

$$D=\sum_{i=1}^{S}{\left(p_{i}\right)}^{2}$$ (8)

$$p_{i}=\frac{n_{i}}{N}$$ (9)

Here the index is applied to estimate lipidome diversity by setting *p* to be the proportion of lipids in the lipidome and n*_i_* the concentration of a lipid class with *N* as the total concentration of all lipid classes (e.g., Elling et al., 2017).

### 2.8 Lipid biomarker proxies

Lipid biomarker proxies were calculated for all samples. These include the methane index (MI; Equation 10), archaeol:hydroxy-archaeol (AR: OH-AR; Equation 11), and an experimental equation called the branched GDGT Sulfate Reduction Index (*br*SRI) for proxying sulfate reduction (Equation 12).


$$\mathrm{MI}=\frac{\left[\text{GDGT}-1]+[\text{GDGT}-2]+[\text{GDGT}-3\right]}{\begin{array}{l} {}[\text{GDGT}-1]+[\text{GDGT}-2]+[\text{GDGT}-3]+\\ \left[\text{GDGT}-5]+[\text{GDGT}-5^{\prime }\right] \end{array}}$$ (10)

$$\text{brSRI}=\frac{\left[\text{brGDGT}-\mathrm{Ia}\;]+[\text{brGDGT}-\mathrm{IIa}]+[\text{brGDGT}-\mathrm{IIb}\right]}{\begin{array}{l} {}[\sum \text{brGDGT}-\mathrm{Ia},b,c]+[\sum \text{brGDGT}-\mathrm{IIa},b,c]+\\ \left[\sum \text{brGDGT}-\text{IIIa},b,c\right] \end{array}}\mathrm{AR}:\mathrm{OH}-\mathrm{AR}=\frac{\mathrm{AR}}{\mathrm{OH}-\mathrm{AR}}$$ (11)

$$\text{brSRI}=\frac{\left[\text{brGDGT}-\mathrm{Ia}\;\right]+\left[\text{brGDGT}-\mathrm{IIa}\right]+\left[\text{brGDGT}-\mathrm{IIb}\right]}{\left[\sum \text{brGDGT}-\mathrm{Ia},b,c\right]+\left[\sum \text{brGDGT}-\mathrm{IIa},b,c\right]+\left[\sum \text{brGDGT}-\text{IIIa},b,c\right]}$$ (12)

## 3 Results

### 3.1 Sedimentation rate and bulk organic matter trends

**Figure 2:**
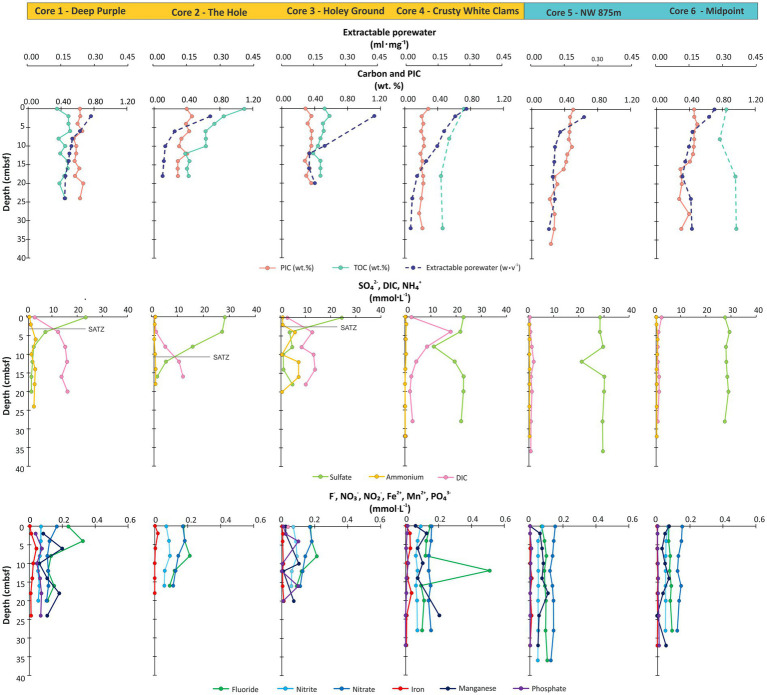
Sedimentary carbon and porewater ion survey. Dashed lines indicate discontinuous down core sampling. The SATZ is indicated by horizontal lines.

All push cores contained highly permeable, unconsolidated sand to silt-sized sediments with porosity deceasing by gravimetric compactions from 0.29 mL·mg^−1^ at 0–2 cmbsf to 0.10 mL·mg^−1^ at 36–38 cmbsf (Figure 2; Supplementary Table S2). Four down core TOC-based radiocarbon ages were taken from an ambient sediment core close to seep 2A-1. These measurements were made to reconstruct the age profile and sedimentation rate of the ambient seep area. Sediments reached a maximum age of 10,343 ± 31 Δ^14^C yr. BP within 40 cmbsf (Supplementary Figure S3). An average sedimentation rate of 0.38 mm‧yr.^−1^ (or 259 yr.‧cm^−1^) was then estimated after adjusting ^14^C values to account for the presence of dead carbon from the water column and by disregarding potential reservoir effects (Supplementary Table S2). Based on the extractable porewater results, the apparent decreasing sedimentation rate with depth is largely a function of sediment compaction.

Total organic carbon ranged from 1.10 to 0.35 wt.% (avg. 0.51 wt.%) with seep sediments from The Hole (core 2) and Crusty White Clams (core 4) containing greater decay rates with sediment depth (Figure 2). Bulk organic carbon isotope (δ^13^C_TOC_) values of the non-seep sediments (avg. -21.9 ± 0.4‰; Supplementary Table S3) are consistent with upper water column sourced organic matter input (δ^13^C_TOC_ = −21 ‰; Meyers, 1994; Ahangarian et al., 2026). Within the seep, The Hole and Holey Ground (marking cores 2 and 3), more depleted δ^13^C_TOC_ values (−40.1‰ and −41.9, respectively; Supplementary Table S3), indicate the shallow sedimentary carbon cycle is strongly influenced by incorporation of microbial sourced CH_4_ seepage (Whiticar, 1999; Joye, 2020; Chowdhury et al., 2024).

### 3.2 Porewater geochemistry

**Figure 3:**
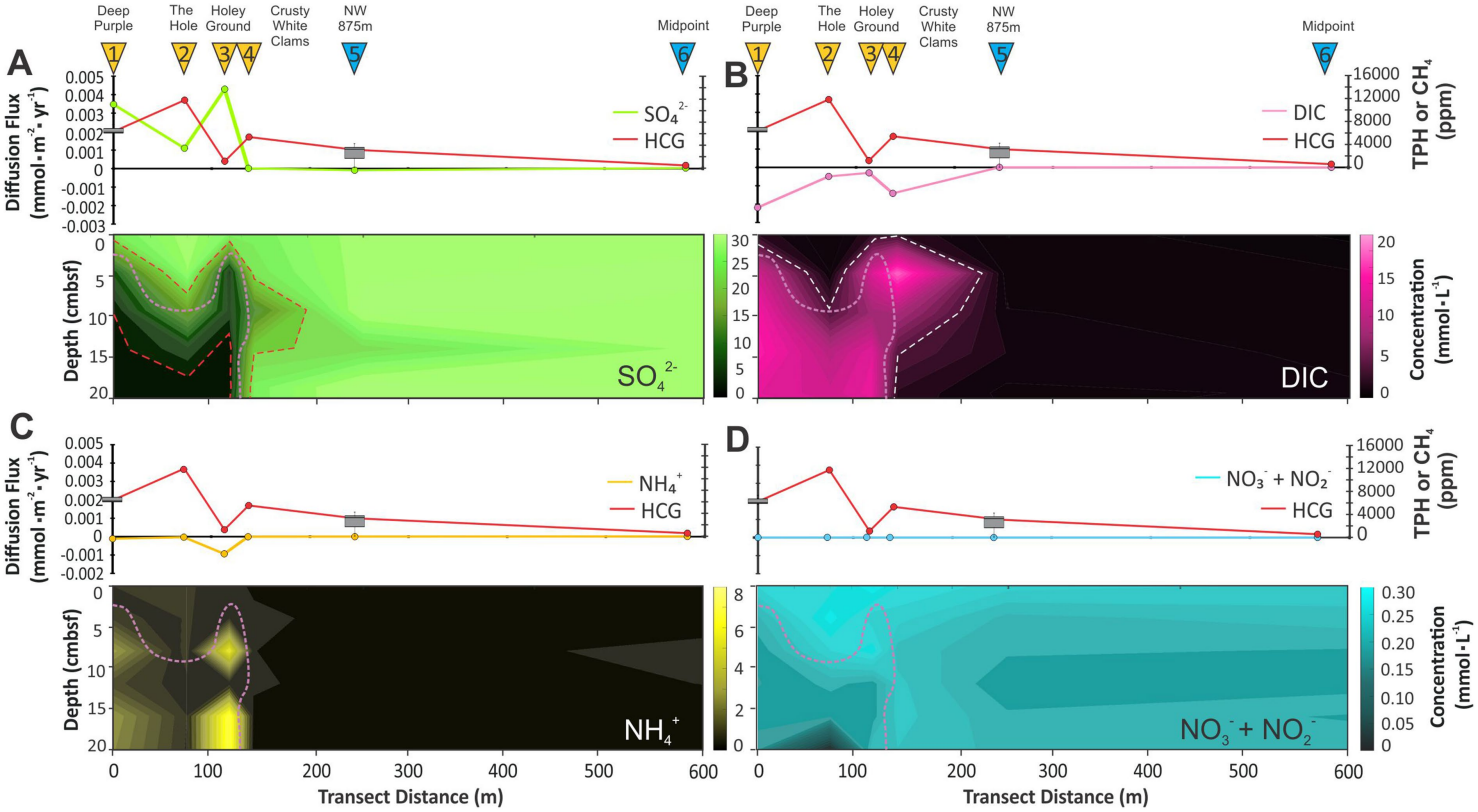
Cross-seep diffusion flux and core bottom hydrocarbon gases (HCG) concentrations measured as either total petroleum hydrocarbon (denoted as whisker plot) or as CH_4_ collected from the bottom of each push core (labeled by red filled circles; Supplementary Figure S4). These plots are nested over depth-distance interpolated porewater ion concentration heatmaps of **(A)** SO_4_^2−^, **(B)** DIC, **(C)** NH_4_^+^, and **(D)** NO_2_^−^ and NO_3_^−^. Dotted red lines indicate region of 95% SO_4_^2−^ loss. White dotted line indicates regions of high DIC concentration (>6 mmol·L^−1^). TPH is total petroleum hydrocarbons (see methods for further details). Pink lines mark the location of the SATZ.

Porewater ions of SO_4_^2−^, DIC, NO_3_^−^, NO_2_^−^, NH_4_^+^, Fe^2+^, Mn^2+^, PO_4_^3−^, and F^−^ were measured across the seep transect (Figure 2; Supplementary Table S3). Porewater ion diffusion fluxes were calculated at each core site for SO_4_^2−^, DIC, NO_2_^−^ and NO_3_^−^ (herein cojoined measures), and NH_4_^+^ (Figure 3). Diffusion fluxes enable tracking of ion movement from high to low zones of concentration with positive and negative values indicating the loss and gain of ion concentration with the sediment profile. These four ions were further interpolated across the transect as heatmaps to provide a spatial reconstruction of the soft sediment geochemical environment (Figure 3). As sediment depths vary for each core, for these and other similarly generated plots, heatmaps are uniformly cut to a maximum depth of 20 cmbsf conforming to the shallowest core in the transect survey. The maps show dramatic geochemical changes to the boundaries of the seep platform and surrounding ambient seafloor sediments.

Sulfate was the most abundant ion at near ocean water column concentrations (avg. 26.10 mmol·L^−1^; Figure 2; Supplementary Table S3) within near surface (0–2 cmbsf) and ambient down core sediments. Within the seep, SO_4_^2−^, concentrations rapidly taper to near zero in sediments as shallow as 10 cmbsf, which is further illustrated by the high positive diffusion fluxes at all seep cores (Figure 3). The reduction could be attributed to advection of reduced fluids or from elevated microbial activity. The deepest penetration of SO_4_^2−^ within the seep occurs at the site of highest gas discharge making advection unlikely.

**Figure 4:**
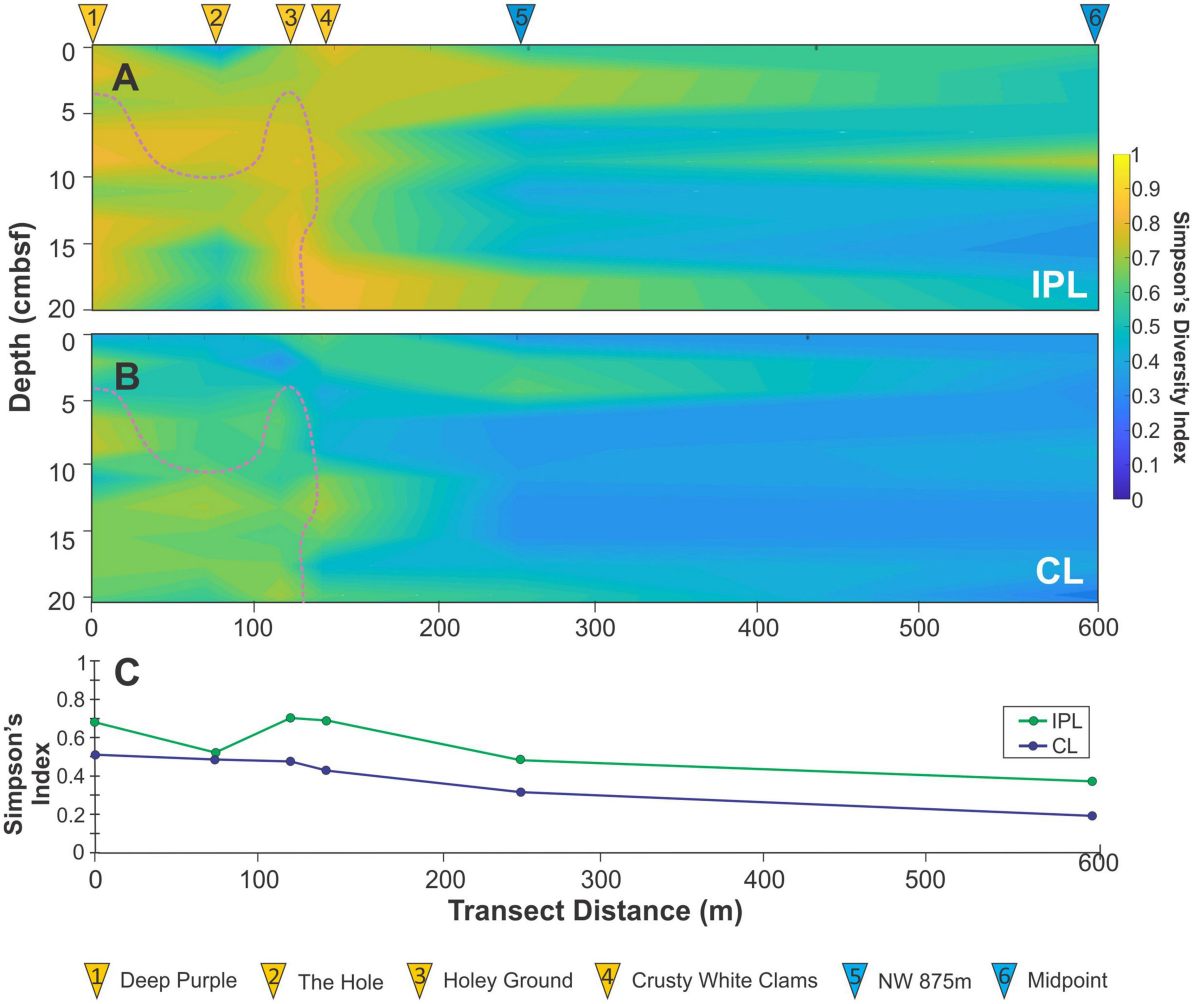
Simpson’s diversity index of **(A)** IPL and **(B)** CL transect heatmaps as well as **(C)** the average index value for the six-cores. Pink dotted line marks the location of the SATZ.

DIC marks the second most abundant porewater ion. Under typical seawater pH conditions, >99% of DIC is comprised of HCO^−^ and CO_3_^2−^ (Zeebe and Wolf-Gladrow, 2001), with the remainder being CO_2_. The residual portion of the DIC is therefore likely to have been sourced from gas seepage as bottom core seep headspace and void space CO_2_ concentrations represent 0.3 to ~4% of the hydrocarbon-based gas that is otherwise dominated by CH_4_ (Chowdhury et al., 2024). DIC concentrations exponentially increasing up to 18 mmol·L^−1^ within the deeper sections of cores 1–4, which is in stark contrast to the constant low concentrations (~0.58 mmol·L^−1^) observed across all sediment depth for the ambient regions of the transect (cores 5 and 6). The DIC produces inverse downcore abundance trends with SO_4_^2−^ across the transect. Changes in SO_4_^2−^ and DIC gradients were used to map the geochemical boundary of the seep. For marine sedimentary environments, these two ion gradients form a sulfate alkalinity transition zone (SATZ, i.e., Ahangarian et al., 2026), marking a point in the subsurface where both DIC and SO_4_^2−^ coexist in an ~1:1 ratio (Figure 4). The SATZ arises as a partial biproduct of AOM activity and therefore occurs at shallower burial depths than the SMTZ. As such, AOM must necessarily be limited to a region between the SATZ and where SO_4_^2−^ concentrations diminish to zero.

For nitrogen biogeochemical cycling, low NO_2_^−^ and NO_3_^−^ concentrations (0.30–0.05 mmol·L^−1^) with near zero diffusive fluxes indicate relatively weak nitrification and denitrification rates over the length of the transect (with a distinctly deeper penetration within The Hole; Figure 3). Isolated smaller negative NH_4_^+^ flux at Holey Ground (core 3) is due to isolated increases at 4–6 and 12–18 cm depth intervals. These spikes occurring within regions of high DIC and may be evidence of dissimilatory nitrate reduction.

Concentrations of F^−^ range from 0.71–0.07 mmol·L^−1^ (avg. 0.16 mmol·L^−1^) within the seep (cores 1–4) concentrations that decreased with depth suggesting calcium carbonate precipitation dominates the removal of dissolved fluoride from sea water, with incorporation into calcium phosphates (e.g., Carpenter, 1969). An inverse trend is observed for ambient cores 5 and 6 exhibited a steady increase with depth consistent with sequestration within the surrounding hemipelagic sourced muds. For the remaining measured ions, concentrations of Fe^2+^, Mn^2+^, and PO_4_^3−^ were low across all samples and exhibited no systematic down core trends, nor clear influences from gas seepage.

The porewater ion variations mark varying rates of seawater infiltration into the sediments that are buffered by gas discharge and enhanced biochemical cycling. These variations dramatically attenuate once CH_4_ concentrations rapidly fall moving outboard from the seep structure (Figure 3). Cross-plots of ion diffusion fluxes and hydrocarbon gases (HCG) based on the cojoined measurements of total petroleum hydrocarbons and CH_4_ (Chowdhury et al., 2024) were generated to further examine the influence of seepage on each ionic species (Figure 3; Supplementary Figures S4, S5). With the exception of the Holey Ground (core 3), increased HCG concentrations led to increased diffusion fluxes for SO_4_^2−^ and DIC. Both NH_4_^+^ and the combined NO_3_^−^ and NO_2_^−^ concentration appear to present a linear correlation with the HCG values. The two transect cores consistently plot with low HCG values and low diffusion fluxes (Supplementary Figure S5). We hypothesize that the lack of statistical alignment for the Holey Ground coring site is due to particularly high CH_4_ oxidation rates based isotopically depleted sedimentary organic matter (Supplementary Figure S3) and high ANME-1 based lipid loadings (see next sections).

### 3.3 Lipidomic survey

Lipid biomarkers included 56 archaeal sourced compounds, representing 24 compound classes, grouped as either IPLs, CLs, or CL decay products (CL-DPs) as well as four photosynthetic plant pigments, that were tentatively identified by their elution patterns and mass spectral characteristics as previously reported in the literature (Supplementary Figures S6, S7; Supplementary Tables S4A–C). These lipids were spatially resolved as 2D heatmaps, which display changing heterogeneous microbial community composition and organic matter loading across the seep.

#### 3.3.1 Lipid diversity and sourcing

For the detected lipidome, the diversity of both IPL and CL lipidomes were independently calculated across the transect (Supplementary Table S11) and reconstructed as Simpson’s diversity heatmaps (Figure 4). IPL diversity is necessarily higher than that of CLs due to headgroup variations being linked to the existing diversity of CLs. Nonetheless, higher lipidomic diversities were recorded for both groups within the seep structure relative to the ambient sediments with the highest levels occurring in Holey Ground (core 3) and Crusty White Clams (core 4) where geochemical gradients were most variable (Supplementary Figures S4, S6, S7; Supplementary Table S11). These trends happened to also occur near or at the SATZ. Lower relative IPL diversity was observed at The Hole (core 2).

#### 3.3.2 Archaeal intact polar lipids

The sourcing of lipid assemblages to various archaeal taxa is an ongoing and evolving effort. To date, within normal marine environments archaeal lipids commonly take the form of three fundament sources and activities: water column and near surface sediment ammonia oxidizers (e.g., AOA; i.e., Francis et al., 2005; Könneke et al., 2005; De La Torre et al., 2008; Hurley, 2016), anaerobic methane oxidizers (ANME; i.e., Niemann and Elvert, 2008; Rossel et al., 2008; Zhang et al., 2011; Kim and Zhang, 2023), and subsurface methanogens (i.e., Oba et al., 2015). For these different ecozones archaea biosynthesize a range of compounds that include C_40_ glycerol dialkyl glycerol tetraether (GDGT) having a multitude of ring configurations (0–8) or a C_20_ diphytanyl diethers (archaeol; AR). These compounds also can have a number of head group arrangements.

For seep 2A-1 the glycolipids, monoglycosyl (1G) and diglycosyl (2G) head groups linked to either GDGTs or ARs were the most common IPLs. These lipid sources appear to be largely geochemically zoned within three regions of the transect. The first regions is comprised of IPLs such as 1G-GDGTs, 2G-GDGTs, and 2G-OH-GDGTs (dominated by GDGT-0 and crenarchaeol; herein referred to as GDGT-5). These compounds mark the dominant lipids biosynthesized by ammonia-oxidizing *Thaumarchaeota* (Elling et al., 2017; Elling et al., 2015; Guan et al., 2024). For seep 2A-1 they are predominantly found in the shallow subsurface of The Hole and Holey Ground as well as within all depths for ambient sediments (cores 5 and 6). 2G-GDGT-5 was only detected in the ambient transect cores. These compounds have a maximum concentration of 0.37 μg‧g sed^−1^ (avg. 0.064 μg‧g sed^−1^).

**Figure 5:**
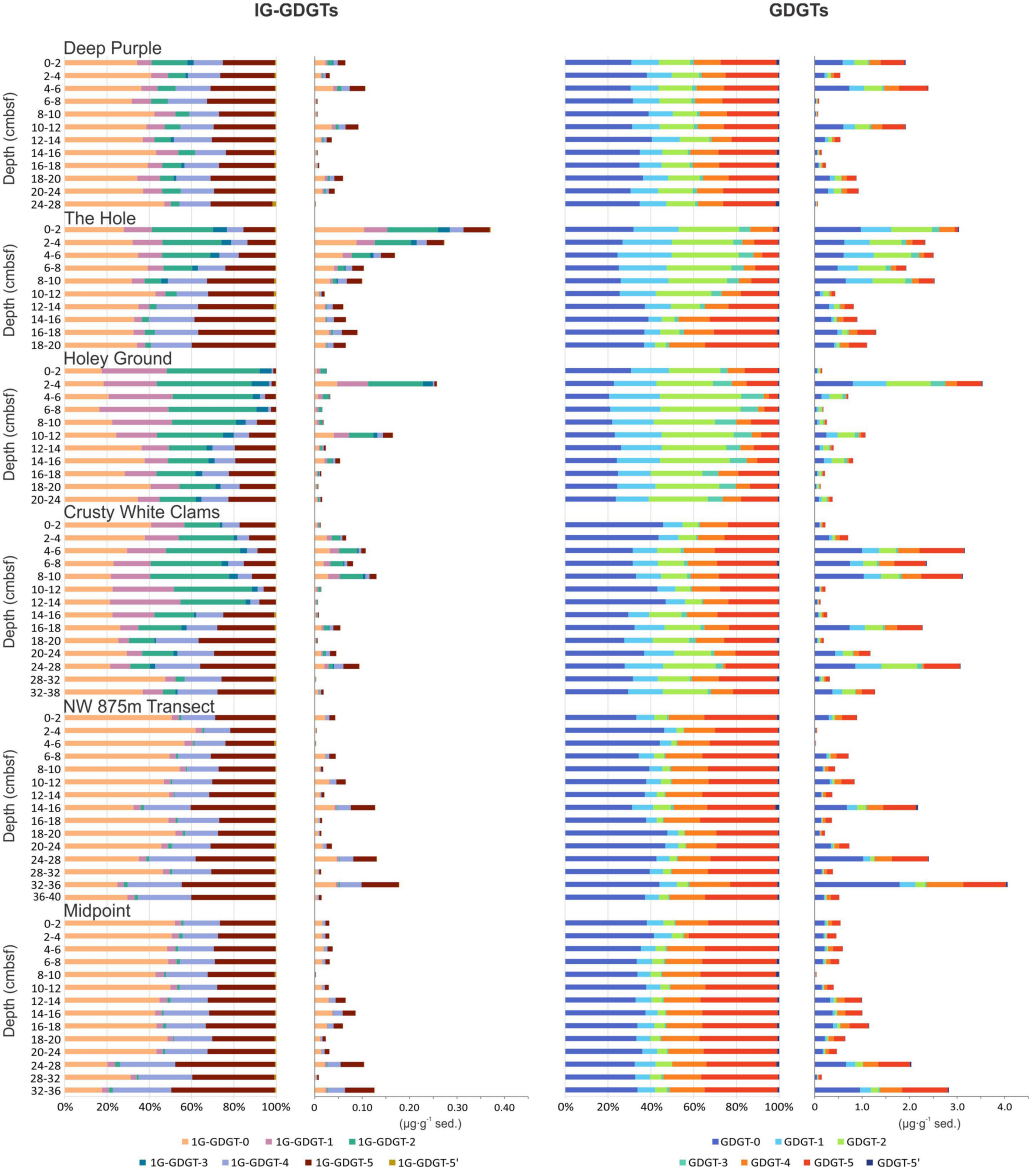
1G-GDGT and GDGT relative abundance and concentrations across site 2A-1 transect. Additional IPL-based compound ring associations are provided in Supplementary Tables S11–S17.

The second zone is dominated IPLs that have a high proportion of GDGT-1 to −3 that are associated with associated with ANME-1 communities (i.e., Blumenberg et al., 2004; Rossel et al., 2008; Zhang et al., 2011; Kurth et al., 2019; Kim and Zhang, 2023) attributed to Bathyarchaeia, previously known as the miscellaneous Crenarchaeotal group (MCG; Zhang, 2023). These lipids have a wider range of abundance extending to the basal sediments of all four seep cores (cores 1–4; Figure 5).

The third zone comprises of IPLs that are associated with ANME-2 and -3 dominated communities that synthesize higher abundances of AR and OH-AR and are involved in S-AOM (Blumenberg et al., 2004; Elvert et al., 2000; Rossel et al., 2008, 2011; Kurth et al., 2019; Zhang, 2023; Stock et al., 2025). IPL ARs, 1G- and 2G-AR were relatively abundant (avg. 0.03 and 0.02 μg‧g sed^−1^, respectively within the seep). In The Hole and Holey Ground (cores 2 and 3), their concentrations systematically decrease with depth. Crusty White Clams (core 4) had increase 1G- and 2G-AR concentrations to a depth of 8 cmbsf, followed by a systematic decrease to the core bottom. 1G- and 2G-AR concentrations substantially decrease (<0.01 μg‧g sed^−1^) in the ambient sediments of the transect with 2G-AR not being detected from the Midpoint transect (core 5). Crusty White Clams is loaded with ANME-2 and -3. PS-AR and PA-AR, lipid loading generally increases within the seep. IPL archaeols (PS-AR, PA-AR, and PG-AR) are largely absent in The Hole. Where present, PG-AR is enriched within Holey Ground. 2G-, 1G-AR, PS-AR, and PA-AR are enriched in Crusty White Clams. These compounds appear to be narrowly restricted to more reducing sediment conditions that closely align with the SATZ.

Archaeal lipids with phospho-based head groups were tentatively identified in low concentrations (<0.05 μg·g sed^−1^). PG-GDGT was only identified in The Hole (core 2) and in a few discrete intervals of Holey Ground and Crusty White Clams (cores 3 and 4). Similarly, PG-AR, and ANME-1 lipid (Kellermann et al., 2012), was also only identified in Holey Ground and Crusty White Clams. Two other headgroups, the phosphatidic acid (PA) and the phosphatidylserine (PS) were exclusively linked to AR and identified more consistently throughout the dataset. No phospholipid ARs were identified in The Hole (core 2), but these compounds were found throughout the other transect core samples with concentrations that decrease with sediment depth. The lack of phospholipids within site 2A-1 may be due to their faster degradation rate relative to glycolipids (Harvey et al., 1986; Schouten et al., 2010).

#### 3.3.3 Archaeal core lipids

**Figure 6:**
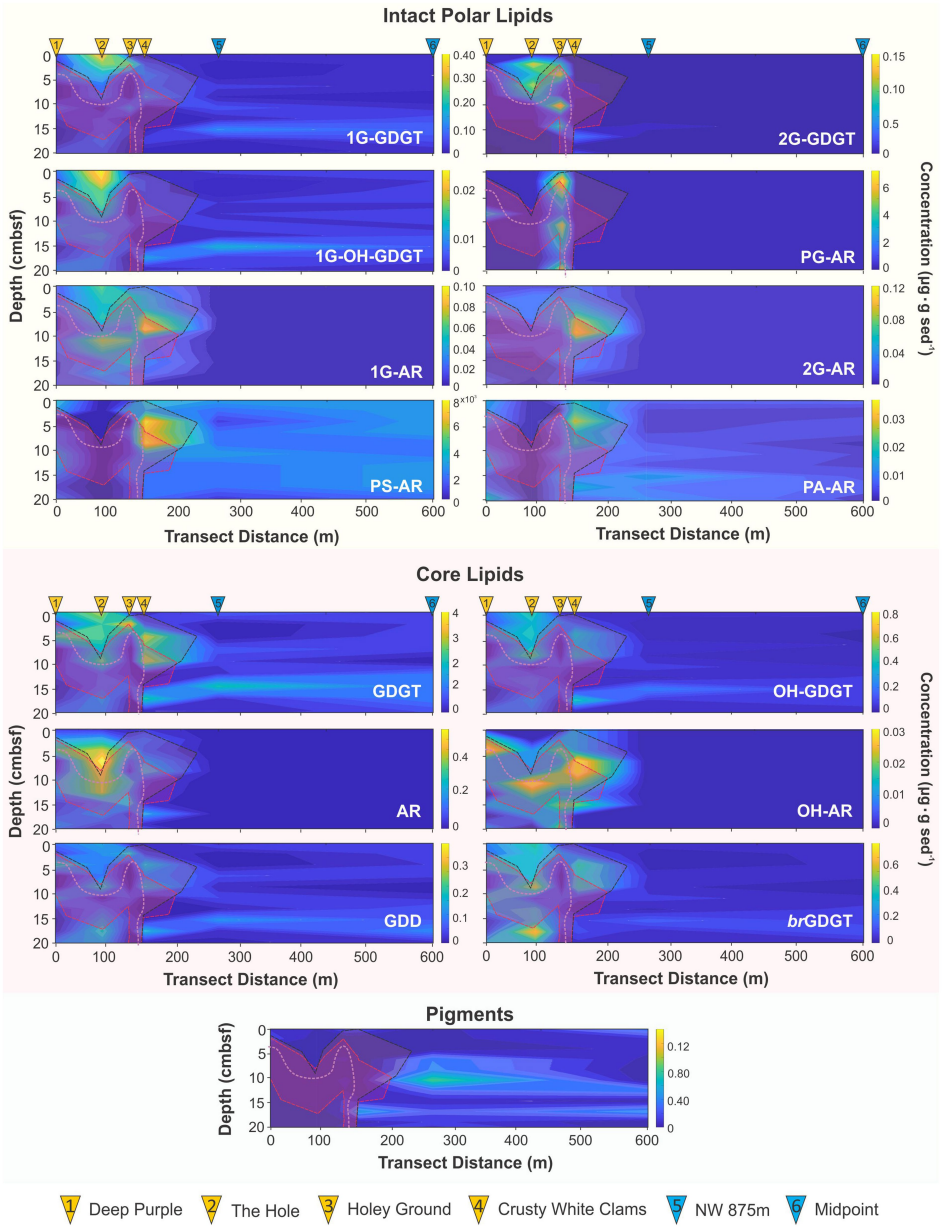
Transect heatmaps of archaeal IPLs, CLs, and pigments. Red, black, and pink dotted lines and shaded regions mark areas of high SO_4_^2−^ loss, DIC concentrations, and the SATZ, respectively as measured from Figure 3.

Isoprenoidal GDGT CLs were the most abundant archaeal lipids detected in the 2A-1 seep sediments. Similar to the 1G- and 2G-GDGTs (Figure 6), these compounds contain 0–3 cyclopentyl moieties, as well as crenarchaeaol (GDGT-5) and the stereoisomer of crenarchaeol (GDGT-5′; Supplementary Figure S7; Liu et al., 2018; Sinninghe Damsté et al., 2000). The summed concentration of GDGTs ranged from 4.07 μg‧g sed^−1^ to 0.02 μg‧g sed^−1^ for an average 1.04 μg‧g sed^−1^. Hydroxylated versions of these compounds occur in lower concentrations (avg. 0.15 μg‧g sed^−1^), and like their 1G- and 2G- precursors, only OH-GDGTs containing 0, 1, and 2 cyclopentyl moieties were detected. With the exception of Crusty White Clams (core 4), both GDGTs and OH-GDGTs systematically decreased downcore within the seep (Figure 6; Supplementary Figure S6). However, outside the seep (cores 5 and 6) concentrations of these compound classes increases with sediment depth. Archaeol (AR; avg. 0.10 μg‧g sed^−1^) is more abundant than its 1G- and 2G-AR precursors. The concentration of AR decreases with depth within the seep and increases with depth within the transect.

#### 3.3.4 Archaeal core lipid degradation products

Glycerol dibiphytanol diethers (GDDs) are composed of two biphytanyl chains bonded to a single glycerol moiety through two ether linkages (Thomas et al., 1979). Detected IPL-GDDs within an archaeal culture suggest these compounds can have a biosynthetic origin (Meador et al., 2014). However, GDDs are also treated as part of an extended class of GDGTs decay product based on the compound’s structure and distribution within natural sediments (Liu et al., 2012; Mitrović et al., 2023; Hingley et al., 2024; Ahangarian et al., 2026). The GDD-0 to −5 series is distinguished by an increasing number of cyclopentyl moieties (Supplementary Figure S7). Within the seep, GDD concentrations are variable and do not produce systematic downcore trends (Figure 6). However, within the ambient sediments (cores 5 and 6) GDD concentrations increase with depth to reach a maximum concentration of 0.34 μg‧g sed^−1^. Hydroxylated GDDs (OH-GDDs) containing 0, 1, and 2 cyclopentyl moieties were tentatively identified in trace amounts (avg. 0.01 μg‧g sed^−1^). OH-GDDs increase in concentration with depth within the ambient transect cores. Lastly, biphytanediols, a class of alcohol biomarkers that represent an extensive level of GDGT decay beyond that of GDDs (Schouten et al., 1998), were detected in all sediments. These single chain structures possess 0–3 cyclopentyl moieties. Detected biphytanediols have low concentrations (avg. ≤0.01 μg‧g sed^−1^) that increases with sediment depth.

#### 3.3.5 Bacterial core lipids

Branched-GDGTs (*br*GDGTs) are non-isoprene core lipids that are distinguished based on their degree of methylation (increasing from four to six for *br*GDGT-I through *br*GDGT-III) and cyclization (with the range from zero to two cyclopentane moieties; a,b,c; Sinninghe Damsté et al., 2000; Hopmans et al., 2004; Weijers et al., 2006; De Jonge et al., 2014; Xiao et al., 2016). In marine sediments these compounds are derived from the transportation of terrestrial materials as well as from the marine water column and sediments (Peterse et al., 2009; Liu et al., 2014; Zhang et al., 2020; Umoh et al., 2020; Xiao et al., 2022). While their biological sources have yet to be fully resolved, to date, *br*GDGTs have only been found within acidobacteria (Halamka et al., 2021; Chen et al., 2023), whose members are organotrophs that are known to engage in dissimilatory sulfate reduction (e.g., Dyksma and Pester, 2023; Demin et al., 2024; Valdez-Nuñez et al., 2024). *br*GDGT-Ia, -IIa (Zhang et al., 2020), and -IIb (Zhang et al., 2024) have been further shown to be significantly influenced by increased rates of AOM within cold seep systems. Additionally, 16S *r*RNA genes reveal a high correlation of *br*GDGT-Ia, -IIa, and -IIb with sulfate reducing bacterial groups, particularly those associated with AOM (Zhang et al., 2024). It is therefore plausible the source organisms of these compounds mark the bacterial syntrophic partner to the consortium. Within the seep transect *br*GDGTs (Figure 3B) were found to be preferentially loaded (avg. 0.14 μg‧g sed^−1^) within the seep sediments with hotspots being observed at the base of The Hole (core 2).

#### 3.3.6 Photosynthetic pigments

Photosynthetic pigments are ubiquitous components of marine sedimentary organic matter and may be used as an indicator of organic matter decay (Woulds and Cowie, 2009). Its presence in deep ocean sediments represents euphotic zone contributions to the benthic sediments. Photosynthetic pigments, chlorophyll *a* (Chl *a*) and pheophytin *a* (Pheo *a*), which is a breakdown product of Chl *a*, as well as their hydroxylated derivatives (OH-Chl *a* and OH-Pheo *a*), represent the primary allochthonous compounds in the seep area (Figure 6). Specific pigment decay products provide insights to grazing patterns (Ingalls et al., 2000; Woulds and Cowie, 2009) with the degradation pathway from Chl *a* to Pheo *a* involving the enzymatic removal of the central magnesium ion from the chlorophyll molecule in a process that can be accelerated by high thermal stress and acidification (Lajolo et al., 1971; Weemaes et al., 1999; Rydzyński et al., 2019), elevated sedimentary redox conditions, as well as from bioturbation (Bianchi et al., 2000; Ingalls et al., 2000). The systematic loss of both compounds (along with their hydroxylated counterparts), is however, heterotrophically regulated with pigment decay accelerated in zones of high redox gradients or where redox oscillations occur (Sun et al., 1995; Ingalls et al., 2000).

For the seep transect, all pigments recorded low concentrations (Chl *a* avg. 0.01 μg‧g sed^−1^ with OH-Chl *a* and OH-Pheo *a* avg. <0.01 μg‧g sed^−1^). Within the seep high redox gradient are associated with microbial mats and bivalves that have low pigment preservation (Figure 4; Supplementary Figure S6; Supplementary Table S5). In particular, the lowest pigment concentrations were found within The Hole (core 2), whose down core trend correlates with the recorded TOC decay profile (Figure 2) consistent with loss a loss by elevated heterotrophic activity. Pigment preservation notably increases outside the seep where higher organic matter preservation occurs also consistent with lower rates of heterotrophic activity for the surrounding ambient sediments.

### 3.4 Principal component analysis

**Figure 7:**
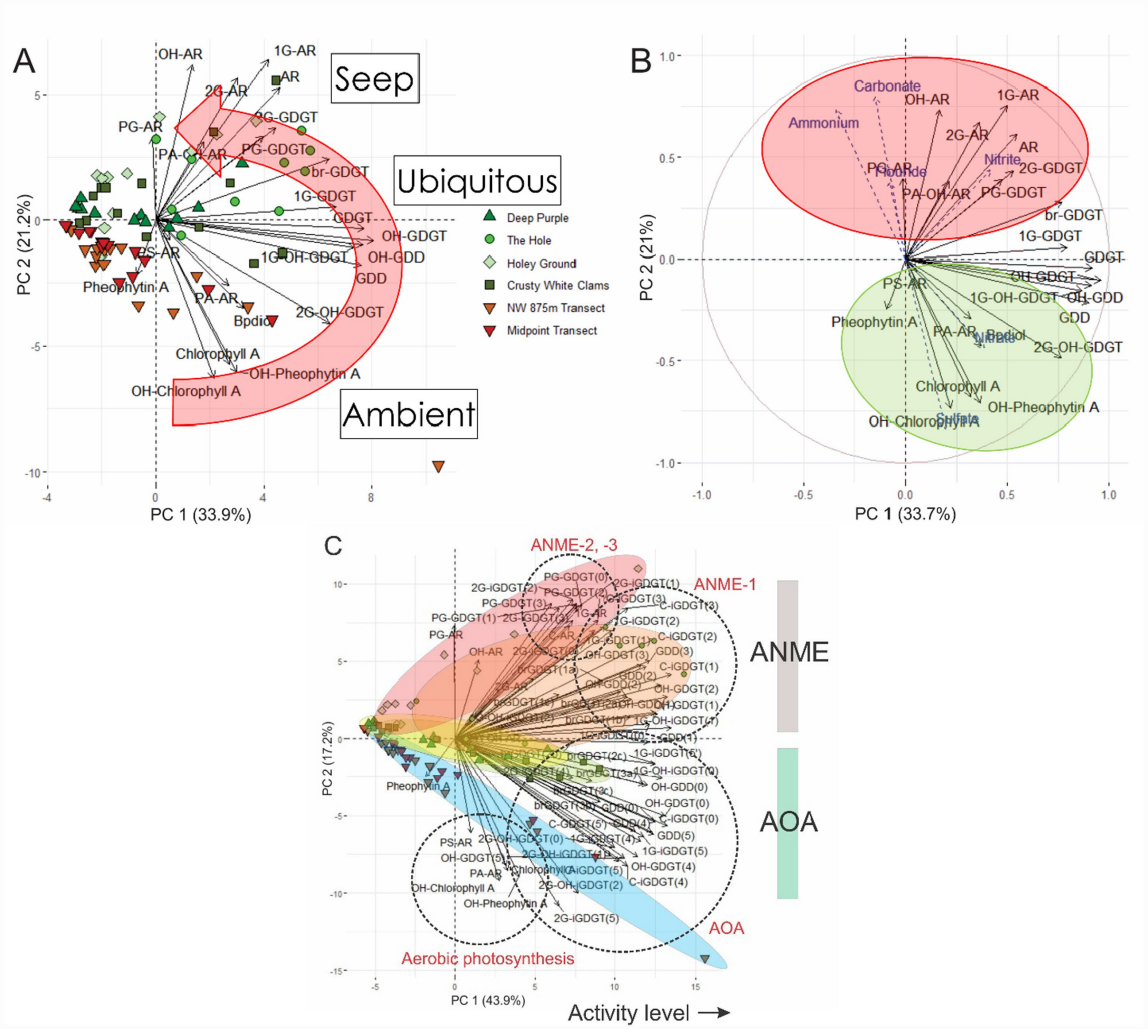
**(A)** PCA biplot of lipid classes and samples. **(B)** Combined PCA analysis of seep lipid classes and porewater ions. The porewater ions concentrations mark supplementary variables to the lipid concentrations. Red and green clusters group lipids within anoxic and dysoxic conditions, respectively. **(C)** For all quantified compounds distinct groupings in relation to metabolic affinities (colored ellipses enclose samples belong to a single push core) become resolved. Core samples (encapsulated in colored ellipses) from more active portions of the seep display higher PC2 loadings.

The distribution of membrane lipids and their relationship to seep porewater geochemistry was further examined by PCA (Figure 7; Supplementary Figures S8–S13). For lipids, the largest PC 1 loadings belonged to CLs: GDGTs, OH-GDGTs, GDDs, and OH-GDGTs as well as the IPLs: 1G-GDGTs and 1G-OH-GDGTs (Figure 7A). These compounds represent the most ubiquitous and, in most cases, abundant compounds across the transect. Alternatively, PC 2 divides ambient and seep settings as a function of redox environments based on sample distributions within the embedded scores plot. Lipid classes found predominantly in the seep have positive PC 2 factor loadings. The near zero PC 2 loadings for GDGTs is consistent with these compounds being detected in all surveyed sediments. Pigments have negative loadings that align with the ambient core samples.

These divisions are further confirmed with a combined porewater and lipidomic PCA (Figure 7B). Porewater ions such as DIC, NH_4_^+^, and NO_2_^−^ that have negative diffusion fluxes (Figure 3) produce high positive PC 2 factor loadings as found for enriched lipid classes within the seep environment. Similarly, porewater ions with high concentrations at depth, outside the seep, have negative PC 2 loadings. Alternatively, photosynthetic pigments in addition to SO_4_^2−^ and NO_3_^−^ produce similar eigenvectors. Ions with minimal change between seep and ambient sites have both moderate positive and negative loadings on PC 2, and plot close to more ubiquitous lipids species (such as GDGTs). When individual ring structures (−1, −2 and −3) are compared, IPL and CL GDGTs, OH-GDGTs, and GDDs similarly produce PC 2 positive factor loadings (Supplementary Figures 6, 7C) consistent with their affiliation with methane oxidizing archaea. Negative loadings for AOA based *Thaumarchaeota* IPLs, CLs, and DPs of GDGT-5 and -5′ and pigments. These results further indicate higher abundances of AMNE-1 and -2 sourced lipids reside within the seep sediments.

### 3.5 Interpretative indices and microbial community dynamics

#### 3.5.1 Microbial sulfate reduction

**Figure 8:**
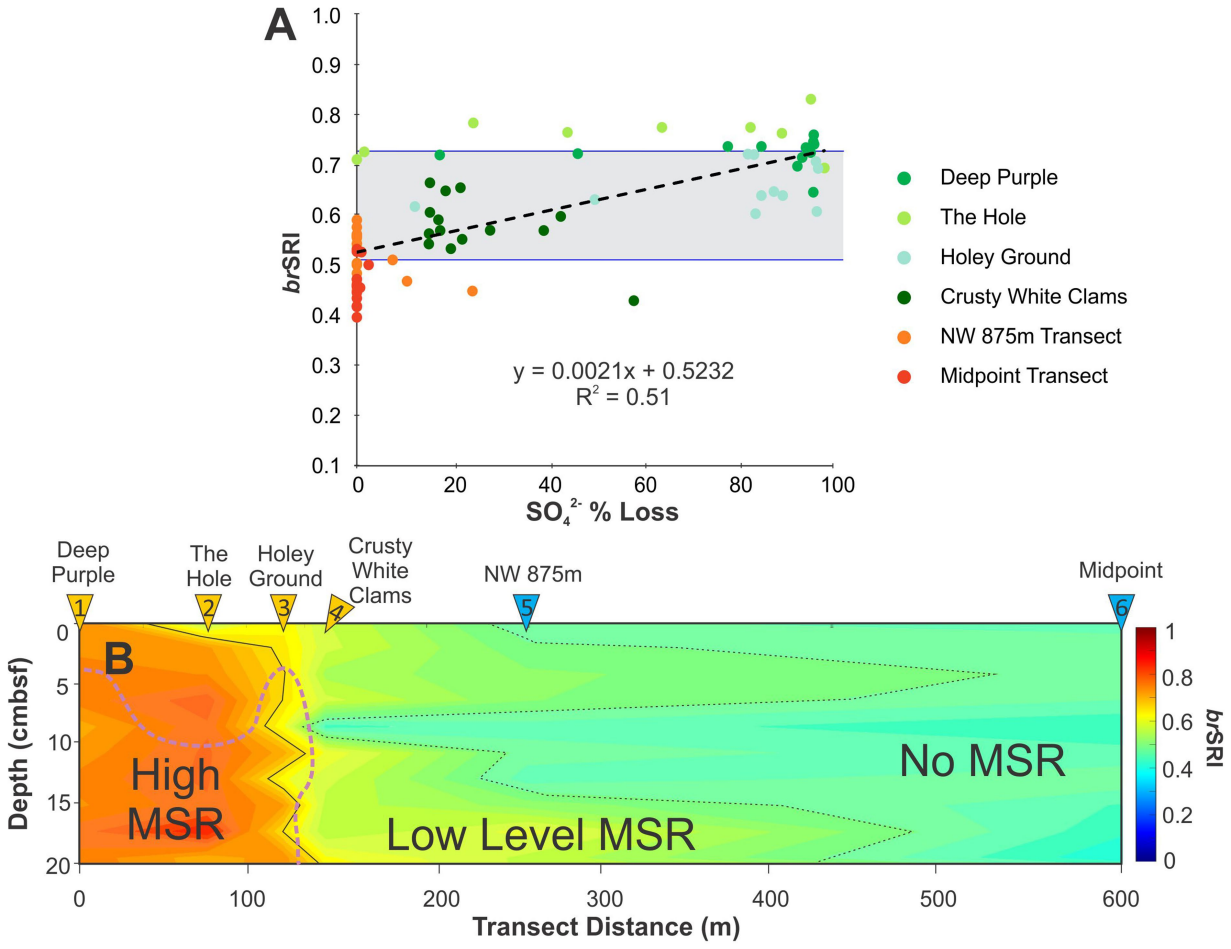
**(A)** Cross-plot of percent SO_4_^2−^ lost calculated as the difference between normal marine conditions (28 mmol·L^−*1*^) and the measured concentration of the sample vs. *br*SRI. Horizontal lines at ~0.5 and ~0.7 *br*SRI correspond to the mean minimum and maximum SO_4_^2−^ loss within the transect. **(B)** *br*SRI heatmap transect with dotted and solid lines marking transition zones between negligible, low, and high levels of MSR. Pink dotted line marks the location of the SATZ.

Rapid reductions in downcore porewater SO_4_^2−^ provides strong evidence for the presence of microbial sulfate reduction within the seep sediments. The large gradients observed at seep 2A-1 therefore allow for the testing of compatible biomarker proxies. To further evaluate the putative relationship, a similar logistic used by Zhang et al. (2011) is consider here as the *br*SRI (Equation 12) for identifying MSR zones within seep environments. To test the ratio, values were compared to the recorded percent loss of SO_4_^2−^ in sediment porewater relative to ambient seawater condition measured from the sample set (Figure 8A). A correlation (R^2^ = 0.51) to the PLS regression is consistent with *br*SRI values <0.5 mainly occurring within oxic to disoxic conditions and therefore marking conditions where MSR is not a dominant microbial process. The detection of MSR potentially arises at values >0.5 with an intermediate zone between 0.5 and 0.7 consistent with core porewater profile samples that display evidence of intensification of MSR. However, Deep Purple and The Hole (cores 1 and 2) also generated high *br*SRI values with shallow sediment samples having low sulfate depletion rates. These two cores instead produce near-zero slope trends that diverge from the overall transect regression line. To further evaluate these relationships a heatmap transect profile was produced to determine the spatial changes in *br*SRI values (Figure 8B). Based on these data, the ambient sediments sampled outside the seep are too shallow for significant levels of MSR to occur, which is consistent with the porewater SO_4_^2−^ profiles (Figures 2, 3). Within the seep transect, *br*SRI defined zones of MSR that closely match porewater geochemical spatial trends, but not to the level of tracking rapid shifts in porewater SO_4_^2−^ gradients. We suspect that this lack of congruency is due to different temporal constants on the two proxies. Whereas porewater ions represents the existing condition within the seep at the time of sampling, core lipid proxies mark a much longer time-averaged window of seep activity. Evidence of this effect is further documented below.

#### 3.5.2 Anaerobic methane oxidation

Highly depleted δ^13^C_TOC_ values within the seep sediments (Supplementary Table S3) necessarily require a portion of the preserved organic matter to be sourced from active carbon fixation using isotopically depleted microbial methane as a substrate for productivity in the shallow seep sediment biome. This requires a microbial methane oxidizing intermediate within the larger seep trophic structure. While the proportion of bacteria and archaea that are engaged in this cannot be determined from our data, the activity of anaerobic methanotrophic archaea (ANME) is resolvable using the methane index (MI; Equation 10). This GDGT-based molecular indicator, initially developed to detect the destabilization of marine gas hydrates (Zhang et al., 2011) or other methane impacted sediments (Kim and Zhang, 2023), compares the relative proportion of two end-member GDGT sources (methanotrophic ANMEs that belong to the phylum of *Euryarchaeota* with lipid signatures derive from planktonic *Thaumarchaeota*; Zhang et al., 2011; Kim and Zhang, 2023). Accordingly, in methane-rich environments, GDGT-1, −2, and −3, predominantly produced by ANME-1 (Blumenberg et al., 2004), become synthesized in larger quantities than planktonic *Thaumarchaeota* lipids (GDGT-0, −4, and crenarchaeol). As such, the MI qualitatively distinguishes ANME activity within methane impacted sediments (with values >0.3) from those that are normal marine. Later applications have employed IPL-based GDGT configurations in a modified MI_IPL_ proxy (e.g., Zhang, 2023; Kim and Zhang, 2023; Ahangarian et al., 2026) to further distinguish active versus more paleo ANME activity.

**Figure 9:**
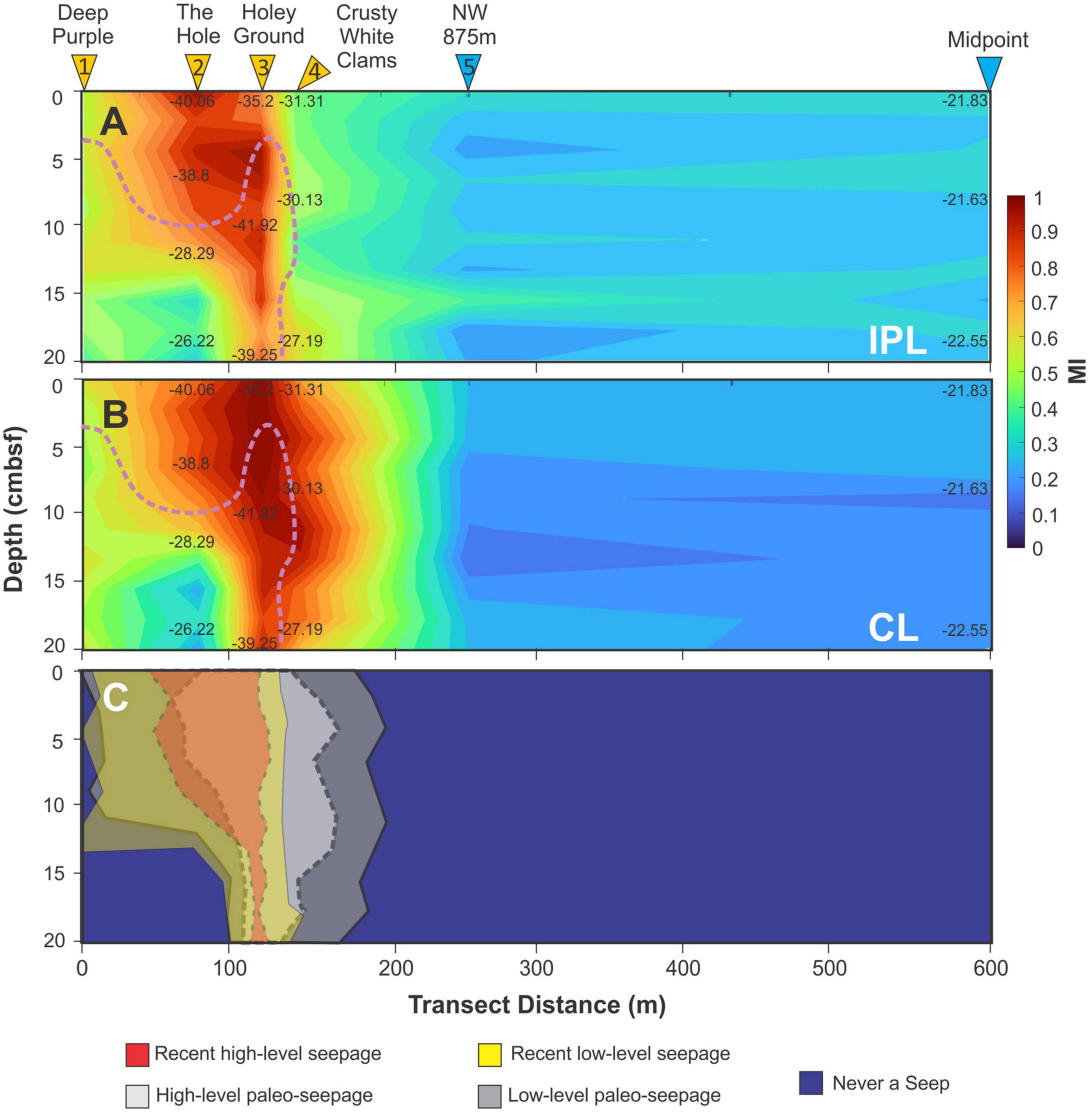
Heatmaps of **(A)** MI_IPL_ and **(B)** MI_CL_ marking the range of ANME-1 activity. Shaded regions mark areas of high SO_4_^2−^ loss and DIC loading as indicated in Figure 3. **(C)** Reconstruction of the inferred seepage history. Values on MI_CL_ and MI_IPL_ are δ^13^C_TOC_ measurements (Supplementary Tables S1, S12). Pink dotted line marks the location of the SATZ.

For Seep 2A-1, MI_IPL_ and MI_CL_ transect heatmaps were generated to further detail ANME responses to the changing seepage history (Figure 9; Supplementary Table S12). High MI values at Holey Ground (core 3) and The Hole (core 2) are coupled to high methane abundance within the transect cores (Figure 3) and depleted δ^13^C_TOC_ values (Figure 8; Supplementary Table S1). Interestingly, as The Hole (core 2) is the only documented site of macroseepage (Bennett and Desiage, 2022; Chowdhury et al., 2024), elevated MI values outside this location must necessarily describe zones of microbially active microseepage, which is consistent with high headspace gas recoveries from cores 1, 3, and 4 (Figure 3; Supplementary Figures S4, S5). Additionally, as the spatial changes in MI_IPL_ values closely match porewater gradient trends (Figure 3); the different MI_IPL_ and MI_CL_ profiles also indicate seepage activity had significantly decreased in more recent times.

An alternative indicator is the OH-AR: AR ratio (Equation 11; Niemann and Elvert, 2008), which is based on the finding that AR and OH-AR can have highly depleted carbon isotope compositions when in the presence of methane under anaerobic conditions (Hinrichs et al., 1999; Elvert et al., 2000). The ratio also capitalizes on the observation that ANME-2 type communities are not capable of internally synthesizing cyclized GDGTs but instead favor the production of OH-AR (Blumenberg et al., 2004). As such, OH-AR: AR values below ≤0.8 indicate an ANME-1 dominated community. For seep Site 2A-1 extremely low ratio values (avg. 0.11, SD = 0.11) were recorded further indicating the seep is dominated by an ANME-1 type community (Supplementary Table S12). However, isolated pockets of elevated OH-AR concentrations that are in the absence of IPL based OH-ARs suggests a historical presence of ANME-2 in Deep Purple, The Hole, and Holey Ground (cores 1–3; Figures 4, 5). These two markers are spatially zoned to high alkalinity regions within the seep.

ANME and SRB represent competing communities that directly interact within the SMTZ. However, unlike ANME-2 and -3 type communities, ANME-1 are only loosely associated with a bacterial partner occurring as single cells or linked together in short chains (Knittel and Boetius, 2009) leading to speculation that single-cell ANME-1 communities may couple AOM to sulfate reduction outside the confined range of the SMTZ (Orphan et al., 2002; Knittel et al., 2005). The dominance of an ANME-1 type community across regions of high SO_4_^2−^ gradients alongside moderate to high *br*SRI values supports the ability of ANME-1 to loosely couple AOM to a sulfate reducing partner within the seep.

## 4 Discussion

### 4.1 Microbial and geochemical seep architecture

**Figure 10:**
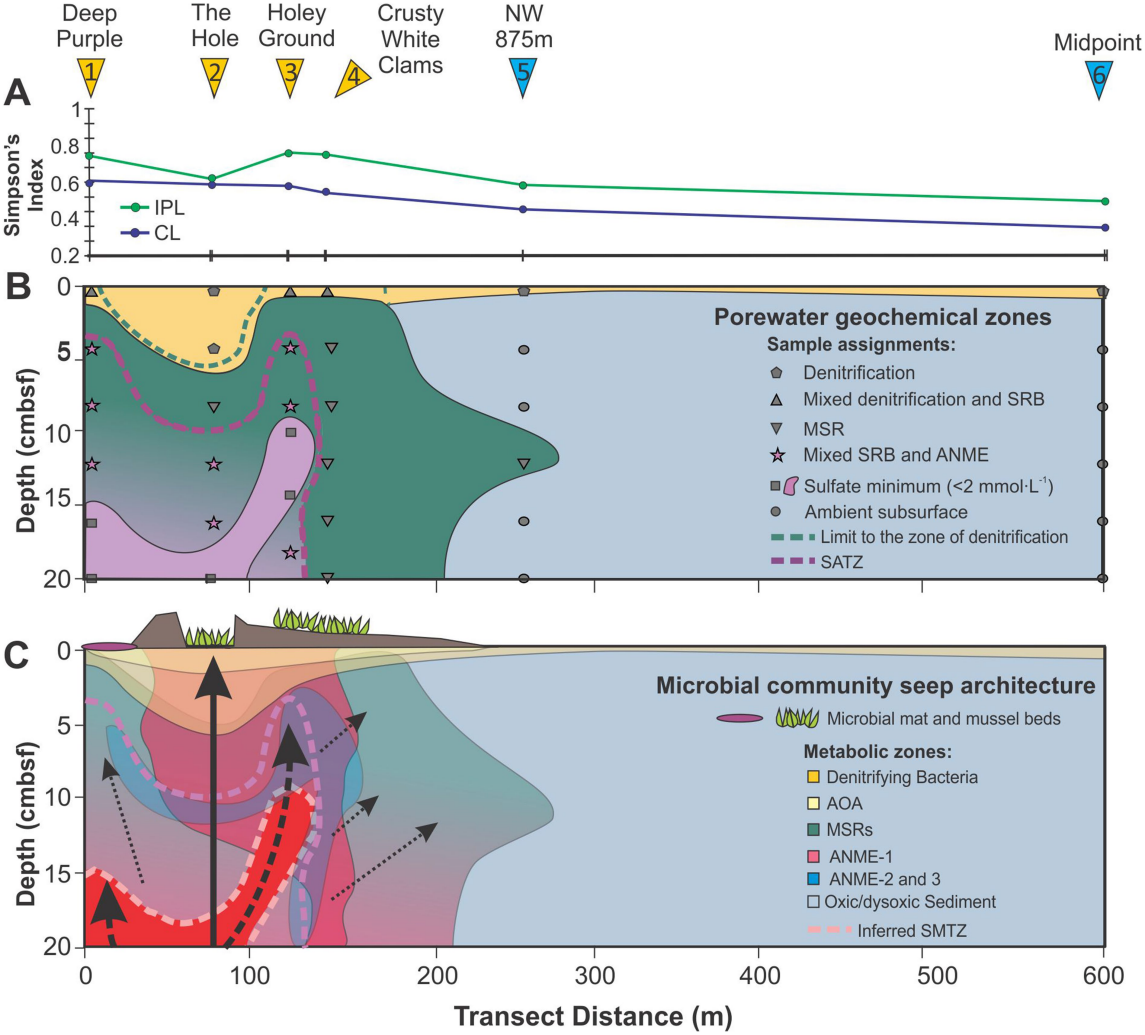
**(A)** Mean Simpson’s index changes across the 2A-1 seep transect. **(B)** The reconstructed seep architecture of porewater defined biogeochemical cycle further linked to **(C)** the lipid resolved microbial community structure. Only MI values of ≥0.7 are considered for the ANME habitable boundary. Dark arrow indicates major conduits of seepage. Black solid arrows indicate macro- and microseepage, respectively with thick line dotted lines indicating potential major paleo-outflow gas channels based on the differences between MI_CL_ to MI_IPL_ spatial ranges (Figure 9).

Biogeochemical cycling within cold seeps is primarily regulated by the geologic conditions that govern the rate and extent of reduced fluid transport (Orcutt et al., 2013). Evolving seepage rates will therefore impact the depth and lateral extent of peripheral redox zones (Borowski et al., 1996; Sommer et al., 2006), which further governs carbonate and gas hydrate precipitation. As the process continues mineralization decreases sediment porosity. These changes impact ion diffusion pathways (Solomon et al., 2008; Rooze et al., 2020) to the extent that carbonate production and gas hydrate formation preferentially occur along higher seep discharge conduits (Rooze et al., 2020). This can laterally shift seepage directions to different pipes when fluid flow is restricted. Resolving and further quantifying these processes can be difficult for active deep ocean seeps but underlies the reality that the geochemical conditions of these environments is dynamic and ever changing. Seep 2A-1 has likely been forming over the past 5,200 years based on measured sedimentation rate of the top 20 cmbsf of Supplementary Figure S3. During this time, methane seepage produced strong porewater redox gradients resulting in high porewater ion gradients (Figures 2, 3). To this extent, the geochemical seep architecture of the transect is highly zoned relative to a single macroseepage chimney flanked by two zones of high microseepage (Figure 10).

The portion of the transect marked by active macroseepage is surrounded by carbonate walls with gas hydrates precipitated within an overhanging ledge and a mussel bed colonizing the floor of the sediment filled crag. The incorporation of depleted δ^13^C into the sedimentary organic matter indicates reduced gas seepage and shallow carbon cycling at the sediment–water interface. Elevated NO_2_^−^ and NO_3_^−^ depletion highlight a zone of denitrification, which is itself constricted by a halo of near-surface MSR that occupies the larger seep structure (Figure 10). Within these sediments, the zone of denitrification dips to its deepest sediment depth across the transect. TOC depletion also produces its largest decay gradient (Supplementary Figure S3) signaling elevated rates of biodegradation. Collectively, these conditions likely arise from enhanced bio-irrigation by the colonizing macrofauna that are underpinned by microbial heterotrophy.

Additional, underlying this, and extending to the seep periphery, are increased concentrations of most archaeal lipid classes that signal the broad existence of three archaeal communities. Lipids associated with ammonia-oxidizing *Thaumarchaeota* (i.e., 1G-GDGTs, 1G-OH-GDGTs, GDGTs, OH-GDGTs dominated by GDGT-0 and -5) were preferentially located in shallower dysoxic to anoxic regions of The Hole where gas seepage was observed (Figures 4–5, 10). Higher loadings of these lipids were also found to extend into the shallow ambient environments indicating potentially elevated loadings of planktonic sourced OM. IPL and CL GDGT-1, −2, and −3, predominantly produced by ANME-1 (Blumenberg et al., 2004; Yanagawa et al., 2011) form a larger subsurface expanse that track seepage routes, but also extend to shallower dysoxic regions of the sediment profile (Figure 9). Lastly, IPL archaeols (i.e., PG-AR, 1G-AR, 2G-AR, PS-AR, PA-AR) linked to ANME-2 and -3, where preferentially concentrated at sites close to the SATZ and where SO_4_^2−^ depletion and DIC enrichment were maximized. Moving out from the seep, the reduced chemical energy resulted in archaeal lipidomic diversities decreasing alongside dramatically weakening rates of MSR that reflects significant community change. This occurs alongside gently widening of the zone of denitrification as deeper dysoxic conditions prevail withing the ambient sediments (Figure 10).

### 4.2 Comparison to other cold seep ecosystems

Pronounced downcore redox gradients have been observed in cold seep sediments from the Black Sea (Jørgensen et al., 2001; Niemann et al., 2006; Reitz et al., 2011), South China Sea (Cao and Lei, 2012; Luo et al., 2013; Chen et al., 2023), Gulf of Mexico (Joye et al., 2004; Joye et al., 2010; Formolo and Lyons, 2013; Bowles et al., 2016), and Mediterranean Sea (Werne et al., 2004; Haese et al., 2006; Pop Ristova et al., 2015). Rapid SO_4_^2−^ depletion within cold seep sediments has been largely attributed to upward fluxes of CH_4_ (Borowski et al., 1996). Similar down core redox gradients are observed in the 2A-1 cold seep ecosystems. Here site-specific biogeochemical zonation driven by methane flux, sediment chemistry, and electron acceptor availability, produce divergent AOM patterns and microbial architectures. For the Scotian Slope, 2A-1 identifies microbial zones of elevated heterotrophy, denitrification, and S-AOM, with lipid biomarkers dominated by ANME-1 co-occurring with active sulfate reducers, highlighting micro- and macro-seepage across carbonate structures. Ahangarian et al. (2026) also reported on Scotian Slope sediments, showing contribution from ANME-1, −2, and −3 with one lipidome marking seep-associated communities and overlapping lipidomes revealing systematic shifts in living, fossil, and diagenetically altered lipids along geochemical gradients. In south China, moderate methane flux favors different ANME communities, while low-flux sediments support boarder archaeal diversity (Zhang, 2023). In other cold seeps, methane-rich sediments compress the SMTZ and concentrate AOM, while organic matter oxidation can dominate SR and metal-driven AOM bellow SMTZ extending AOM beyond typical S-AOM pathways (Beal et al., 2009; Egger et al., 2015). These patterns reveal that methane flux, SMTZ depth, ANME composition, and electron acceptor availability shape microbial and lipidomic diversity across seep ecosystems.

## 5 Conclusion

This study provides a window into the microbial community structure, function, and history of seep 2A-1 in the Scotian Slope. Our findings highlight the dynamic interactions between gas seepage and microbial activity in shaping the geochemical environment. Geochemical gradient within the seep were largely driven by changes in SO_4_^2−^ and DIC, which are likely enhanced by AOM as regulated by varying degrees of macro- and microseepage. The resulting conditions promote higher microbial diversity that in return enhances rates of heterotrophy, mixed zones of denitrification, and the activity of ANME-1 communities. Biomarker ratios of intact and core lipids, further provide a glimpse into how the biogeochemical cycling and microbial community dynamics spatially changed over time suggesting the seep had been more active in the past.

## Data Availability

The original contributions presented in the study are included in the article/Supplementary material, further inquiries can be directed to the corresponding author.

## References

[ref1] Ahangarian N. Umoh U. MacAdam N. MacDonald A. Granados P. Bentley J. N. . (2026). Archaeal lipostratigraphy of the Scotian slope shallow sediments. Atlantic Canada, EGUsphere Biogeosci. 23, 21–38. doi: 10.5194/bg-23-21-2026

[ref2] Barnes R. O. Goldberg E. D. (1976). Methane production and consumption in anoxic marine sediments. Geology 4:297.

[ref3] Barnes P. M. Lamarche G. Bialas J. Henrys S. Pecher I. Netzeband G. L. . (2010). Tectonic and geological framework for gas hydrates and cold seeps on the Hikurangi subduction margin. New Zealand. Marine Geology 272, 26–48. doi: 10.1016/j.margeo.2009.03.012

[ref4] Beal E. J. House C. H. Orphan V. J. (2009). Manganese- and Iron-dependent marine methane oxidation. Science 325, 184–187. doi: 10.1126/science.1169984, 19589998

[ref5] Bennett R. Desiage P.-A. (2022). Expedition report 21CONDOR: Scotian slope, 2021, 8889.

[ref6] Bentley J. N. Ventura G. T. Dalzell C. J. Walters C. C. Peters C. A. Mennito A. S. . (2022). Archaeal lipid diversity, alteration, and preservation at the Cathedral Hill deep sea hydrothermal vent, Guaymas Basin, gulf of California, and its implications regarding the deep time preservation paradox. Org. Geochem. 163:104302. doi: 10.1016/j.orggeochem.2021.104302

[ref7] Bianchi T. S. Johansson B. Elmgren R. (2000). Breakdown of phytoplankton pigments in Baltic sediments: effects of anoxia and loss of deposit-feeding macrofauna. J. Exp. Mar. Biol. Ecol. 251, 161–183. 10960613 10.1016/s0022-0981(00)00212-4

[ref8] Blumenberg M. Seifert R. Reitner J. Pape T. Michaelis W. (2004). Membrane lipid patterns typify distinct anaerobic methanotrophic consortia. Proc. Natl. Acad. Sci. 101, 11111–11116. doi: 10.1073/pnas.0401188101, 15258285 PMC503748

[ref9] Boetius A. Ravenschlag K. Schubert C. J. Rickert D. Widdel F. Gieseke A. . (2000). A marine microbial consortium apparently mediating anaerobic oxidation of methane. Nature 407, 623–626. 11034209 10.1038/35036572

[ref10] Boetius A. Wenzhöfer F. (2013). Seafloor oxygen consumption fuelled by methane from cold seeps. Nat. Geosci. 6, 725–734. doi: 10.1038/ngeo1926

[ref11] Borowski W. S. Paull C. K. Ussler W. (1996). Marine pore-water sulfate profiles indicate in situ methane flux from underlying gas hydrate. Geology 24:655.

[ref12] Bowles M. Hunter K. S. Samarkin V. Joye S. (2016). Patterns and variability in geochemical signatures and microbial activity within and between diverse cold seep habitats along the lower continental slope, northern Gulf of Mexico. Deep-Sea Res. II Top. Stud. Oceanogr. 129, 31–40. doi: 10.1016/j.dsr2.2016.02.011

[ref13] Campbell D. C. (2019). CCGS Hudson expedition 2016–011, phase 2. Cold seep investigations on the Scotian slope, offshore Nova Scotia.

[ref14] Campbell D. C. Normandeau A. (2019). CCGS Hudson expedition 2018–041: High-resolution investigation of deep-water seabed seeps and landslides along the Scotian slope, offshore Nova Scotia.

[ref15] Cao C. Lei H. (2012). Geochemical characteristics of pore water in shallow sediments from north continental slope of South China Sea and their significance for natural gas hydrate occurrence. Procedia Environ. Sci. 12, 1017–1023. doi: 10.1016/j.proenv.2012.01.381

[ref16] Cao H. Zhang W. Wang Y. Qian P.-Y. (2015). Microbial community changes along the active seepage site of one cold seep in the Red Sea. Front. Microbiol. 6, 1–11. doi: 10.3389/fmicb.2015.00739, 26284035 PMC4523032

[ref17] Carpenter R. (1969). Factors controlling the marine geochemistry of fluorine. Geochim. Cosmochim. Acta 33, 1153–1167.

[ref18] Cartwright J. (2011). Diagenetically induced shear failure of fine-grained sediments and the development of polygonal fault systems. Mar. Pet. Geol. 28, 1593–1610. doi: 10.1016/j.marpetgeo.2011.06.004

[ref19] Chen C. Wu X. Wan Z. Shang J. Huang W. Zhang W. . (2023). Geochemical characteristics of sediment and pore water affected by cold seeps in southern South China Sea. Front. Mar. Sci. 10:1167578. doi: 10.3389/fmars.2023.1167578

[ref20] Chowdhury A. Ventura G. T. Owino Y. Lalk E. J. MacAdam N. Dooma J. M. . (2024). Cold seep formation from salt diapir–controlled deep biosphere oases. Proc. Natl. Acad. Sci. 121:e2316878121. doi: 10.1073/pnas.2316878121, 38466851 PMC10963010

[ref21] Cochran J. K. Landman N. H. Jakubowicz M. Brezina J. (2022). “Geochemistry of cold hydrocarbon seeps: an overview” in Ancient Hydrocarbon Seeps. eds. Kaim A. Cochran J. K. Landman N. H., vol. 53 (Springer International Publishing), 3–45.

[ref22] Craig H. (1957). Isotopic standards for carbon and oxygen and correction factors for mass-spectrometric analysis of carbon dioxide. Geochim. Cosmochim. Acta 12, 133–149.

[ref23] Crann C. A. Murseli S. St-Jean G. Zhao X. Clark I. D. Kieser W. E. (2017). First status report on radiocarbon sample preparation techniques at the a.E. Lalonde AMS Laboratory (Ottawa, Canada). Radiocarbon 59, 695–704. doi: 10.1017/RDC.2016.55

[ref24] De Jonge C. Hopmans E. C. Zell C. I. Kim J. -H. Schouten S. Sinninghe Damste J. S. (2014). Occurrence and abundance of 6-methyl branched glycerol dialkyl glycerol tetraethers in soils: implications for palaeoclimate. Geochim. Cosmochim. Acta 141, 97–112. doi: 10.1016/j.gca.2014.06.013

[ref25] De La Torre J. R. Walker C. B. Ingalls A. E. Könneke M. Stahl D. A. (2008). Cultivation of a thermophilic ammonia oxidizing archaeon synthesizing crenarchaeol. Environ. Microbiol. 10, 810–818. doi: 10.1111/j.1462-2920.2007.01506.x, 18205821

[ref26] Demin K. A. Prazdnova E. V. Minkina T. M. Gorovtsov A. V. (2024). Sulfate-reducing bacteria unearthed: ecological functions of the diverse prokaryotic group in terrestrial environments. Appl. Environ. Microbiol. 90:e0139023. doi: 10.1128/aem.01390-23, 38551370 PMC11022543

[ref27] Dyksma S. Pester M. (2023). Oxygen respiration and polysaccharide degradation by a sulfate-reducing acidobacterium. Nat. Commun. 14:1234567890. doi: 10.1038/s41467-023-42074-z, 37816749 PMC10564751

[ref28] Egger M. Rasigraf O. Sapart C. J. Jilbert T. Jetten M. S. Röckmann T. . (2015). Iron-mediated anaerobic oxidation of methane in brackish coastal sediments. Environ. Sci. Technol. 49, 277–283. doi: 10.1021/es503663z, 25412274

[ref29] Elling F. J. Könneke M. Mußmann M. Greve A. Hinrichs K.-U. (2015). Influence of temperature, pH, and salinity on membrane lipid composition and TEX86 of marine planktonic thaumarchaeal isolates. Geochim. Cosmochim. Acta 171, 238–255. doi: 10.1016/j.gca.2015.09.004

[ref30] Elling F. J. Könneke M. Nicol G. W. Stieglmeier M. Bayer B. Spieck E. . (2017). Chemotaxonomic characterisation of the thaumarchaeal lipidome. Environ. Microbiol. 19, 2681–2700. doi: 10.1111/1462-2920.13759, 28419726

[ref31] Elvert M. Suess E. Greinert J. Whiticar M. J. (2000). Archaea mediating anaerobic methane oxidation in deep-sea sediments at cold seeps of the eastern Aleutian subduction zone. Org. Geochem. 31, 1175–1187. doi: 10.1016/S0146-6380(00)00111-X

[ref32] Formolo M. J. Lyons T. W. (2013). Sulfur biogeochemistry of cold seeps in the green canyon region of the Gulf of Mexico. Geochim. Cosmochim. Acta 119, 264–285. doi: 10.1016/j.gca.2013.05.017

[ref33] Francis C. A. Roberts K. J. Beman J. M. Santoro A. E. Oakley B. B. (2005). Ubiquity and diversity of ammonia-oxidizing archaea in water columns and sediments of the ocean. Proc. Natl. Acad. Sci. 102, 14683–14688. doi: 10.1073/pnas.0506625102, 16186488 PMC1253578

[ref34] Guan H. Liu L. Birgel D. Peckmann J. Feng D. Li S. (2024). Hydroxylated GDGTs-0 in marine methane seep environments: a putative indicator for archaeal methanogenesis. Org. Geochem. 198:104862. doi: 10.1016/j.orggeochem.2024.104862

[ref35] Haese R. R. Hensen C. De Lange G. J. (2006). Pore water geochemistry of eastern Mediterranean mud volcanoes: implications for fluid transport and fluid origin. Mar. Geol. 225, 191–208. doi: 10.1016/j.margeo.2005.09.001

[ref36] Halamka T. A. McFarlin J. M. Younkin A. D. Depoy J. Dildar N. Kopf S. H. (2021). Oxygen limitation can trigger the production of branched GDGTs in culture. Geochemical Perspectives Letters 19, 36–39. doi: 10.7185/geochemlet.213

[ref37] Haroon M. F. Hu S. Shi Y. Imelfort M. Keller J. Hugenholtz P. . (2013). Anaerobic oxidation of methane coupled to nitrate reduction in a novel archaeal lineage. Nature 500, 567–570. doi: 10.1038/nature12375, 23892779

[ref38] Harvey H. R. Fallon R. D. Patton J. S. (1986). The effect of organic matter and oxygen on the degradation of bacterial membrane lipids in marine sediments. Geochim. Cosmochim. Acta 50, 795–804.

[ref39] He Z. Qingying Z. Yudong F. Hongwei L. Xiangliang P. Geoffrey M. G. (2018). Microbiological and environmental significance of metal-dependent anaerobic oxidation of methane. Sci. Total Environ. 610-611, 759–768. doi: 10.1016/j.scitotenv.2017.08.140, 28830047

[ref40] Hingley J. S. Martins C. C. Walker-Trivett C. Adams J. K. Naeher S. Häggi C. . (2024). The global distribution of Isoprenoidal glycerol Dialkyl Diethers (isoGDDs) is consistent with a predominant degradation origin. Org. Geochem. 192:104782. doi: 10.1016/j.orggeochem.2024.104782

[ref41] Hinrichs K.-U. Hayes J. M. Sylva S. P. Brewer P. G. DeLong E. F. (1999). Methane-consuming archaebacteria in marine sediments. Nature 398, 802–805. 10235261 10.1038/19751

[ref42] Hopmans E. C. Weijers J. W. H. Schefuß E. Herfort L. Sinninghe Damsté J. S. Schouten S. (2004). A novel proxy for terrestrial organic matter in sediments based on branched and isoprenoid tetraether lipids. Earth Planet. Sci. Lett. 224, 107–116. doi: 10.1016/j.epsl.2004.05.012

[ref43] Hovland M. Judd A. G. Burke R. A. (1993). The global flux of methane from shallow submarine sediments. Chemosphere 26, 559–578.

[ref44] Hurley S. J. (2016). Nfluence of ammonia oxidation rate on thaumarchaeal lipid composition and the TEX86 temperature proxy. Proc. Natl. Acad. Sci. 113, 7762–7767. doi: 10.1073/pnas.1518534113 27357675 PMC4948339

[ref45] Ingalls A. E. Aller R. C. Lee C. Sun M.-Y. (2000). The influence of deposit-feeding on chlorophyll-a degradation in coastal marine sediments. J. Mar. Res. 58:4.

[ref001] Jing H. Wang R. Jiang Q. Zhang Y. Peng X. (2000). Anaerobic methaneoxidation coupled to denitrification is an important potential methane sink in deep-sea cold seeps. Sci. Total Environ. 748:142459. doi: 10.1016/j.scitotenv.2020.142459 33113688

[ref46] Jørgensen B. B. Weber A. Zopfi J. (2001). Sulfate reduction and anaerobic methane oxidation in Black Sea sediments. Deep-Sea Res. I Oceanogr. Res. Pap. 48, 2097–2120. doi: 10.1016/S0967-0637(01)00007-3

[ref47] Joye S. B. (2020). The geology and biogeochemistry of hydrocarbon seeps. Annu. Rev. Earth Planet. Sci. 48, 205–231. doi: 10.1146/annurev-earth-063016-020052

[ref48] Joye S. B. Boetius A. Orcutt B. N. Montoya J. P. Schulz H. N. Erickson M. J. . (2004). The anaerobic oxidation of methane and sulfate reduction in sediments from Gulf of Mexico cold seeps. Chem. Geol. 205, 219–238. doi: 10.1016/j.chemgeo.2003.12.019

[ref49] Joye S. B. Bowles M. W. Samarkin V. A. Hunter K. S. Niemann H. (2010). Biogeochemical signatures and microbial activity of different cold seep habitats along the Gulf of Mexico lower slope. Deep-Sea Res. II 57, 1990–2001. doi: 10.1016/j.dsr2.2010.06.001

[ref50] Kellermann M. Y. Wegener G. Elvert M. Yoshinaga M. Y. Lin Y.-S. Holler T. . (2012). Autotrophy as a predominant mode of carbon fixation in anaerobic methane-oxidizing microbial communities. Proc. Natl. Acad. Sci. 109, 19321–19326. doi: 10.1073/pnas.1208795109 23129626 PMC3511159

[ref51] Kim B. Zhang Y. G. (2023). Methane index: towards a quantitative archaeal lipid biomarker proxy for reconstructing marine sedimentary methane fluxes. Geochim. Cosmochim. Acta 354, 74–87. doi: 10.1016/j.gca.2023.06.008

[ref52] Kleindienst S. Ramette A. Amann R. Knittel K. (2012). Distribution and in situ abundance of sulfate-reducing bacteria in diverse marine hydrocarbon seep sediments. Environ. Microbiol. 14, 2689–2710. doi: 10.1111/j.1462-2920.2012.02832.x, 22882476

[ref53] Knittel K. Boetius A. (2009). Anaerobic oxidation of methane: Progress with an unknown process. Ann. Rev. Microbiol. 63, 311–334. doi: 10.1146/annurev.micro.61.080706.093130, 19575572

[ref54] Knittel K. Lösekann T. Boetius A. Kort R. Amann R. (2005). Diversity and distribution of Methanotrophic Archaea at cold seeps. Appl. Environ. Microbiol. 71, 467–479. doi: 10.1128/AEM.71.1.467-479.2005, 15640223 PMC544223

[ref55] Kohl S. D. Rice J. A. (1999). Contribution of lipids to the nonlinear sorption of polycyclic aromatic hydrocarbons to soil organic matter. Org. Geochem. 30, 929–936.

[ref56] Könneke M. Bernhard A. E. de la Torre J. R. Walker C. B. Waterbury J. B. Stahl D. A. (2005). Isolation of an autotrophic ammonia-oxidizing marine archaeon. Nature 437, 543–546. doi: 10.1038/nature03911, 16177789

[ref57] Kurth J. M. Smit N. T. Berger S. Schouten S. Jetten M. S. M. Welte C. U. (2019). Anaerobic methanotrophic archaea of the ANME-2d clade feature lipid composition that differs from other ANME archaea. FEMS Microbiol. Ecol. 95, 1–11. doi: 10.1093/femsec/fiz082, 31150548 PMC6581649

[ref58] Kvenvolden K. A. Cooper C. K. (2003). Natural seepage of crude oil into the marine environment. Geo-Mar. Lett. 23, 140–146. doi: 10.1007/s00367-003-0135-0

[ref59] Kvenvolden K. A. Rogers B. W. (2005). Gaia’s breath—global methane exhalations. Mar. Pet. Geol. 22, 579–590. doi: 10.1016/j.marpetgeo.2004.08.004

[ref60] LaJolo F. M. Tannenbaum S. R. Labuza T. P. (1971). Reaction at limited water concentration. 2. Chlorophyll degradation. J. Food Sci. 13, 281–286.

[ref61] Langworthy T. A. Holzer G. Zeikus J. G. Tornabene T. G. (1983). Iso- and Anteiso-branched glycerol Diethers of the thermophilic anaerobe Thermodesulfotobacterium commune. Syst. Appl. Microbiol. 4, 1–17. 23196295 10.1016/S0723-2020(83)80029-0

[ref62] Levin L. A. (2005). “Ecology of cold seep sediments: Interactions of Fauna with flow, chemistry and microbes” in Oceanography and marine biology. eds. Gibson R. N. Atkinson R. J. A. Gordon J. D. M. (CRC Press).

[ref63] Lipp J. S. Hinrichs K.-U. (2009). Structural diversity and fate of intact polar lipids in marine sediments. Geochim. Cosmochim. Acta 73, 6816–6833. doi: 10.1016/j.gca.2009.08.003

[ref64] Lipp J. S. Morono Y. Inagaki F. Hinrichs K. -U. (2008). Significant contribution of Archaea to extant biomass in marine subsurface sediments. Nature 454, 991–994. doi: 10.1038/nature07174, 18641632

[ref65] Liu X. -L. Lipp J. S. Schröder J. M. Summons R. E. Hinrichs K.-U. (2012). Isoprenoid glycerol dialkanol diethers: a series of novel archaeal lipids in marine sediments. Org. Geochem. 43, 50–55. doi: 10.1016/j.orggeochem.2011.11.002

[ref66] Liu X. L. Russell D. A. Bonfio C. Summons R. E. (2018). Glycerol configurations of environmental GDGTs investigated using a selective sn2 ether cleavage protocol. Org. Geochem. 128, 57–62. doi: 10.1016/j.orggeochem.2018.12.003

[ref67] Liu X. -L. Zhu C. Wakeham S. G. Hinrichs K. -U. (2014). In situ production of branched glycerol dialkyl glycerol tetraethers in anoxic marine water columns. Mar. Chem. 166, 1–8. doi: 10.1016/j.marchem.2014.08.008

[ref68] Luo M. Chen L. Wang S. Yan W. Wang H. Chen D. (2013). Pockmark activity inferred from pore water geochemistry in shallow sediments of the pockmark field in southwestern Xisha uplift, northwestern South China Sea. Mar. Pet. Geol. 48, 247–259. doi: 10.1016/j.marpetgeo.2013.08.018

[ref69] Lv Y. Yang S. Xiao X. Zhang Y. (2022). Stimulated organic carbon cycling and microbial community shift driven by a simulated cold-seep eruption. MBio 13, e00087–e00022. doi: 10.1128/mbio.00087-22, 35229641 PMC8941925

[ref70] Meador T. B. Zhu C. Elling F. J. Könneke M. Hinrichs K.-U. (2014). Identification of isoprenoid glycosidic glycerol dibiphytanol diethers and indications for their biosynthetic origin. Org. Geochem. 69, 70–75. doi: 10.1016/j.orggeochem.2014.02.005

[ref71] Meyers P. A. (1994). Preservation of elemental and isotopic source identification of sedimentary organic matter. Chem. Geol. 114, 289–302.

[ref72] Mitrović D. Hopmans E. C. Bale N. J. Richter N. Amaral-Zettler L. A. Baxter A. J. . (2023). Isoprenoidal GDGTs and GDDs associated with anoxic lacustrine environments. Org. Geochem. 178:104582. doi: 10.1016/j.orggeochem.2023.104582

[ref73] Murseli S. Middlestead P. St-Jean G. Zhao X. Jean C. Crann C. A. . (2019). The preparation of water (DIC, DOC) and gas (CO _2_, CH_4_) samples for radiocarbon analysis at AEL-AMS, Ottawa. Canada. Radiocarbon 61, 1563–1571. doi: 10.1017/RDC.2019.14

[ref74] Niemann H. Elvert M. (2008). Diagnostic lipid biomarker and stable carbon isotope signatures of microbial communities mediating the anaerobic oxidation of methane with sulphate. Org. Geochem. 39, 1668–1677. doi: 10.1016/j.orggeochem.2007.11.003

[ref75] Niemann H. Lösekann T. De Beer D. Elvert M. Nadalig T. Knittel K. . (2006). Novel microbial communities of the Haakon Mosby mud volcano and their role as a methane sink. Nature 443, 854–858. doi: 10.1038/nature05227, 17051217

[ref76] Niu M. Fan X. Zhuang G. Liang Q. Wang F. (2017). Methane-metabolizing microbial communities in sediments of the Haima cold seep area, northwest slope of the South China Sea. FEMS Microbiol. Ecol. 93, 1–13. doi: 10.1093/femsec/fix101, 28934399

[ref77] Oba M. Susumu S. Tetsuya F. (2015). Archaeal polar lipids in subseafloor sediments from the Nankai trough: implications for the distribution of methanogens in the deep marine subsurface. Org. Geochem. 78, 153–160. doi: 10.1016/j.orggeochem.2014.11.006

[ref78] Orcutt B. N. Joye S. B. Kleindienst S. Knittel K. Ramette A. Reitz A. . (2010). Impact of natural oil and higher hydrocarbons on microbial diversity, distribution, and activity in Gulf of Mexico cold-seep sediments. Deep-Sea Res. II Top. Stud. Oceanogr. 57, 21–23. doi: 10.1016/j.dsr2.2010.05.014

[ref79] Orcutt B. N. LaRowe D. E. Biddle J. F. Colwell F. S. Glazer B. T. Reese B. K. . (2013). Microbial activity in the marine deep biosphere: Progress and prospects. Front. Microbiol. 4, 1–15. doi: 10.3389/fmicb.2013.00189, 23874326 PMC3708129

[ref80] Orphan V. J. Hinrichs K. -U. Ussler W. Paull C. K. Taylor L. T. Sylva S. P. . (2001). Comparative analysis of methane-oxidizing Archaea and sulfate-reducing Bacteria in anoxic marine sediments. Appl. Environ. Microbiol. 67, 1922–1934. doi: 10.1128/AEM.67.4.1922-1934.2001, 11282650 PMC92814

[ref81] Orphan V. J. House C. H. Hinrichs K. -U. McKeegan K. D. DeLong E. F. (2002). Multiple archaeal groups mediate methane oxidation in anoxic cold seep sediments. Proc. Natl. Acad. Sci. 99, 7663–7668. doi: 10.1073/pnas.072210299, 12032340 PMC124316

[ref82] Peterse F. Kim J. -H. Schouten S. Kristensen D. K. Koç N. Sinninghe Damsté J. S. (2009). Constraints on the application of the MBT/CBT palaeothermometer at high latitude environments (Svalbard, Norway). Org. Geochem. 40, 692–699. doi: 10.1016/j.orggeochem.2009.03.004

[ref83] Pitcher A. Hopmans E. C. Mosier A. C. Park S.-J. Rhee S.-K. Francis C. A. . (2011). Core and intact polar glycerol Dibiphytanyl glycerol Tetraether lipids of Ammonia-oxidizing Archaea enriched from marine and estuarine sediments. Appl. Environ. Microbiol. 77, 3468–3477. doi: 10.1128/AEM.02758-10, 21441324 PMC3126447

[ref84] Pop Ristova P. Wenzhöfer F. Ramette A. Felden J. Boetius A. (2015). Spatial scales of bacterial community diversity at cold seeps (eastern Mediterranean Sea). ISME J. 9, 1306–1318. doi: 10.1038/ismej.2014.217, 25500510 PMC4438319

[ref85] Reeburgh W. S. (2007). Oceanic methane biogeochemistry. Chem. Rev. 107, 486–513. doi: 10.1021/cr050362v 17261072

[ref86] Regnier P. Dale A. W. Arndt S. LaRowe D. E. Mogollón J. Van Cappellen P. (2011). Quantitative analysis of anaerobic oxidation of methane (AOM) in marine sediments: a modeling perspective. Earth Sci. Rev. 106, 105–130. doi: 10.1016/j.earscirev.2011.01.002

[ref87] Reitz A. Pape T. Haeckel M. Schmidt M. Berner U. Scholz F. . (2011). Sources of fluids and gases expelled at cold seeps offshore Georgia, eastern Black Sea. Geochim. Cosmochim. Acta 75, 3250–3268. doi: 10.1016/j.gca.2011.03.018

[ref88] Riedinger N. (2017). Sulfur cycling in an iron oxide-dominated, dynamic marine depositional system: the argentine continental margin. Front. Earth Sci. 5:33. doi: 10.3389/feart.2017.00033

[ref89] Rooze J. Peterson L. Peterson R. N. Meile C. (2020). Porewater flow patterns in surficial cold seep sediments inferred from conservative tracer profiles and early diagenetic modeling. Chem. Geol. 536:119468. doi: 10.1016/j.chemgeo.2020.119468

[ref90] Rossel P. E. Elvert M. Ramette A. Boetius A. Hinrichs K.-U. (2011). Factors controlling the distribution of anaerobic methanotrophic communities in marine environments: evidence from intact polar membrane lipids. Geochim. Cosmochim. Acta 75, 164–184. doi: 10.1016/j.gca.2010.09.031

[ref91] Rossel P. E. Lipp J. S. Fredricks H. F. Arnds J. Boetius A. Elvert M. . (2008). Intact polar lipids of anaerobic methanotrophic archaea and associated bacteria. Org. Geochem. 39, 992–999. doi: 10.1016/j.orggeochem.2008.02.021

[ref92] Rütters H. Sass H. Cypionka H. Rullkötter J. (2002). Phospholipid analysis as a tool to study complex microbial communities in marine sediments. J. Microbiol. Methods 48, 149–160. doi: 10.1016/S0167-7012(01)00319-0, 11777565

[ref93] Rydzyński D. Piotrowicz-Cieślak A. I. Grajek H. . (2019). Chlorophyll degradation by tetracycline and cadmium in spinach (*Spinacia oleracea* L.) leaves. Int. J. Environ. Sci. Technol. 16, 6301–6314. doi: 10.1007/s13762-018-2142-8

[ref94] Schippers A. Neretin L. N. (2006). Quantification of microbial communities in near-surface and deeply buried marine sediments on the Peru continental margin using real-time PCR. Environ. Microbiol. 8, 1251–1260. doi: 10.1111/j.1462-2920.2006.01019.x, 16817933

[ref95] Schouten S. Hoefs M. J. L. Koopmans M. P. Bosch H. -J. Sinninghe Damsté J. S. (1998). Structural characterization, occurrence and fate of archaeal ether-bound acyclic and cyclic biphytanes and corresponding diols in sediments. Org. Geochem. 29, 1305–1319.

[ref96] Schouten S. Middelburg J. J. Hopmans E. C. Sinninghe Damsté J. S. (2010). Fossilization and degradation of intact polar lipids in deep subsurface sediments: a theoretical approach. Geochim. Cosmochim. Acta 74, 3806–3814. doi: 10.1016/j.gca.2010.03.029

[ref97] Schubotz F. Wakeham S. G. Lipp J. S. Fredricks H. F. Hinrichs K. (2009). Detection of microbial biomass by intact polar membrane lipid analysis in the water column and surface sediments of the Black Sea. Environ. Microbiol. 11, 2720–2734. doi: 10.1111/j.1462-2920.2009.01999.x, 19624710

[ref98] Schulz H. D. (2000). “Quantification of early diagenesis: dissolved constituents in marine pore water” in Marine Geochemistry. eds. Schulz H. D. Zabel M. (Berlin Heidelberg: Springer), 85–128.

[ref99] Simpson E. H. (1949). Measurement of diversity. Nature 163, 688–688.

[ref100] Sinninghe Damsté J. S. Hopmans E. C. Pancost R. D. Schouten S. Geenevasen J. A. J. (2000). Newly discovered non-isoprenoid glycerol dialkylglycerol tetraether lipids in sediments. Chem. Commun. 17, 1683–1684. doi: 10.1039/B004517I

[ref101] Solomon E. A. Kastner M. Jannasch H. Robertson G. Weinstein Y. (2008). Dynamic fluid flow and chemical fluxes associated with a seafloor gas hydrate deposit on the northern Gulf of Mexico slope. Earth Planet. Sci. Lett. 270, 95–105. doi: 10.1016/j.epsl.2008.03.024

[ref102] Sommer S. Pfannkuche O. Linke P. Luff R. Greinert J. Drews M. . (2006). Efficiency of the benthic filter: biological control of the emission of dissolved methane from sediments containing shallow gas hydrates at hydrate ridge. Glob. Biogeochem. Cycles 20, 1–14. doi: 10.1029/2004GB002389

[ref103] Stock L. Wegener G. Wang Y. Zander Y. Elvert M. (2025). Marine cold seep ANME-2/SRB consortia produce their lipid biomass from inorganic carbon. Environ. Microbiol. 27:e70213. doi: 10.1111/1462-2920.70213, 41327581 PMC12669819

[ref104] Sturt H. F. Summons R. E. Smith K. Elvert M. Hinrichs K. (2004). Intact polar membrane lipids in prokaryotes and sediments deciphered by high-performance liquid chromatography/electrospray ionization multistage mass spectrometry—new biomarkers for biogeochemistry and microbial ecology. Rapid Commun. Mass Spectrom. 18, 617–628. doi: 10.1002/rcm.1378, 15052572

[ref105] Suess E. (2020). “Marine cold seeps: background and recent advances” in Hydrocarbons, oils and lipids: Diversity, origin, chemistry and fate. ed. Wilkes H. (Springer International Publishing), 747–767. doi: 10.1007/978-3-319-54529-5_27-1

[ref106] Sultan N. Garziglia S. Ruffine L. (2016). New insights into the transport processes controlling the sulfate-methane-transition-zone near methane vents. Sci. Rep. 6:26701. doi: 10.1038/srep26701, 27230887 PMC4882613

[ref107] Sun T. (2023). The effects of organic matter and anaerobic oxidation of methane on the microbial sulfate reduction in cold seeps. Front. Mar. Sci. 10:1111133. doi: 10.3389/fmars.2023.1111133

[ref108] Sun M. Y. Lee C. Aller R. C. (1995). Erratum: laboratory studies of oxic and anoxic degradation of chlorophyll-a in Long Island sediments. Geochim. Cosmochim. Acta 59, 1439–1439.

[ref109] Talukder A. R. (2012). Review of submarine cold seep plumbing systems: leakage to seepage and venting. Terra Nova 24, 255–272. doi: 10.1111/j.1365-3121.2012.01066.x

[ref110] Teske A. Hinrichs K. -U. Edgcomb V. De Vera Gomez A. Kysela D. Sylva S. P. . (2002). Microbial diversity of hydrothermal sediments in the Guaymas Basin: evidence for anaerobic Methanotrophic communities. Appl. Environ. Microbiol. 68, 1994–2007. doi: 10.1128/AEM.68.4.1994-2007.2002, 11916723 PMC123873

[ref111] Thomas G. Tornabene Thomas A. Langworthy (1979). Diphytanyl and Dibiphytanyl glycerol ether lipids of methanogenic Archaebacteria. Science 203, 51–53. 758677 10.1126/science.758677

[ref112] Treude T. (2014). Sulfate reduction and methane oxidation activity below the sulfate-methane transition zone in Alaskan Beaufort Sea continental margin sediments: implications for deep sulfur cycling. Geochim. Cosmochim. Acta 144, 217–237. doi: 10.1016/j.gca.2014.08.018

[ref113] Umoh U. U. Li L. Luckge A. Schwarz-Schampera U. Naafs D. (2020). Influence hydrothermal vent activity on GDGT pool in marine sediments might be less than previously thought. Org. Geochem. 149:104102. doi: 10.1016/j.orggeochem.2020.104102

[ref114] Valdez-Nuñez L. F. Kappler A. Ayala-Muñoz D. Chávez I. J. Mansor M. (2024). Acidophilic sulphate-reducing bacteria: diversity, ecophysiology, and applications. Environ. Microbiol. Rep. 16:e70019. doi: 10.1111/1758-2229.70019, 39396517 PMC11471286

[ref116] Weemaes C. Ooms V. Van Loey A. M. Hendrickx M. E. (1999). Kinetics of chlorophyll degradation and color loss in heated broccoli juice. J. Agric. Food Chem. 47, 2404–2409. 10794643 10.1021/jf980663o

[ref117] Wegener G. Kellermann M. Y. Elvert M. (2016). Tracking activity and function of microorganisms by stable isotope probing of membrane lipids. Curr. Opin. Biotechnol. 41, 43–52. doi: 10.1016/j.copbio.2016.04.022, 27179643

[ref118] Weijers J. W. H. Schouten S. Hopmans E. C. Geenevasen J. A. J. David O. R. P. Coleman J. M. . (2006). Membrane lipids of mesophilic anaerobic bacteria thriving in peats have typical archaeal traits. Environ. Microbiol. 8, 648–657. doi: 10.1111/j.1462-2920.2005.00941.x 16584476

[ref119] Welte C. U. Rasigraf O. Vaksmaa A. Versantvoort W. Arshad A. Op den Camp H. J. M. . (2016). Nitrate- and nitrite-dependent anaerobic oxidation of methane. Environ. Microbiol. Rep. 8, 941–955. doi: 10.1111/1758-2229.12487 27753265

[ref120] Werne J. P. Haese R. R. Zitter T. Aloisi G. Bouloubassi I. Heijs S. . (2004). Life at cold seeps: a synthesis of biogeochemical and ecological data from Kazan mud volcano, eastern Mediterranean Sea. Chem. Geol. 205, 367–390. doi: 10.1016/j.chemgeo.2003.12.031

[ref121] White D. C. Davis W. M. Nickels J. S. King J. D. Bobbie R. J. (1979). Determination of the sedimentary microbial biomass by extractible lipid phosphate. Oecologia 40, 51–62. 28309603 10.1007/BF00388810

[ref122] Whiticar M. J. (1999). Carbon and hydrogen isotope systematics of bacterial formation and oxidation of methane. Chem. Geol. 161, 291–314.

[ref123] Wörmer L. Lipp J. S. Hinrichs K. -U. (2015). “Comprehensive analysis of microbial lipids in environmental samples through HPLC-MS protocols” in Hydrocarbon and lipid microbiology protocols. eds. McGenity T. J. Timmis K. N. Nogales B. (Berlin Heidelberg: Springer), 289–317.

[ref124] Woulds C. Cowie G. L. (2009). Sedimentary pigments on the Pakistan margin: controlling factors and organic matter dynamics. Deep-Sea Res. II 56, 347–357. doi: 10.1016/J.DSR2.2008.05.033

[ref125] Xiao W. Wang Y. Zhou S. Hu L. Yang H. Xu Y. (2016). Ubiquitous production of branched glycerol dialkyl glycerol tetraethers (brGDGTs) in global marine environments: a new source indicator for brGDGTs. Biogeosciences 13, 5883–5894. doi: 10.5194/bg-13-5883-2016

[ref126] Xiao W. Xu Y. Lin J. Zeng Z. Liu Y. Zhang H. . (2022). Global scale production of brGDGTs by benthic marine bacteria: implication for developing ocean bottom environmental proxies. Glob. Planet. Chang. 211:103783. doi: 10.1016/j.gloplacha.2022.103783

[ref127] Yanagawa K. Sunamura M. Lever M. A. Morono Y. Hiruta A. Ishizaki O. . (2011). Niche separation of methanotrophic archaea (ANME-1 and -2) in methane-seep sediments of the eastern Japan Sea offshore Joetsu. Geomicrobiol J. 28, 118–129. doi: 10.1038/ismej.2012.124

[ref128] Yunker M. B. Belicka L. L. Harvey H. R. Macdonald R. W. (2005). Tracing the inputs and fate of marine and terrigenous organic matter in Arctic Ocean sediments: a multivariate analysis of lipid biomarkers. Deep-Sea Res. II Top. Stud. Oceanogr. 52, 3478–3508. doi: 10.1016/j.dsr2.2005.09.008

[ref129] Zeebe R. E. Wolf-Gladrow D. (2001). “Chapter 1 Equilibrium” in Elsevier oceanography series, vol. 65 (Elsevier), 1–84.

[ref130] Zhang T. (2023). Lipidomic diversity and proxy implications of archaea from cold seep sediments of the South China Sea. Front. Microbiol. 14:1241958. doi: 10.3389/fmicb.2023.1241958 37954235 PMC10635418

[ref131] Zhang Z. -X. Li J. Chen Z. Sun Z. Yang H. Fu M. . (2020). The effect of methane seeps on the bacterial tetraether lipid distributions at the Okinawa trough. Mar. Chem. 225:103845. doi: 10.1016/j.marchem.2020.103845

[ref132] Zhang Z.-X. Li J. Lu H. Yang H. Zhang Y. Tang Y. . (2024). Bacterial glycerol tetraethers as a potential tool to trace marine methane cycling. Limnol. Oceanogr. 69, 104–120. doi: 10.1002/lno.12462

[ref133] Zhang Y. G. Zhang C. L. Liu X.-L. Li L. Hinrichs K. -U. Noakes J. E. (2011). Methane index: a tetraether archaeal lipid biomarker indicator for detecting the instability of marine gas hydrates. Earth Planet. Sci. Lett. 307, 525–534. doi: 10.1016/j.epsl.2011.05.031

[ref134] Zhao Y. Liu Y. Cao S. Hao Q. Liu C. Li Y. (2024). Anaerobic oxidation of methane driven by different electron acceptors: a review. Sci. Total Environ. 946:174287. doi: 10.1016/j.scitotenv.2024.174287, 38945238

[ref135] Zhong S. (2025). Sediment depth impacts microbial community structure in methane seepage regions. Commun. Earth Environ. 6:868. doi: 10.1038/s43247-025-02794-0

