# Supplementary material for: Microbial and geochemical architecture of an active Scotian Slope cold seep
Source: Front Microbiol. 2026 Feb 23;17:1709097. doi: 10.3389/fmicb.2026.1709097 (PMC12989751; doi:10.3389/fmicb.2026.1709097)
Supplement: Supplementary file 1 [file Presentation_1.PDF]

Microbial and Geochemical Architecture of an Active Scotian Slope Cold Seep

*Elish Redshaw[1], Gamra Oueslati[1], Natasha MacAdam[2], Unyime Umoh[1], Patricia Granados[3], Jeremy N. Bentley[1], Narges Ahangarian[1], Martin G. Fowler[4], Robbie Bennett[4], Venus Baghalabadi[1,5], Adam MacDonald[2], G. Todd Ventura[1]**

[1]Department of Geology, Saint Mary's University, 923 Robie Street, Halifax, Nova Scotia B3H 3C3, Canada

[2]Nova Scotia Department of Energy, 1690 Hollis St., Halifax, Nova Scotia B3J 3J9, Canada

[3]Centre for Environmental Analysis and Remediation, Saint Mary's University, 923 Robie Street, Halifax, Nova Scotia B3H 3C3, Canada

[4]Applied Petroleum Technology (Canada) Ltd., Calgary, AB T3A 2M3, Canada

[5]Department of Pharmacology, Dalhousie University, 5850 College St, Halifax, Nova Scotia, B3H 4R2, Canada

Supplementary Material

1 Supplementary Data

Supplementary Material should be uploaded separately on submission. Please include any supplementary data, figures and/or tables.

Supplementary material is not typeset so please ensure that all information is clearly presented, the appropriate caption is included in the file and not in the manuscript, and that the style conforms to the rest of the article.

2 Supplementary Figures and Tables

2.1 Supplementary Figures

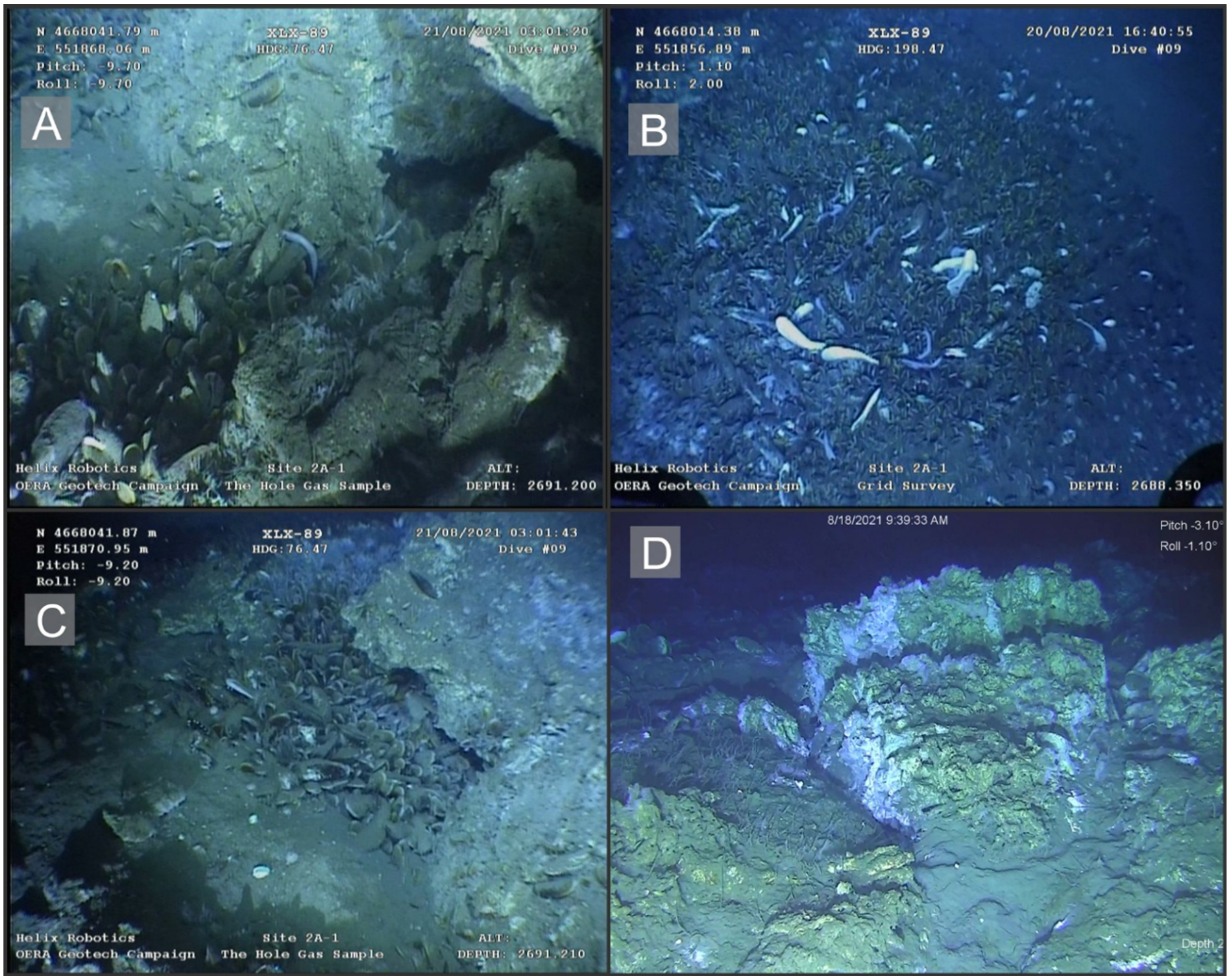

Figure S1: Seep photos taken from ROV camera (images from Bennett & Desiage, 2022).

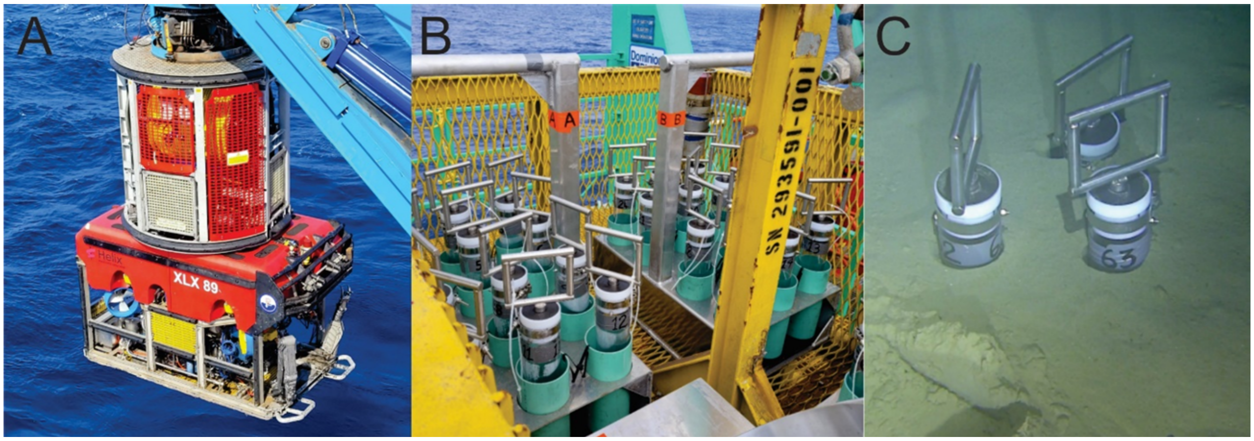

Figure S2: A) Triton XLX ROV pre-deployment. B) Push core quivers loaded for deployment. C) Push core sampling in progress. Images modified from (Bennett & Desiage, 2022).

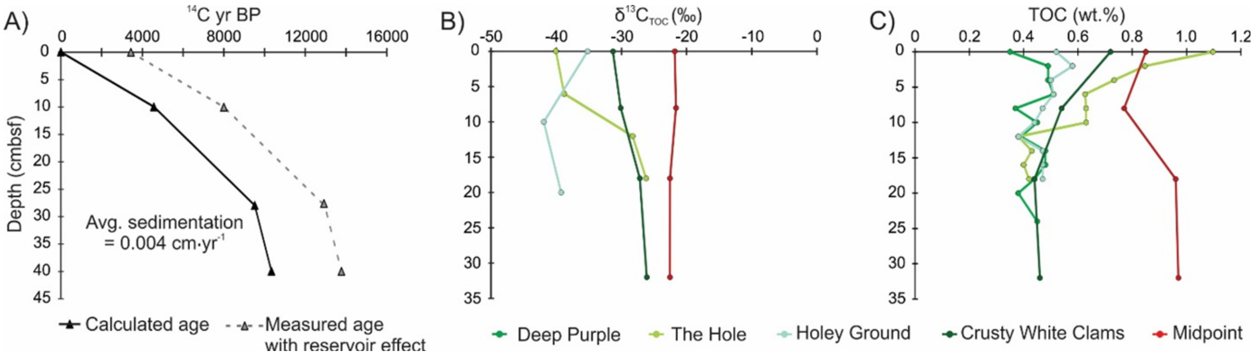

Figure S3: A) Sedimentation rate as determined from radiocarbon age data of core 2A-49. The rate is calculated from the reservoir effect adjusted ^{14}C age. B) Downcore profiles of $\delta^{13}C$ measurement.

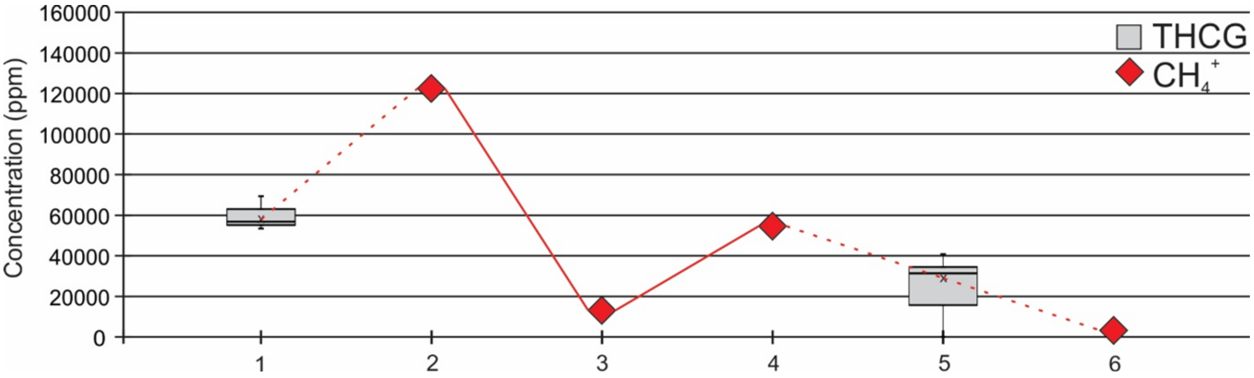

Figure S4: Methane (from Chowdhury et al., 2024) and total hydrocarbon gas (THCG; unpublished open-source file data supplied by Martin Fowler of Applied Petroleum Technologies) concentrations across the push core transect.

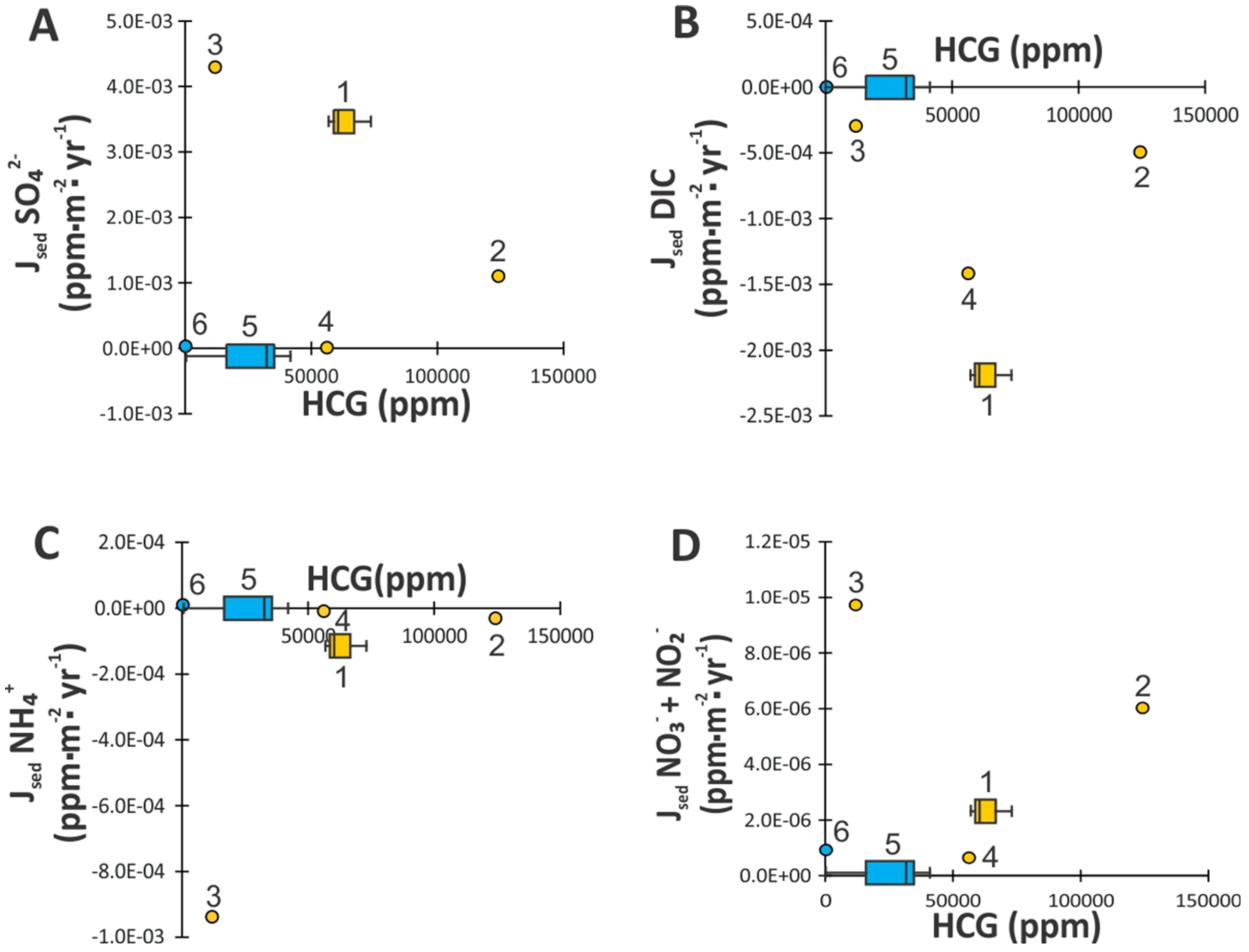

Figure S5: Cross-plots SO_4^{2-} and NH_4^+ ion diffusion flux versus HCG (Chowdhury et al., 2024) for seep cores (yellow) and transect cores (blue). Numerals next to the data point indicate the transect core number.

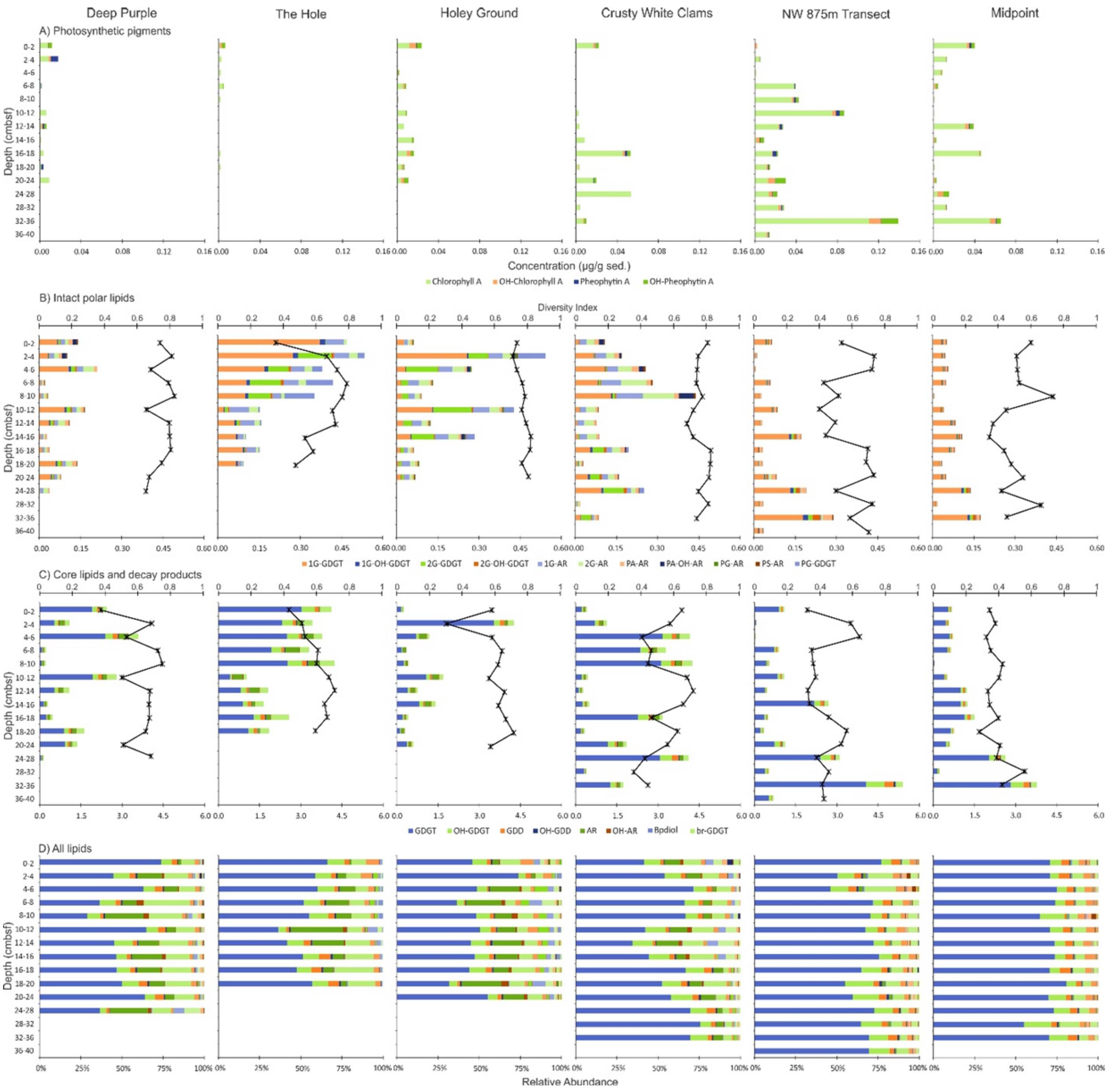

FIGURE S6: Down core profiles of the 2A-1 seep transect cores A) water column sourced pigments, B) IPLs, C) CLs and CL-DP, and D) the percent abundance of all lipids (black lines indicate Simpson's Diversity Index changes). Depth uniformly extends to a maximum depth of 20 cmbsf conforming to the shallowest core in the transect survey.

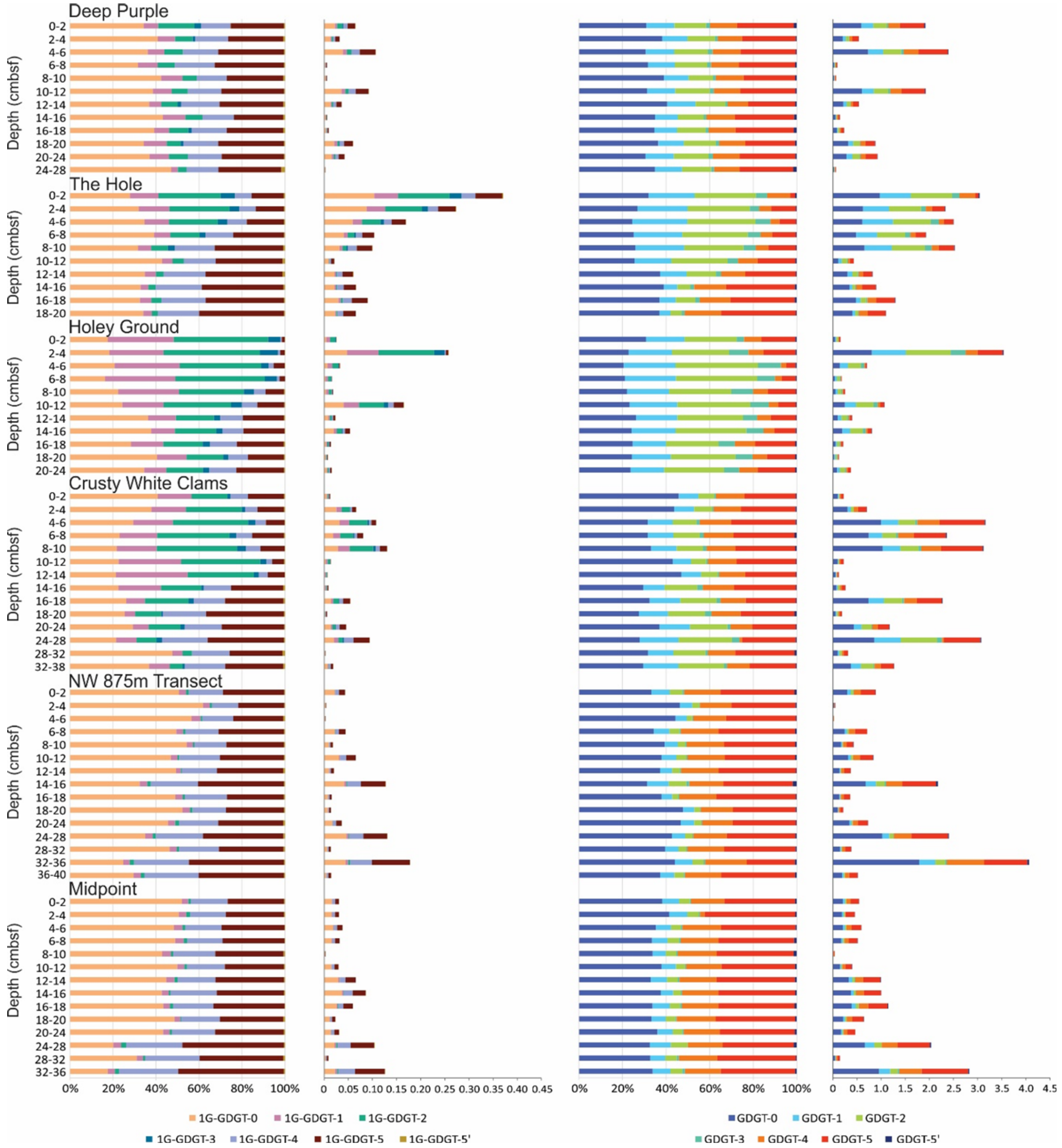

Figure S7: 1G-GDGT and GDGT relative abundance and concentrations across site 2A-1 transect.

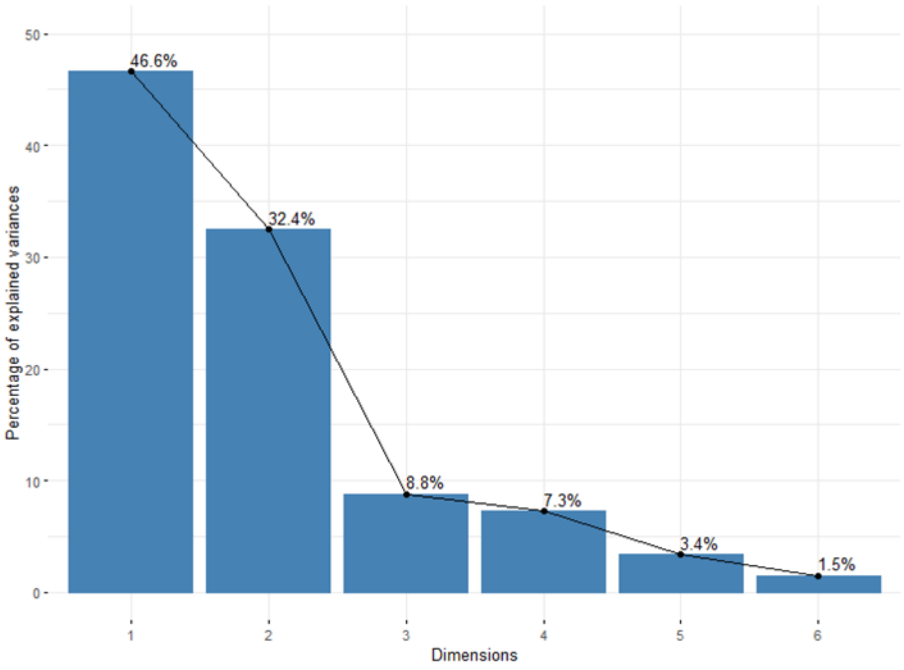

Figure S8: Scree plot of the porewater ion concentrations.

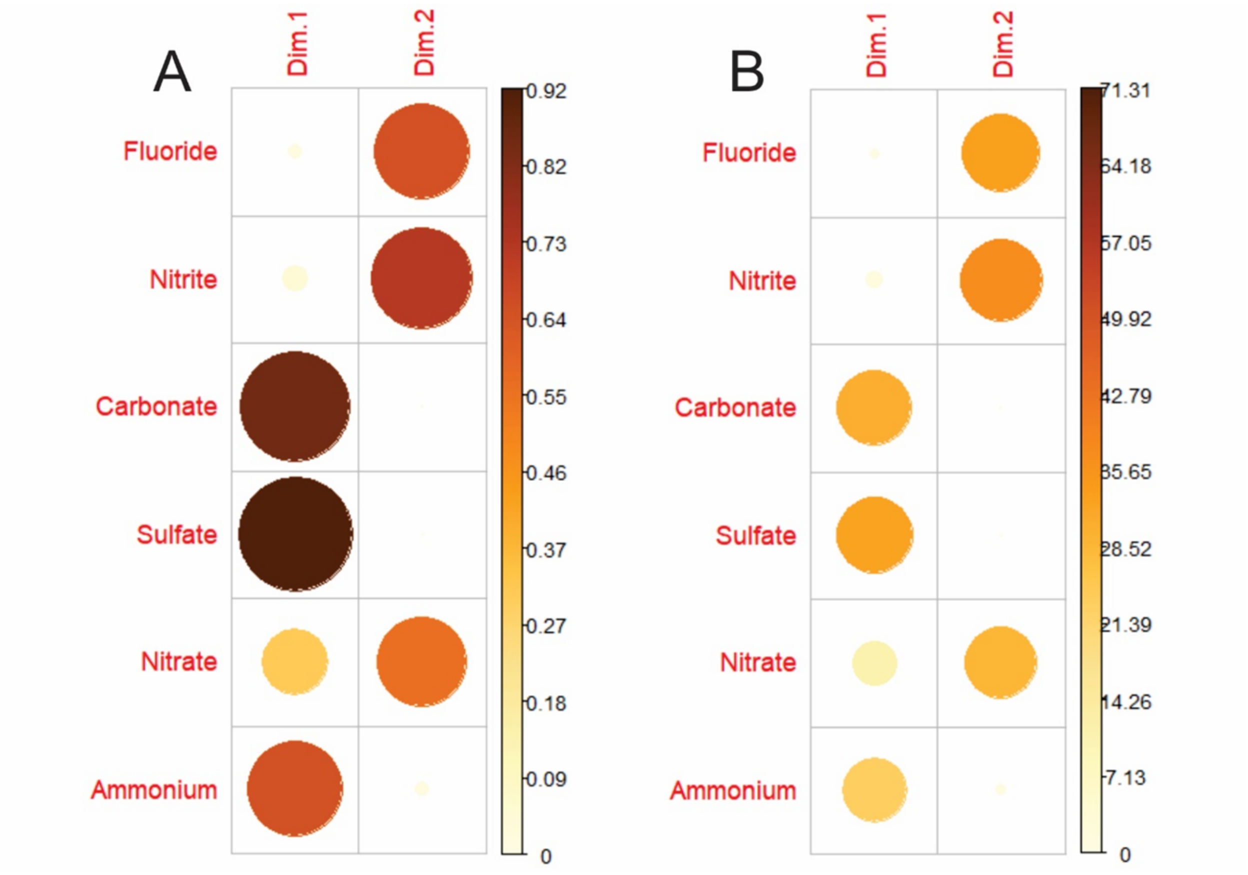

Figure S9: A) Cos2 values of each ion. B) Variable contribution values of each ion.

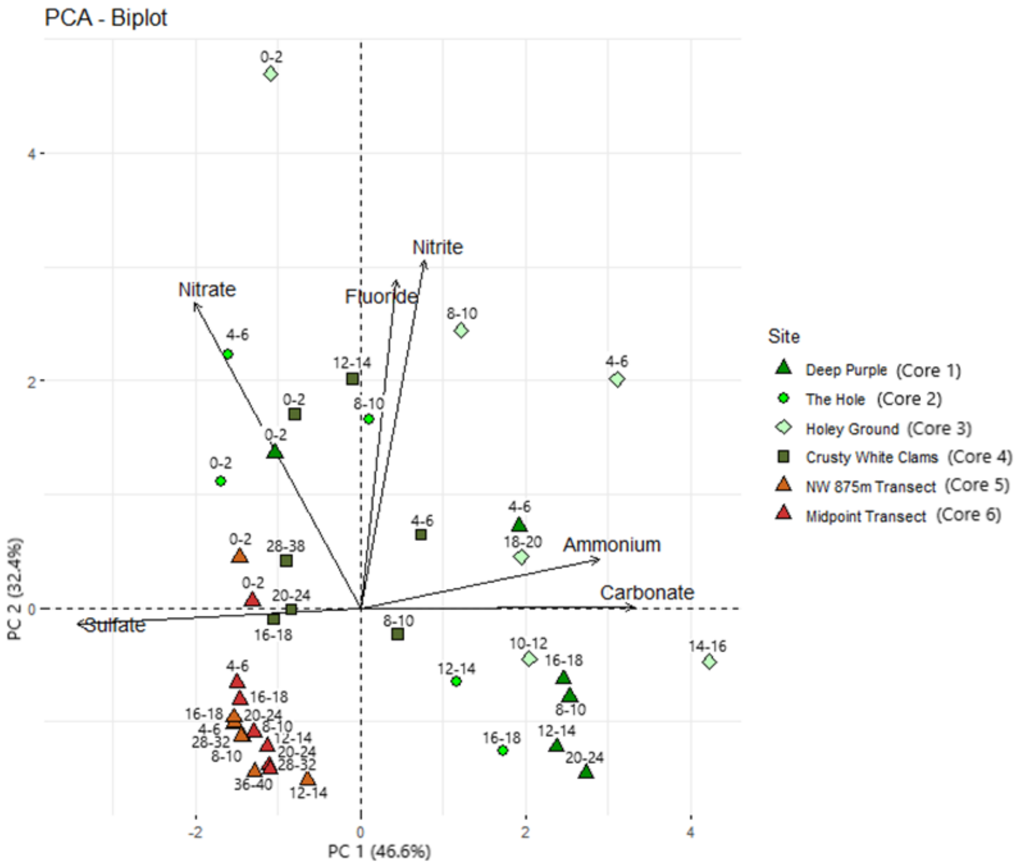

Figure S10: Depth annotated PCA biplot.

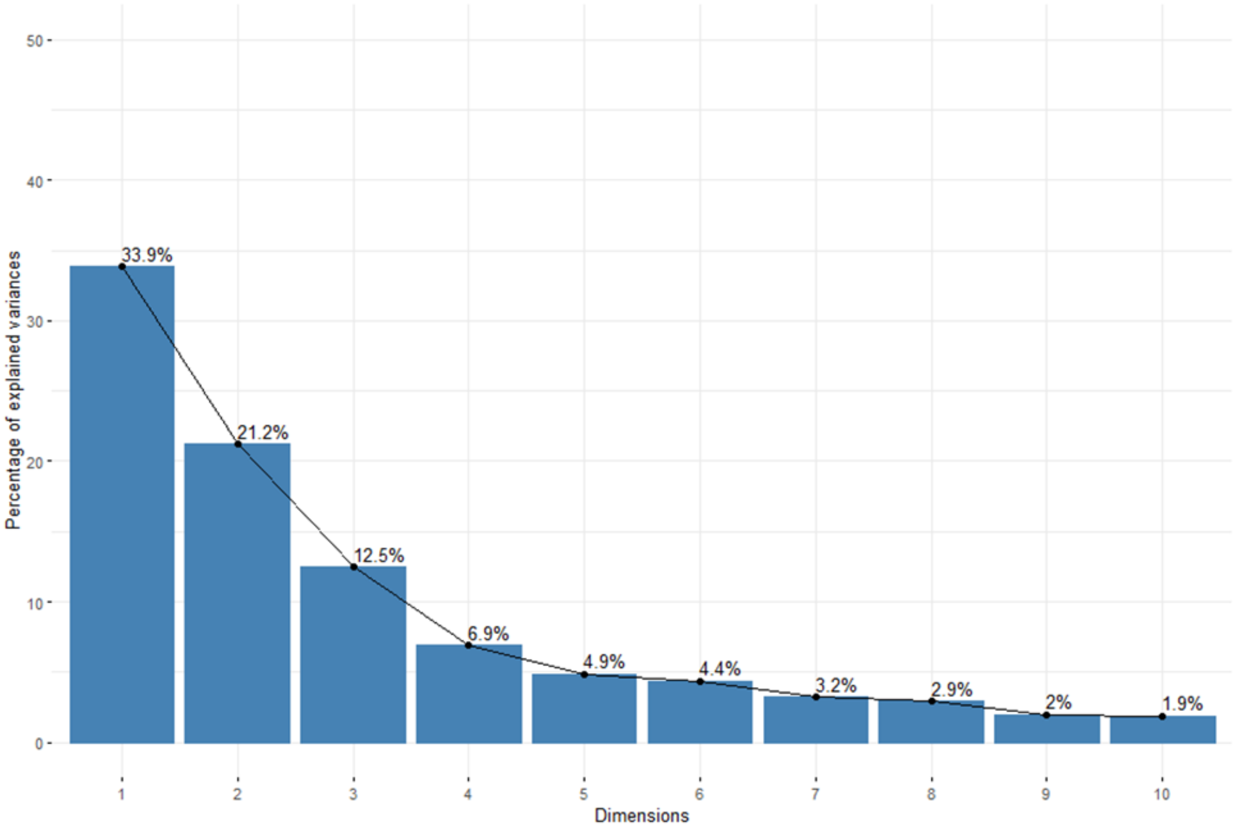

Figure S11: Scree plot of lipidomic PCA eigenvalues.

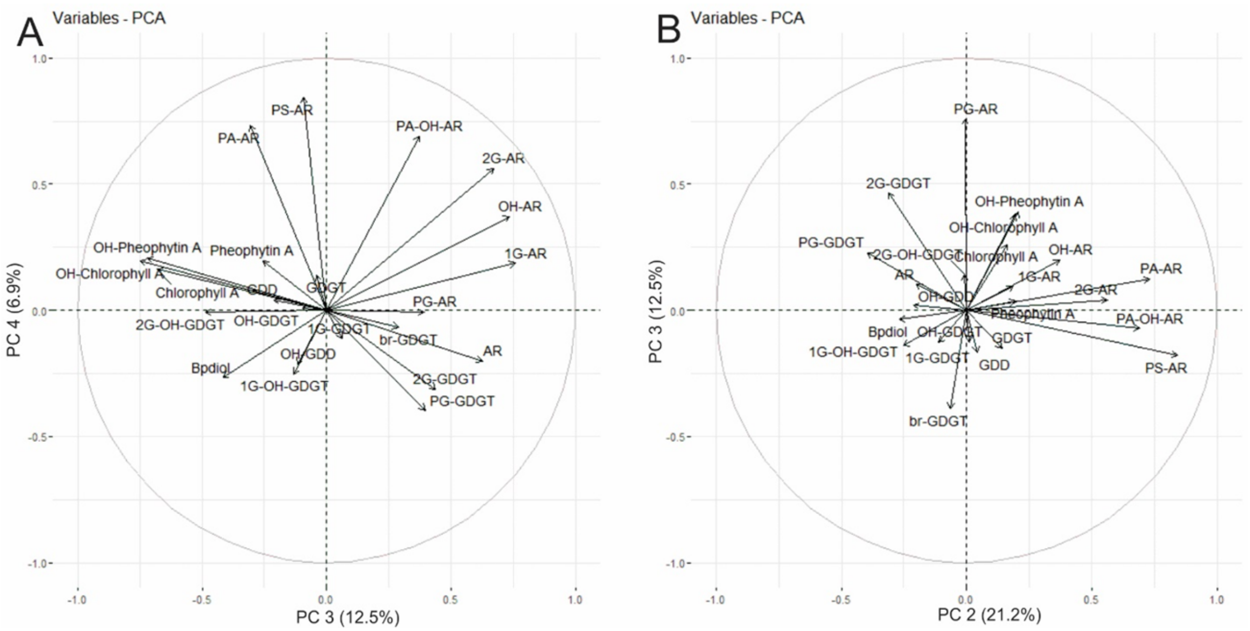

Figure S12: PCA imbedded factor loadings and scores plots for A) PC 2 and PC 3 and B) PC 3 and PC 4.

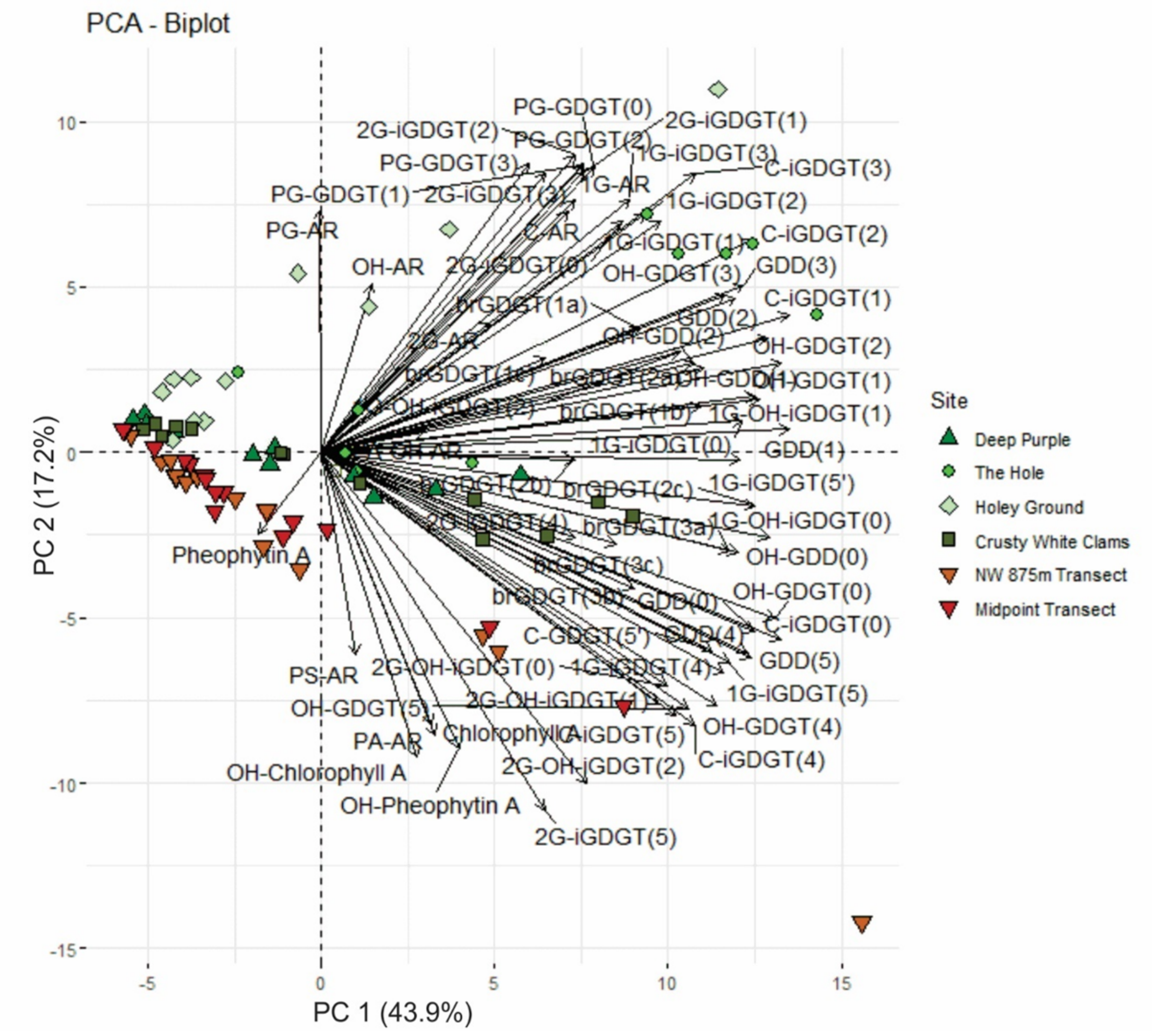

Figure S13: PCA biplot of lipid classes separated by ring structure.

2.2 Supplementary Tables

TABLE S1: Diffusion constants for free ions in seawater from Schulz (2000) and sources herein.

**Ion**	**D_{SW} ($m^2 \cdot s^{-1}$ at 5 °C)**
NO_2^-	1.13×10^{-9}
NO_3^-	1.08×10^{-9}
SO_4^{2-}	5.72×10^{-10}
DIC	6.09×10^{-10}
NH_4^+	1.10×10^{-9}

Supplemental Table S2: ^{14}C data provided by the André E. Lalonde AMS Laboratory (University of Ottawa).

**Sample name**	**^{14}C yr BP**	**±**	**$F^{14}C$**	**±**
**2A-49, 0-2**	3434	15	0.6521	0.0012
**2A-49, 10-12**	8005	20	0.3691	0.0009
**2A-49, 28-32**	12957	28	0.1993	0.0007
**2A-49, 40-46**	13777	31	0.1799	0.0007

TABLE S3: Porewater ion and TOC, PIC concentration summary*.

Core Number	Sediment Interval (cmbsf)	Instrument	Porosity ($ml \cdot ml^{-1}$)	TOC (wt.%)	$\delta^{13}C_{TOC}$ (‰)	PIC (wt.%)	Ions ($mmol \cdot L^{-1}$)								
							F^-	NO_2^-	DIC	SO_4^{2-}	NO_3^-	NH_4^+	Fe^{2+}	Mn^{2+}	PO_4^{3-}
1	0-2	IC + P	0.14	0.35		0.63	0.24	0.07	2.50	23.12	0.17	0.34	0.00	U	U
	2-4	P		0.49		0.63	-	-	-	-	-	0.94	0.01	0.08	0.04
	4-6	IC	0.12	0.49		0.60	0.33	0.07	11.97	6.81	0.12	-	-	-	-
	6-8	P	0.10	0.51		0.66	-	-	-	-	-	2.88	0.04	0.20	0.08
	8-10	IC	0.09	0.37		0.55	0.13	0.06	14.83	2.09	0.11	-	-	-	-
	10-12	P	0.08	0.45		0.58	-	-	-	-	-	1.13	0.02	0.06	0.05
	12-14	IC	0.08	0.39		0.58	0.11	0.05	15.58	1.59	0.12	-	-	-	-
	14-16	P		0.48		0.56	-	-	-	-	-	2.68	0.02	0.11	0.07
	16-18	IC		0.48		0.62	0.15	0.06	13.34	1.13	0.12	-	-	-	-
	18-20	P	0.08	0.44		0.57	-	-	-	-	-	2.45	0.01	0.18	0.07
	20-24	IC	0.03	0.38		0.67	0.11	0.05	15.78	1.15	0.10	-	-	-	-
	24-28	P	0.01	0.45		0.63	-	-	-	-	-	2.16	0.01	0.11	0.07
2	0-2	IC + P	0.01	1.10	-40.06	0.40	0.17	0.07	0.06	28.72	0.17	0.25	0.00	0.07	0.07
	2-4	P	0.01	0.85		0.46	-	-	-	-	-	0.29	0.02	0.07	0.02
	4-6	IC		0.73		0.39	0.18	0.09	0.82	27.56	0.18	-	-	-	-
	6-8	P	0.12	0.63	-38.80	0.43	-	-	-	-	-	0.09	0.00	U	U
	8-10	IC	0.09	0.63		0.33	0.21	0.09	4.54	15.61	0.14	-	-	-	-
	10-12	P	0.07	0.63		0.30	-	-	-	-	-	0.44	U	U	U
	12-14	IC	0.05	0.38	-28.29	0.40	0.12	0.06	10.13	4.84	0.13	-	-	-	-
	14-16	P	0.03	0.43		0.29	-	-	-	-	-	0.67	U	U	U
	16-18	IC	0.02	0.40		0.29	0.09	0.06	11.72	1.29	0.11	-	-	-	-
	18-20	P	0.01	0.42	26.22	0.29	-	-	-	-	-	0.62	U	U	U
3	0-2	IC + P		0.52	-35.20	0.29	0.71	0.08	2.56	24.59	0.19	0.68	0.00	0.02	0.01
	2-4	P		0.58		0.36	-	-	-	-	-	0.67	0.01	0.02	0.02
	4-6	IC+P		0.50		0.31	0.32	0.08	12.66	3.48	0.14	5.64	0.01	U	0.10
	6-8			0.51		0.36	-	-	-	-	-	-	-	-	-
	8-10	IC	0.06	0.47		0.36	0.25	0.09	8.30	4.56	0.17	-	-	-	-

	10-12	IC+P	0.06	0.44	-41.92	0.36	0.18	0.07	13.18	0.87	0.11	0.39	0.01	0.10	0.00
	12-14	P		0.38		0.33	-	-	-	-	-	7.14	0.01	0.01	U
	14-16	IC		0.47		0.28	0.08	0.07	13.79	1.00	0.10	-	-	-	-
	16-18	P	0.12	0.47		0.34	-	-	-	-	-	7.14	0.01	U	0.10
	18-20	IC	0.06	0.47		0.30	0.18	0.06	10.01	4.64	0.14	-	-	-	-
	20-24	P	0.05		-39.25	0.36	-	-	-	-	-	0.43	0.01	0.07	0.01
	0-2	IC + P	0.05	0.72	-31.31	0.29	0.15	0.09	2.64	23.75	0.16	0.18	0.00	0.06	0.01
	2-4	P	0.05	0.37		0.21	-	-	-	-	-	0.55	0.03	0.13	0.01
	4-6	IC	0.05	0.37		0.23	0.13	0.07	18.53	22.47	0.15	-	-	-	-
	6-8	P	0.04			0.20	-	-	-	-	-	0.35	0.03	0.07	0.00
	8-10	IC		0.54	-30.13	0.23	0.12	0.06	8.98	11.70	0.14	-	-	-	-
	10-12	P	0.12	0.21		0.23	-	-	-	-	-	0.44	0.01	0.10	0.02
	12-14	IC	0.08			0.19	0.51	0.07	4.58	20.16	0.14	-	-	-	-
**4**	14-16	P	0.07	0.44		0.21	-	-	-	-	-	0.18	0.01	0.07	0.01
	16-18	IC	0.07	0.37		0.20	0.09	0.06	2.56	23.77	0.15	-	-	-	-
	18-20	P	0.06	0.44	-27.19	0.22	-	-	-	-	-	0.05	0.04	U	0.00
	20-24	IC	0.08	0.24		0.23	0.11	0.07	2.07	23.71	0.14	-	-	-	-
	24-28	P	0.08	0.17		0.20	-	-	-	-	-	0.11	0.01	0.20	U
	28-32	IC	0.14	0.16		0.18	0.10	0.07	3.11	22.77	0.15	-	-	-	-
	32-38	P		0.46	-26.12	0.22	-	-	-	-	-	0.14	0.00	U	U
	0-2	IC	0.12	0.48		0.50	0.07	0.08	0.22	28.63	0.15	-	-	-	-
	2-4	P	0.10	0.44		0.47	-	-	-	-	-	0.00	0.00	0.06	0.00
	4-6	IC	0.09	0.39		0.47	0.09	0.05	0.45	28.56	0.14	-	-	-	-
	6-8	P	0.08	0.43		0.46	-	-	-	-	-	0.09	0.00	0.07	0.01
	8-10	IC	0.08	0.52		0.45	0.10	0.05	1.00	29.89	0.14	-	-	-	-
**5**	10-12	P		1.12		0.49	-	-	-	-	-	0.04	0.00	0.08	0.00
	12-14	IC		0.44		0.43	0.09	0.05	1.80	21.20	0.12	-	-	-	-
	14-16	P	0.08	0.35		0.42	-	-	-	-	-	0.16	0.01	0.07	0.00
	16-18	IC	0.03	0.52		0.39	0.09	0.05	0.66	30.39	0.14	-	-	-	-
	18-20	P	0.01	0.48		0.28	-	-	-	-	-	0.11	0.00	0.11	0.00
	20-24	IC	0.01	0.52		0.31	0.10	0.05	1.06	30.25	0.14	-	-	-	-

	24-28	P	0.01	0.56		0.22	-	-	-	-	-	0.04	0.01	0.05	0.00
	28-32	IC		0.42		0.28	0.09	0.05	0.66	29.63	0.14	-	-	-	-
	32-36	P	0.12	0.49		0.27	-	-	-	-	-	0.14	0.00	0.05	0.00
	36-40	IC	0.09	0.48		0.23	0.10	0.05	0.60	29.78	0.13	-	-	-	-
	0-2	IC + P	0.07	0.85	-21.83	0.46	0.07	0.07	2.13	27.81	0.15	0.01	0.00	0.07	0.00
	2-4	P	0.05	0.50		0.46	-	-	-	-	-	0.08	0.00	0.05	0.00
	4-6	IC	0.03	0.36		0.50	0.07	0.05	0.88	29.62	0.15	-	-	-	-
	6-8	P	0.02	0.43		0.45	-	-	-	-	-	0.51	0.00	0.03	0.00
	8-10	IC	0.01	0.77	-21.63	0.46	0.08	0.05	0.55	28.18	0.13	-	-	-	-
	10-12	P				0.45	-	-	-	-	-	0.35	0.00	0.05	0.01
	12-14	IC				0.44	0.08	0.05	0.61	28.33	0.12	-	-	-	-
**6**	14-16	P				0.41	-	-	-	-	-	0.11	0.01	0.07	0.01
	16-18	IC				0.29	0.09	0.05	1.11	28.73	0.15	-	-	-	-
	18-20	P	0.06	0.96	-21.55	0.30	-	-	-	-	-	0.16	0.00	0.04	0.01
	20-24	IC	0.06			0.31	0.08	0.05	1.11	29.19	0.13	-	-	-	-
	24-28	P				0.28	-	-	-	-	-	0.12	0.00	U	0.01
	28-32	IC				0.40	0.09	0.05	0.55	27.72	0.12	-	-	-	-
	32-36	P	0.12	0.97	-22.56	0.30	-	-	-	-	-	0.14	0.00	0.05	0.01

* The measurement of porewater ions is based on porosity and sediment availability. As such, not all samples could be measured consistently downcore. Priority was therefore given to NH_4^+ followed by Fe^{2+}, with Mn^{2+}, and PO_4^{3-} being the lowest priority and only measured in the most porous sediments. Core 2 was extremely non-porous and limited ion measurements to IC measured anions and NH_4^+.

IC = ion chromatography

P = photometer

U = measurement unavailable from lack of porewater

Supplemental Table S4a: Archaeal lipids names and diagnostic mass spectral characteristics used for identification.

Chemical Name	Abbreviation	RT (min)	Molecular Ion $[M^{+}H]^{+}$	Diagnostic Fragment Ion	References
**Hydroxyl phosphatidic acid archaeol**	PA-OH-AR	4	749.6	453.3	Yoshinaga et al., 2011
**sn2-Hydroxyl Archaeol**	OH-AR	8	669.17	367, 341	
**Phosphatidic acid archaeol**	PA-AR	9	733.6	453.3, 357.4	Yoshinaga et al., 2011
**Phosphatidyl glycerol archaeol**	PG-AR	9	807.7	733.6, 653.7	Yoshinaga et al., 2011
**Biphytanediol**	Bp-Diol	10	595.6388, 593.6244, 591.6100, 589.5956		Schouten et al., 1998
**Diglycosidic Archaeol**	2G-AR	11	977.7862	653, 373	Lipp and Hinrichs, 2009; Yoshinaga et al., 2011
**Monoglycosidic Archaeol**	1G-AR	13	815.7334	653, 373	Lipp and Hinrichs, 2009; Yoshinaga et al., 2011
**Phosphatidylserine Archaeol**	PS-AR	14	803.6735	733.6, 453.3	Yoshinaga et al., 2011
**Archaeol**	AR	19	653.6806	373.3	Lipp and Hinrichs, 2009; Yoshinaga et al., 2011
**Hydroxylated glycerol dialkanol diether**	OH-GDD (0-2)	33	1262.2913, 1260.2757, 1258.2601	686.6	Liu et al., 2012a
**Phosphatidylglycerol glycerol dialkyl glycerol tetraether**	PG-GDGT	34	1456.3227, 1454.3070, 1452.2914, 1450.2757, 1448.2601, 1446.2444	1380.3, 1300.0	Yoshinaga et al., 2011
**Hydroxyl diglycosidic glycerol dialkyl glycerol tetraether**	2G-OH-GDGT-0; 2G-OH-GDGT-2	34	1642.4283, 1640.4075, 1638.3919	1318.3, 760.7, 668.7	Lipp and Hinrichs, 2009; Liu et al., 2012c
**Hydroxyl monoglycosidic glycerol dialkyl glycerol tetraether**	1G-OH-GDGT (0-2)	38	1480.3704, 1478.3548, 1476.3391	1318.3, 760.7, 668.7	Lipp and Hinrichs, 2009; Liu et al., 2012a
**Diglycosidic glycerol dialkyl glycerol tetraether**	2G-GDGT (0-5)	40	1626.4283, 1624.4127, 1622.3970,1620.3814, 1618.3657, 1616.3501	1302.3, 743.7, 651.7	Stuart et al., 2004; Meador et al., 2014

**Glycerol dialkanol diether**	GDD	41	1246.2965, 1244.2808, 1242.2652, 1240.2495, 1238.2339, 1236.2182	669.6	Liu et al., 2012b
**Hydroxyl Glycerol dialkyl glycerol tetraether**	OH-GDGT (0-5)	43	1318.3176, 1316.3019. 1314.2863, 1312.2706, 1310.2550, 1308.2393	1302.3, 743.7, 651.7	Liu et al., 2012c
**Monoglycosidic glycerol dialkyl glycerol tetraether**	1G-GDGT-0; 1G-GDGT-5+5’	44	1464.3755, 1462.3598, 1460.3442, 1458.3285 1456.3129, 1454.2972	1302.3, 743.7, 651.7	Stuart et al., 2004
**Glycerol dialkyl glycerol tetraether**	GDGT-0; GDGT-5+5’	54	1302.3226, 1300.3070, 1298.2914, 1296.2757, 1294.2601, 1292.2444	1302.3, 743.7, 651.7	Hopmans et al., 2000; Schouten et al., 2013; Lengger et al., 2018

Supplemental Table S4b: Eukaryotic lipid names and diagnostic mass spectral characteristics used for identification.

Chemical Name	Abbreviation	RT (min)	Molecular Ion $[M^{+}H]^{+}$	Diagnostic Fragment Ion	References
**Hydroxychlorophyll a**	OH-Chlo	14	909.5502	593.3, 533.5	Chen et al., 2015
**Hydroxypheophytin a**	OH-Pheo	15	887.5782	591, 609	Milenkovic et al., 2012; Chen et al., 2015
**Chlorophyll a**	Chl a	17	893.5422	593.3, 533.5	Milenkovic et al., 2012; Chen et al., 2015
**Pheophytin a**	Pheo a	18	871.5721	591, 609	Chen et al., 2015

Supplemental Table S4c: Bacterial and mixed source *br*GDGT mass spectral characteristics used for lipid identification.

Chemical Name	Abbreviation	RT (min)	Molecular Ion $[M^{+}H]^{+}$	Diagnostic Fragment Ion	References
**Branched glycerol dialkyl glycerol tetraether**	*br*GDGT	35	1050.0410, 1048.0253, 1046.0097, 1036.0253, 1034.0097, 1031.9940, 1022.0097, 1019.9921, 1017.9775	525.5, 489.6	Schouten et al, 2000, Hopmans et al., 2004

Supplemental Table S5: Deep Purple (core 1) downcore lipid concentration (μg·g^{-1} sed).

Sample Name	GDGT	OH-GDGT	GDD	OH-GDD	AR	OH-AR	Bpdiol	*br*GDGT	1G-GDGT	1G-OH-GDGT	2G-GDGT	2G-OH-GDGT	1G-AR	2G-AR	PA-AR	PA-OH-AR	PG-AR	PS-AR	PG-GDGT	Chlorophyll a	OH-Chlorophyll a	Pheophytin a	OH-Pheophytin a
**2A-1,36, 0-2**	1.92	0.17	0.09	0.01	0.05	0.00	-	0.21	0.06	0.00	0.01	0.00	0.02	0.01	0.02	0.01	-	0.00	-	0.01	0.00	0.00	0.00
**2A-1,36, 2-4**	0.54	0.12	0.04	0.01	0.18	0.02	0.00	0.17	0.03	0.00	0.00	0.00	0.02	0.02	0.01	0.01	-	0.00	-	0.01	0.00	0.01	-
**2A-1,36, 4-6**	2.39	0.27	0.19	0.03	0.31	0.03	-	0.38	0.11	0.01	0.02	0.01	0.02	0.04	0.01	-	-	-	-	0.00	-	-	-
**2A-1,36, 6-8**	0.09	0.02	0.01	0.00	0.02	0.01	-	0.07	0.01	0.00	0.00	-	0.00	0.00	0.00	-	-	0.00	-	0.00	-	0.00	-
**2A-1,36, 8-10**	0.07	0.02	0.00	0.00	0.06	0.01	-	0.05	0.01	0.00	0.00	0.00	0.01	0.01	0.01	-	0.00	0.00	-	-	-	-	-
**2A-1,36, 10-12**	1.92	0.21	0.13	0.02	0.16	0.01	-	0.35	0.09	0.01	0.01	0.00	0.02	0.02	0.01	-	-	0.00	-	0.01	-	-	-
**2A-1,36, 12-14**	0.54	0.14	0.03	0.01	0.15	0.00	0.00	0.21	0.04	0.00	0.01	0.00	0.01	0.03	0.01	-	-	0.00	-	0.00	0.00	0.00	0.00
**2A-1,36, 14-16**	0.15	0.02	0.01	0.00	0.06	0.01	0.00	0.05	0.01	0.00	0.00	0.00	0.01	0.00	0.00	-	-	0.00	-	0.00	-	-	-
**2A-1,36, 16-18**	0.24	0.04	0.02	0.01	0.07	0.00	0.00	0.09	0.01	0.00	0.00	0.00	0.01	0.01	0.01	-	-	0.00	-	0.00	-	-	-
**2A-1,36, 18-20**	0.88	0.18	0.09	0.02	0.16	0.01	0.00	0.28	0.06	0.01	0.01	0.01	0.01	0.02	0.02	-	-	0.00	-	0.00	-	0.00	-
**2A-1,36, 20-24**	0.93	0.09	0.08	0.01	0.08	0.00	0.00	0.17	0.04	0.00	0.01	0.00	0.01	0.01	0.00	-	-	0.00	-	0.01	-	-	-
**2A-1,36, 24-28**	0.07	0.01	0.00	0.00	0.04	0.00	0.00	0.02	0.00	0.00	0.00	0.00	0.01	0.02	0.00	-	-	0.00	-	-	-	-	-

Supplemental Table S6: The Hole (core 2) downcore lipid concentrations (μg·g^{-1} sed).

Sample Name	GDGT	OH-GDGT	GDD	OH-GDD	AR	OH-AR	Bpdiol	*br*GDGT	1G-GDGT	1G-OH-GDGT	2G-GDGT	2G-OH-GDGT	1G-AR	2G-AR	PA-AR	PA-OH-AR	PG-AR	PS-AR	PG-GDGT	Chlorophyll a	OH-Chlorophyll a	Pheophytin a	OH-Pheophytin a
**2A-1,78,0-2**	3.04	0.48	0.14	0.01	0.04	-	-	0.41	0.37	0.02	0.00	0.00	0.07	0.01	-	-	-	-	-	0.00	0.00	-	0.00
**2A-1,78,2-4**	2.33	0.38	0.12	0.04	0.21	-	0.00	0.35	0.27	0.02	0.12	0.01	0.06	0.03	-	-	-	-	0.02	0.00	-	-	-
**2A-1,78,4-6**	2.50	0.42	0.10	0.03	0.40	0.01	0.00	0.31	0.17	0.01	0.07	0.01	0.05	0.03	-	-	-	-	0.04	0.00	-	-	-
**2A-1,78,6-8**	1.93	0.37	0.10	0.04	0.51	0.01	0.00	0.34	0.10	0.01	0.11	0.01	0.05	0.03	-	-	-	-	0.10	0.00	-	-	0.00
**2A-1,78,8-10**	2.53	0.52	0.17	0.04	0.43	0.01	0.00	0.53	0.10	0.01	0.08	0.01	0.03	0.01	-	-	-	-	0.11	0.00	-	-	-
**2A-1,78,10-12**	0.43	0.05	0.02	0.01	0.38	0.03	0.00	0.10	0.02	0.00	0.01	0.00	0.07	0.03	-	-	-	-	0.01	-	-	-	-
**2A-1,78,12-14**	0.82	0.20	0.09	0.02	0.37	0.02	0.00	0.29	0.06	0.01	0.01	0.00	0.05	0.02	-	-	-	-	0.01	-	-	-	-
**2A-1,78,14-16**	0.90	0.17	0.12	0.02	0.12	0.01	0.01	0.30	0.07	0.00	0.00	0.00	0.02	0.01	-	-	-	-	0.00	-	-	-	-
**2A-1,78,16-18**	1.29	0.24	0.17	0.03	0.17	0.01	0.01	0.65	0.09	0.01	0.00	0.00	0.04	0.01	-	-	-	-	0.00	0.00	-	-	-
**2A-1,78,18-20**	1.10	0.19	0.13	0.02	0.07	0.00	0.01	0.32	0.07	0.01	0.00	0.00	0.01	0.00	-	-	-	-	0.00	0.00	-	-	0.00

Supplemental Table S7: Holey Ground (core 3) downcore lipid concentrations ($\mu g \cdot g^{-1}$ sed).

Sample Name	GDGT	OH-GDGT	GDD	OH-GDD	AR	OH-AR	Bpdiol	*br*GDGT	1G-GDGT	1G-OH-GDGT	2G-GDGT	2G-OH-GDGT	1G-AR	2G-AR	PA-AR	PA-OH-AR	PG-AR	PS-AR	PG-GDGT	Chlorophyll a	OH-Chlorophyll a	Pheophytin a	OH-Pheophytin a
**2A-1,18, 0-2**	0.16	0.03	0.01	0.00	0.01	0.00	0.00	0.04	0.03	0.00	0.00	0.00	0.01	0.01	0.01	-	0.00	0.00	-	0.01	0.01	0.00	0.00
**2A-1,18, 2-4**	3.54	0.22	0.16	0.02	0.09	0.01	0.00	0.22	0.26	0.01	0.07	0.00	0.05	0.03	0.00	0.01	0.01	0.00	0.10	-	-	-	-
**2A-1,18, 4-6**	0.71	0.09	0.02	0.01	0.26	0.02	0.00	0.08	0.03	0.00	0.12	0.00	0.05	0.04	0.01	0.01	0.01	-	-	0.00	0.00	-	0.00
**2A-1,18, 6-8**	0.18	0.02	0.00	0.00	0.11	0.01	0.00	0.03	0.02	0.00	0.03	0.00	0.04	0.04	0.01	-	0.00	0.00	-	0.01	0.00	0.00	0.00
**2A-1,18, 8-10**	0.25	0.04	0.01	0.00	0.05	0.02	-	0.05	0.02	0.00	0.02	0.00	0.02	0.02	0.00	-	0.00	0.00	-	0.00	0.00	-	0.00
**2A-1,18, 10-12**	1.07	0.14	0.07	0.02	0.24	0.02	0.00	0.14	0.13	0.00	0.14	0.01	0.06	0.04	0.01	-	0.00	0.00	0.04	0.01	0.00	0.00	0.00
**2A-1,18, 12-14**	0.40	0.08	0.02	0.01	0.15	0.01	0.00	0.07	0.02	0.00	0.03	0.00	0.04	0.02	0.01	-	0.00	0.00	0.00	0.01	-	0.00	0.00
**2A-1,18, 14-16**	0.81	0.15	0.05	0.02	0.23	0.02	0.00	0.14	0.05	0.00	0.08	0.00	0.05	0.04	0.01	0.01	0.00	0.00	0.03	0.01	0.00	0.00	0.00
**2A-1,18, 16-18**	0.22	0.05	0.02	0.00	0.04	0.01	-	0.07	0.01	0.00	0.01	0.00	0.01	0.01	0.01	-	0.00	0.00	-	0.01	0.00	-	0.00
**2A-1,18, 18-20**	0.13	0.02	0.01	0.00	0.10	0.02	0.00	0.04	0.01	0.00	0.01	0.00	0.03	0.02	0.01	-	0.01	0.00	-	0.00	0.00	0.00	0.00
**2A-1,18, 20-24**	0.38	0.04	0.02	0.01	0.09	0.01	0.00	0.06	0.02	0.00	0.01	0.00	0.02	0.01	0.01	-	0.00	0.00	-	0.00	0.00	0.00	0.00

Supplemental Table S8: Crusty White Clams (core 4) downcore lipid concentration (μg·g^{-1} sed).

Sample Name	GDGT	OH-GDGT	GDD	OH-GDD	AR	OH-AR	Bpdiol	*br*GDGT	1G-GDGT	1G-OH-GDGT	2G-GDGT	2G-OH-GDGT	1G-AR	2G-AR	PA-AR	PA-OH-AR	PG-AR	PS-AR	PG-GDGT	Chlorophyll a	OH-Chlorophyll a	Pheophytin a	OH-Pheophytin a
**2A-1,29, 0-2**	0.22	0.05	0.01	0.00	0.05	0.01	-	0.06	0.01	0.00	0.00	0.00	0.03	0.03	0.02	0.02	-	0.00	-	0.02	0.00	0.00	0.00
**2A-1,29, 2-4**	0.70	0.13	0.04	0.00	0.11	0.01	-	0.13	0.07	0.00	0.00	0.00	0.04	0.03	0.02	0.01	-	0.00	-	-	-	-	-
**2A-1,29, 4-6**	3.16	0.35	0.15	0.01	0.09	0.01	0.00	0.38	0.11	0.00	0.00	0.00	0.04	0.05	0.03	0.02	-	0.01	-	-	-	-	-
**2A-1,29, 6-8**	2.36	0.30	0.10	0.01	0.16	0.03	-	0.33	0.08	0.00	0.01	0.00	0.07	0.09	0.02	-	-	0.01	-	-	-	-	-
**2A-1,29, 8-10**	3.12	0.35	0.15	0.01	0.21	0.03	-	0.37	0.13	0.00	0.01	0.00	0.10	0.11	0.02	0.05	-	0.01	-	-	-	-	-
**2A-1,29, 10-12**	0.23	0.07	0.01	0.00	0.06	0.02	0.00	0.07	0.01	0.00	0.00	0.00	0.02	0.03	0.01	-	-	0.00	-	0.00	-	-	-
**2A-1,29, 12-14**	0.12	0.04	0.01	0.00	0.05	0.01	0.00	0.04	0.01	0.00	0.00	0.00	0.02	0.03	0.01	-	-	0.00	-	0.00	0.00	-	0.00
**2A-1,29, 14-16**	0.26	0.07	0.02	0.01	0.03	0.02	-	0.09	0.01	0.00	0.00	0.00	0.03	0.03	0.01	-	-	0.00	-	0.01	-	-	-
**2A-1,29, 16-18**	2.27	0.39	0.16	0.03	0.19	0.00	0.01	0.11	0.05	0.01	0.04	0.01	0.02	0.03	0.02	0.00	-	0.00	0.01	0.05	0.00	0.00	0.00
**2A-1,29, 18-20**	0.19	0.04	0.01	0.00	0.03	0.00	0.00	0.05	0.01	0.00	0.00	0.00	0.00	0.01	0.01	-	-	0.00	-	0.00	0.00	-	-
**2A-1,29, 20-24**	1.17	0.27	0.08	0.02	0.18	0.01	0.00	0.12	0.05	0.01	0.04	0.01	0.02	0.02	0.01	0.00	-	0.00	-	0.02	0.00	0.00	0.00
**2A-1,29, 24-28**	3.07	0.44	0.24	0.04	0.14	-	0.01	0.18	0.09	0.01	0.07	0.01	0.02	0.02	0.01	-	-	0.00	0.02	0.05	-	-	-
**2A-1,29, 28-32**	0.32	0.03	0.01	0.00	0.02	0.00	0.00	0.02	0.00	0.00	0.00	0.00	0.00	0.00	0.00	-	-	0.00	-	0.00	0.00	0.00	0.00
**2A-1,29, 32-38**	1.27	0.20	0.05	0.01	0.10	0.00	0.00	0.10	0.02	0.00	0.04	0.00	0.01	0.00	0.01	-	-	0.00	-	0.01	0.00	-	0.00

Supplemental Table S9: NW 875m Transect (core 5) downcore lipid concentration (µg·g^{-1} sed).

Sample Name	GDGT	OH-GDGT	GDD	OH-GDD	AR	OH-AR	Bpdiol	*br*GDGT	1G-GDGT	1G-OH-GDGT	2G-GDGT	2G-OH-GDGT	1G-AR	2G-AR	PA-AR	PA-OH-AR	PG-AR	PS-AR	PG-GDGT	Chlorophyll a	OH-Chlorophyll a	Pheophytin a	OH-Pheophytin a
**2A,66, 0-2**	0.89	0.07	0.06	0.00	0.00	-	0.00	0.05	0.04	0.00	0.00	0.00	0.00	-	0.01	-	-	0.00	-	0.00	0.00	-	-
**2A,66, 2-4**	0.05	0.01	0.00	0.00	0.00	-	0.00	0.01	0.00	0.00	0.00	0.00	0.00	-	0.00	-	-	0.00	-	0.00	0.00	0.00	0.00
**2A,66, 4-6**	0.02	0.01	0.00	0.00	0.00	-	0.00	0.01	0.00	0.00	0.00	0.00	0.00	-	0.00	-	-	0.00	-	-	0.00	-	0.00
**2A,66, 6-8**	0.71	0.06	0.05	0.00	0.01	-	0.00	0.06	0.04	0.00	0.00	0.00	0.00	-	0.00	-	-	0.00	-	0.04	0.00	0.00	0.00
**2A,66, 8-10**	0.43	0.04	0.02	0.00	0.00	-	0.00	0.04	0.02	0.00	0.00	0.00	0.00	-	0.00	-	-	0.00	-	0.04	0.00	0.00	0.00
**2A,66, 10-12**	0.84	0.09	0.05	0.01	0.01	-	0.00	0.08	0.07	0.00	0.00	0.00	0.00	0.00	0.01	-	-	0.00	-	0.08	0.00	0.00	0.00
**2A,66, 12-14**	0.37	0.03	0.01	0.00	0.00	-	0.00	0.03	0.02	0.00	0.00	0.00	0.00	0.00	0.00	-	-	0.00	-	0.02	0.00	0.00	0.00
**2A,66, 14-16**	2.18	0.23	0.12	0.01	0.01	-	0.00	0.14	0.13	0.01	0.01	0.01	0.00	0.00	0.01	-	-	0.00	-	0.00	0.00	0.00	0.00
**2A,66, 16-18**	0.36	0.07	0.02	0.01	0.01	-	0.00	0.04	0.02	0.00	0.00	0.00	0.00	0.00	0.01	-	-	0.00	-	0.02	0.00	0.00	0.00
**2A,66, 18-20**	0.22	0.07	0.01	0.01	0.01	-	0.00	0.03	0.01	0.00	0.00	0.00	0.00	0.00	0.01	-	-	0.00	-	0.01	0.00	0.00	0.00
**2A,66, 20-24**	0.73	0.19	0.06	0.02	0.02	-	0.00	0.09	0.04	0.00	0.00	0.01	0.00	0.01	0.02	-	-	0.00	-	0.01	0.01	0.00	0.01
**2A,66, 24-28**	2.40	0.38	0.12	0.02	0.01	-	0.01	0.14	0.13	0.01	0.01	0.01	0.00	0.00	0.02	-	-	0.00	-	0.01	0.00	0.00	0.00
**2A,66, 28-32**	0.38	0.07	0.02	0.00	0.01	-	0.00	0.04	0.01	0.00	0.00	0.00	0.00	-	0.01	-	-	0.00	-	0.02	0.00	0.00	0.00
**2A,66, 32-36**	4.07	0.67	0.34	0.06	0.02	-	0.01	0.25	0.18	0.02	0.02	0.03	0.00	0.00	0.03	-	-	0.00	-	0.11	0.01	0.00	0.02
**2A,66, 36-40**	0.52	0.09	0.02	0.00	0.01	-	0.00	0.05	0.01	0.00	0.00	0.00	0.00	-	0.01	-	-	0.00	-	0.01	0.00	0.00	0.00

Supplemental Table S10: Midpoint Transect (core 6) downcore lipid concentrations (μg·g^{-1} sed).

Sample Name	GDGT	OH-GDGT	GDD	OH-GDD	AR	OH-AR	Bpdiol	br-GDGT	1G-GDGT	1G-OH-GDGT	2G-GDGT	2G-OH-GDGT	1G-AR	2G-AR	PA-AR	PA-OH-AR	PG-AR	PS-AR	PG-GDGT	Chlorophyll a	OH-Chlorophyll a	Pheophytin a	OH-Pheophytin a
**2A,41, 0-2**	0.55	0.05	0.04	0.00	0.00	-	0.01	0.03	0.03	0.00	0.00	0.00	0.00	-	0.01	-	-	0.00	-	0.03	0.00	0.00	0.00
**2A,41, 2-4**	0.46	0.03	0.04	0.00	0.00	-	0.00	0.04	0.03	0.00	0.00	0.00	0.00	-	0.00	-	-	0.00	-	0.01	0.00	0.00	0.00
**2A,41, 4-6**	0.59	0.05	0.03	0.00	0.00	-	0.00	0.04	0.04	0.00	0.00	0.00	0.00	-	0.00	-	-	0.00	-	0.01	0.00	0.00	0.00
**2A,41, 6-8**	0.52	0.04	0.04	0.00	0.00	-	0.00	0.04	0.03	0.00	0.00	0.00	0.00	-	0.00	-	-	0.00	-	0.00	0.00	0.00	0.00
**2A,41, 8-10**	0.04	0.01	0.00	0.00	0.00	-	0.00	0.00	0.00	0.00	-	0.00	0.00	-	0.00	-	-	0.00	-	-	0.00	-	0.00
**2A,41, 10-12**	0.40	0.04	0.03	0.00	0.00	-	0.00	0.04	0.03	0.00	0.00	0.00	0.00	-	0.00	-	-	0.00	-	-	0.00	-	0.00
**2A,41, 12-14**	1.00	0.08	0.06	0.01	0.00	-	0.00	0.07	0.07	0.00	0.00	0.00	0.00	-	0.00	-	-	0.00	-	0.03	0.00	0.00	0.00
**2A,41, 14-16**	1.00	0.09	0.06	0.01	0.01	-	0.01	0.08	0.09	0.00	0.00	0.00	0.00	-	0.00	-	-	0.00	-	-	0.00	-	0.00
**2A,41, 16-18**	1.15	0.10	0.11	0.01	0.01	-	0.00	0.12	0.06	0.00	0.00	0.00	0.00	-	0.01	-	-	0.00	-	0.04	0.00	0.00	0.00
**2A,41, 18-20**	0.64	0.05	0.03	0.00	0.00	-	0.00	0.04	0.02	0.00	0.00	0.00	0.00	-	0.00	-	-	0.00	-	-	0.00	-	0.00
**2A,41, 20-24**	0.47	0.07	0.02	0.01	0.00	-	0.00	0.05	0.03	0.00	0.00	0.00	0.00	-	0.01	-	-	0.00	-	-	0.00	-	0.00
**2A,41, 24-28**	2.04	0.27	0.13	0.02	0.01	-	0.00	0.16	0.10	0.01	0.01	0.01	0.00	-	0.00	-	-	0.00	-	0.00	0.01	-	0.01
**2A,41, 28-32**	0.15	0.04	0.01	0.00	0.00	-	-	0.03	0.01	0.00	0.00	0.00	0.00	-	0.00	-	-	0.00	-	0.01	0.00	0.00	0.00
**2A,41, 32-36**	2.83	0.46	0.23	0.04	0.01	-	0.00	0.22	0.13	0.01	0.01	0.01	0.00	-	0.01	-	-	0.00	-	0.05	0.01	0.00	0.00

Supplemental Table S11: Deep Purple (core 1) downcore lipid concentration (µg·g^{-1} sed).

Sample Name	GDGT-0	GDGT-1	GDGT-2	GDGT-3	GDGT-4	GDGT-5	GDGT-5´	1G-GDGT-0	1G-GDGT-1	1G-GDGT-2	1G-GDGT-3	1G-GDGT-4	1G-GDGT-5	1G-GDGT-5´	2G-GDGT-0	2G-GDGT-1	2G-GDGT-2	2G-GDGT-3	2G-GDGT-4	1G-GDGT-5	1G-GDGT-5´
**2A-1,36, 0-2**	0.59	0.25	0.28	0.03	0.24	0.50	0.02	0.02	0.00	0.01	0.00	0.01	0.02	0.00	0.00	0.00	0.00	0.00	0.00	0.00	0.00
**2A-1,36, 2-4**	0.21	0.06	0.07	0.01	0.06	0.13	0.00	0.01	0.00	0.00	0.00	0.00	0.01	0.00	0.00	0.00	0.00	0.00	0.00	0.00	0.00
**2A-1,36, 4-6**	0.73	0.31	0.37	0.05	0.31	0.61	0.01	0.04	0.01	0.01	0.00	0.02	0.03	0.00	0.00	0.01	0.01	0.00	0.00	0.00	0.00
**2A-1,36, 6-8**	0.03	0.01	0.01	0.00	0.01	0.02	0.00	0.00	0.00	0.00	0.00	0.00	0.00	0.00	0.00	0.00	0.00	0.00	0.00	0.00	0.00
**2A-1,36, 8-10**	0.03	0.01	0.01	0.00	0.01	0.02	0.00	0.00	0.00	0.00	0.00	0.00	0.00	0.00	0.00	0.00	0.00	0.00	0.00	0.00	0.00
**2A-1,36, 10-12**	0.60	0.25	0.30	0.04	0.23	0.49	0.01	0.04	0.01	0.01	0.00	0.01	0.03	0.00	0.00	0.01	0.01	0.00	0.00	0.00	0.00
**2A-1,36, 12-14**	0.22	0.07	0.07	0.01	0.05	0.12	0.00	0.01	0.00	0.00	0.00	0.01	0.01	0.00	0.00	0.00	0.00	0.00	0.00	0.00	0.00
**2A-1,36, 14-16**	0.05	0.02	0.02	0.00	0.02	0.04	0.00	0.00	0.00	0.00	0.00	0.00	0.00	0.00	0.00	0.00	0.00	0.00	0.00	0.00	0.00
**2A-1,36, 16-18**	0.08	0.02	0.03	0.00	0.03	0.06	0.00	0.00	0.00	0.00	0.00	0.00	0.00	0.00	0.00	0.00	0.00	0.00	0.00	0.00	0.00
**2A-1,36, 18-20**	0.32	0.10	0.13	0.01	0.11	0.20	0.01	0.02	0.01	0.00	0.00	0.01	0.02	0.00	0.00	0.01	0.01	0.00	0.00	0.00	0.00
**2A-1,36, 20-24**	0.28	0.12	0.15	0.02	0.11	0.24	0.01	0.02	0.00	0.00	0.00	0.01	0.01	0.00	0.00	0.00	0.00	0.00	0.00	0.00	0.00
**2A-1,36, 24-28**																					

Supplemental Table S12: The Hole (core 2) downcore lipid concentrations ($\mu g \cdot g^{-1}$ sed).

Sgample Name	GDGT-0	GDGT-1	GDGT-2	GDGT-3	GDGT-4	GDGT-5	GDGT-5´	1G-GDGT-0	1G-GDGT-1	1G-GDGT-2	1G-GDGT-3	1G-GDGT-4	1G-GDGT-5	1G-GDGT-5´	2G-GDGT-0	2G-GDGT-1	2G-GDGT-2	2G-GDGT-3	2G-GDGT-4	1G-GDGT-5	1G-GDGT-5´
**2A-1,78,0-2**	0.97	0.64	0.86	0.15	0.32	0.07	0.02	0.10	0.05	0.11	0.02	0.03	0.06	0.00	0.00	0.00	0.00	0.00	0.00	0.00	0.97
**2A-1,78,2-4**	0.63	0.54	0.66	0.10	0.13	0.26	0.01	0.09	0.04	0.08	0.01	0.02	0.04	0.00	0.02	0.04	0.05	0.01	0.00	0.00	0.63
**2A-1,78,4-6**	0.61	0.63	0.78	0.17	0.10	0.19	0.01	0.06	0.02	0.04	0.01	0.02	0.03	0.00	0.01	0.03	0.03	0.01	0.00	0.00	0.61
**2A-1,78,6-8**	0.48	0.43	0.58	0.11	0.10	0.21	0.01	0.04	0.01	0.01	0.00	0.01	0.02	0.00	0.01	0.04	0.05	0.01	0.00	0.00	0.48
**2A-1,78,8-10**	0.66	0.56	0.69	0.14	0.14	0.32	0.01	0.03	0.01	0.01	0.00	0.02	0.03	0.00	0.01	0.03	0.03	0.01	0.00	0.00	0.66
**2A-1,78,10-12**	0.11	0.07	0.11	0.02	0.04	0.08	0.00	0.01	0.00	0.00	0.00	0.00	0.01	0.00	0.00	0.00	0.00	0.00	0.00	0.00	0.11
**2A-1,78,12-14**	0.31	0.10	0.11	0.02	0.09	0.19	0.00	0.02	0.00	0.00	0.00	0.01	0.02	0.00	0.00	0.00	0.01	0.00	0.00	0.00	0.31
**2A-1,78,14-16**	0.35	0.06	0.05	0.02	0.13	0.28	0.01	0.02	0.00	0.00	0.00	0.01	0.03	0.00	0.00	0.00	0.00	0.00	0.00	0.00	0.35
**2A-1,78,16-18**	0.48	0.10	0.11	0.03	0.18	0.38	0.01	0.03	0.00	0.00	0.00	0.02	0.03	0.00	0.00	0.00	0.00	0.00	0.00	0.00	0.48
**2A-1,78,18-20**																					

Supplemental Table S13: Holey Ground (core 3) downcore lipid concentrations ($\mu g \cdot g^{-1}$ sed).

Sample Name	GDGT-0	GDGT-1	GDGT-2	GDGT-3	GDGT-4	GDGT-5	GDGT-5´	1G-GDGT-0	1G-GDGT-1	1G-GDGT-2	1G-GDGT-3	1G-GDGT-4	1G-GDGT-5	1G-GDGT-5´	2G-GDGT-0	2G-GDGT-1	2G-GDGT-2	2G-GDGT-3	2G-GDGT-4	1G-GDGT-5	1G-GDGT-5´
**2A-1,18, 0-2**	0.05	0.03	0.04	0.01	0.01	0.02	0.00	0.00	0.01	0.01	0.00	0.00	0.00	0.00	0.00	0.00	0.00	0.00	0.00	0.00	0.00
**2A-1,18, 2-4**	0.81	0.70	0.93	0.31	0.25	0.52	0.02	0.05	0.06	0.12	0.02	0.00	0.01	0.00	0.01	0.02	0.03	0.01	0.00	0.00	0.00
**2A-1,18, 4-6**	0.15	0.17	0.27	0.07	0.02	0.03	0.00	0.01	0.01	0.01	0.00	0.00	0.00	0.00	0.01	0.04	0.05	0.01	0.00	0.00	0.00
**2A-1,18, 6-8**	0.04	0.04	0.07	0.01	0.01	0.01	0.00	0.00	0.01	0.01	0.00	0.00	0.00	0.00	0.00	0.01	0.01	0.00	0.00	0.00	0.00
**2A-1,18, 8-10**	0.06	0.05	0.07	0.03	0.02	0.03	0.00	0.00	0.01	0.01	0.00	0.00	0.00	0.00	0.01	0.01	0.01	0.00	0.00	0.00	0.00
**2A-1,18, 10-12**	0.25	0.23	0.36	0.09	0.05	0.09	0.00	0.04	0.03	0.05	0.01	0.01	0.02	0.00	0.02	0.05	0.06	0.01	0.00	0.00	0.00
**2A-1,18, 12-14**	0.10	0.08	0.12	0.03	0.03	0.05	0.00	0.01	0.00	0.00	0.00	0.00	0.00	0.00	0.00	0.01	0.01	0.00	0.00	0.00	0.00
**2A-1,18, 14-16**	0.20	0.16	0.26	0.06	0.04	0.08	0.00	0.02	0.01	0.01	0.00	0.01	0.01	0.00	0.01	0.03	0.03	0.01	0.00	0.00	0.00
**2A-1,18, 16-18**	0.05	0.03	0.05	0.02	0.02	0.04	0.00	0.00	0.00	0.00	0.00	0.00	0.00	0.00	0.00	0.00	0.00	0.00	0.00	0.00	0.00
**2A-1,18, 18-20**	0.03	0.02	0.04	0.01	0.01	0.02	0.00	0.00	0.00	0.00	0.00	0.00	0.00	0.00	0.00	0.00	0.00	0.00	0.00	0.00	0.00
**2A-1,18, 20-24**	0.09	0.06	0.10	0.03	0.03	0.07	0.00	0.01	0.00	0.00	0.00	0.00	0.00	0.00	0.00	0.00	0.00	0.00	0.00	0.00	0.00

Supplemental Table S14: Crusty White Clams (core 4) downcore lipid concentration ($\mu g \cdot g^{-1}$ sed).

Sample Name	GDGT-0	GDGT-1	GDGT-2	GDGT-3	GDGT-4	GDGT-5	GDGT-5´	1G-GDGT-0	1G-GDGT-1	1G-GDGT-2	1G-GDGT-3	1G-GDGT-4	1G-GDGT-5	1G-GDGT-5´	2G-GDGT-0	2G-GDGT-1	2G-GDGT-2	2G-GDGT-3	2G-GDGT-4	1G-GDGT-5	1G-GDGT-5´
**2A-1,29, 0-2**	0.10	0.02	0.02	0.00	0.03	0.05	0.00	0.01	0.00	0.00	0.00	0.00	0.00	0.00	0.00	0.00	0.00	0.00	0.00	0.00	0.00
**2A-1,29, 2-4**	0.31	0.07	0.06	0.01	0.09	0.18	0.00	0.03	0.01	0.02	0.00	0.00	0.01	0.00	0.00	0.00	0.00	0.00	0.00	0.00	0.00
**2A-1,29, 4-6**	1.00	0.36	0.35	0.05	0.46	0.94	0.02	0.03	0.02	0.04	0.00	0.01	0.01	0.00	0.00	0.00	0.00	0.00	0.00	0.00	0.00
**2A-1,29, 6-8**	0.75	0.28	0.29	0.04	0.32	0.66	0.02	0.02	0.01	0.03	0.00	0.01	0.01	0.00	0.00	0.00	0.00	0.00	0.00	0.00	0.00
**2A-1,29, 8-10**	1.04	0.36	0.38	0.05	0.41	0.86	0.02	0.03	0.02	0.05	0.01	0.01	0.01	0.00	0.00	0.00	0.00	0.00	0.00	0.00	0.00
**2A-1,29, 10-12**	0.10	0.02	0.02	0.00	0.03	0.06	0.00	0.00	0.00	0.01	0.00	0.00	0.00	0.00	0.00	0.00	0.00	0.00	0.00	0.00	0.00
**2A-1,29, 12-14**	0.06	0.01	0.01	0.00	0.01	0.03	0.00	0.00	0.00	0.00	0.00	0.00	0.00	0.00	0.00	0.00	0.00	0.00	0.00	0.00	0.00
**2A-1,29, 14-16**	0.08	0.03	0.04	0.01	0.04	0.07	0.00	0.00	0.00	0.00	0.00	0.00	0.00	0.00	0.00	0.00	0.00	0.00	0.00	0.00	0.00
**2A-1,29, 16-18**	0.74	0.32	0.38	0.04	0.26	0.52	0.01	0.01	0.00	0.01	0.00	0.01	0.01	0.00	0.01	0.02	0.01	0.00	0.00	0.00	0.00
**2A-1,29, 18-20**	0.05	0.03	0.03	0.01	0.03	0.05	0.00	0.00	0.00	0.00	0.00	0.00	0.00	0.00	0.00	0.00	0.00	0.00	0.00	0.00	0.00
**2A-1,29, 20-24**	0.43	0.16	0.20	0.02	0.12	0.24	0.00	0.01	0.00	0.01	0.00	0.01	0.01	0.00	0.01	0.01	0.01	0.00	0.00	0.00	0.00
**2A-1,29, 24-28**	0.86	0.55	0.75	0.11	0.04	0.75	0.02	0.02	0.01	0.01	0.00	0.02	0.03	0.00	0.01	0.03	0.03	0.00	0.00	0.00	0.00
**2A-1,29, 28-32**	0.10	0.04	0.05	0.00	0.04	0.09	0.00	0.00	0.00	0.00	0.00	0.00	0.00	0.00	0.00	0.00	0.00	0.00	0.00	0.00	0.00
**2A-1,29, 32-38**																					

Supplemental Table S15: NW 875m Transect (core 5) downcore lipid concentration ($\mu g \cdot g^{-1}$ sed).

Sample Name	GDGT-0	GDGT-1	GDGT-2	GDGT-3	GDGT-4	GDGT-5	GDGT-5´	1G-GDGT-0	1G-GDGT-1	1G-GDGT-2	1G-GDGT-3	1G-GDGT-4	1G-GDGT-5	1G-GDGT-5´	2G-GDGT-0	2G-GDGT-1	2G-GDGT-2	2G-GDGT-3	2G-GDGT-4	1G-GDGT-5	1G-GDGT-5´
**2A,66, 0-2**	0.30	0.07	0.05	0.01	0.15	0.30	0.01	0.02	0.00	0.00	0.00	0.01	0.01	0.00	0.00	0.00	0.00	0.00	0.00	0.00	0.00
**2A,66, 2-4**	0.02	0.00	0.00	0.00	0.01	0.01	0.00	0.00	0.00	0.00	0.00	0.00	0.00	0.00	0.00	0.00	0.00	0.00	0.00	0.00	0.00
**2A,66, 4-6**	0.01	0.00	0.00	0.00	0.00	0.01	0.00	0.00	0.00	0.00	0.00	0.00	0.00	0.00	0.00	0.00	0.00	0.00	0.00	0.00	0.00
**2A,66, 6-8**	0.25	0.05	0.03	0.00	0.12	0.25	0.01	0.02	0.00	0.00	0.00	0.01	0.01	0.00	0.00	0.00	0.00	0.00	0.00	0.00	0.00
**2A,66, 8-10**	0.17	0.03	0.01	0.00	0.07	0.14	0.00	0.01	0.00	0.00	0.00	0.00	0.00	0.00	0.00	0.00	0.00	0.00	0.00	0.00	0.00
**2A,66, 10-12**	0.32	0.06	0.04	0.00	0.14	0.27	0.01	0.03	0.00	0.00	0.00	0.01	0.02	0.00	0.00	0.00	0.00	0.00	0.00	0.00	0.00
**2A,66, 12-14**	0.14	0.02	0.01	0.00	0.06	0.13	0.00	0.01	0.00	0.00	0.00	0.00	0.01	0.00	0.00	0.00	0.00	0.00	0.00	0.00	0.00
**2A,66, 14-16**	0.68	0.21	0.18	0.03	0.34	0.69	0.04	0.04	0.00	0.00	0.00	0.03	0.05	0.00	0.00	0.00	0.00	0.00	0.00	0.00	0.00
**2A,66, 16-18**	0.14	0.02	0.01	0.00	0.06	0.13	0.00	0.01	0.00	0.00	0.00	0.00	0.00	0.00	0.00	0.00	0.00	0.00	0.00	0.00	0.00
**2A,66, 18-20**	0.10	0.01	0.01	0.00	0.03	0.06	0.00	0.01	0.00	0.00	0.00	0.00	0.00	0.00	0.00	0.00	0.00	0.00	0.00	0.00	0.00
**2A,66, 20-24**	0.34	0.05	0.02	0.00	0.10	0.21	0.00	0.02	0.00	0.00	0.00	0.01	0.01	0.00	0.00	0.00	0.00	0.00	0.00	0.00	0.00
**2A,66, 24-28**	1.03	0.15	0.08	0.01	0.37	0.76	0.02	0.05	0.00	0.00	0.00	0.03	0.05	0.00	0.00	0.00	0.00	0.00	0.00	0.00	0.00
**2A,66, 28-32**	0.15	0.02	0.01	0.00	0.07	0.13	0.00	0.01	0.00	0.00	0.00	0.00	0.00	0.00	0.00	0.00	0.00	0.00	0.00	0.00	0.00
**2A,66, 32-36**	1.79	0.33	0.21	0.03	0.77	0.90	0.04	0.04	0.01	0.00	0.00	0.05	0.08	0.00	0.01	0.00	0.00	0.00	0.00	0.00	0.00
**2A,66, 36-40**	0.19	0.03	0.02	0.00	0.09	0.18	0.00	0.00	0.00	0.00	0.00	0.00	0.01	0.00	0.00	0.00	0.00	0.00	0.00	0.00	0.00

Supplemental Table S16: Midpoint Transect (core 6) downcore lipid concentrations (μg·g^{-1} sed).

Sample Name	GDGT-0	GDGT-1	GDGT-2	GDGT-3	GDGT-4	GDGT-5	GDGT-5´	1G-GDGT-0	1G-GDGT-1	1G-GDGT-2	1G-GDGT-3	1G-GDGT-4	1G-GDGT-5	1G-GDGT-5´	2G-GDGT-0	2G-GDGT-1	2G-GDGT-2	2G-GDGT-3	2G-GDGT-4	1G-GDGT-5	1G-GDGT-5´
**2A,41, 0-2**	0.21	0.04	0.03	0.00	0.08	0.18	0.00	0.02	0.00	0.00	0.00	0.01	0.01	0.00	0.00	0.00	0.00	0.00	0.00	0.00	0.00
**2A,41, 2-4**	0.19	0.04	0.02	0.00	0.01	0.19	0.00	0.02	0.00	0.00	0.00	0.01	0.01	0.00	0.00	0.00	0.00	0.00	0.00	0.00	0.00
**2A,41, 4-6**	0.21	0.04	0.03	0.00	0.10	0.20	0.00	0.02	0.00	0.00	0.00	0.01	0.01	0.00	0.00	0.00	0.00	0.00	0.00	0.00	0.00
**2A,41, 6-8**	0.17	0.04	0.03	0.00	0.09	0.18	0.01	0.02	0.00	0.00	0.00	0.01	0.01	0.00	0.00	0.00	0.00	0.00	0.00	0.00	0.00
**2A,41, 8-10**	0.01	0.00	0.00	0.00	0.01	0.01	0.00	0.00	0.00	0.00	0.00	0.00	0.00	0.00	0.00	0.00	0.00	0.00	0.00	0.00	0.00
**2A,41, 10-12**	0.15	0.03	0.02	0.00	0.07	0.13	0.00	0.01	0.00	0.00	0.00	0.01	0.01	0.00	0.00	0.00	0.00	0.00	0.00	0.00	0.00
**2A,41, 12-14**	0.33	0.07	0.05	0.01	0.17	0.36	0.01	0.03	0.00	0.00	0.00	0.01	0.02	0.00	0.00	0.00	0.00	0.00	0.00	0.00	0.00
**2A,41, 14-16**	0.38	0.06	0.04	0.01	0.17	0.35	0.01	0.04	0.00	0.00	0.00	0.02	0.03	0.00	0.00	0.00	0.00	0.00	0.00	0.00	0.00
**2A,41, 16-18**	0.39	0.09	0.06	0.01	0.19	0.40	0.01	0.03	0.00	0.00	0.00	0.01	0.02	0.00	0.00	0.00	0.00	0.00	0.00	0.00	0.00
**2A,41, 18-20**	0.21	0.04	0.03	0.00	0.12	0.23	0.01	0.01	0.00	0.00	0.00	0.00	0.01	0.00	0.00	0.00	0.00	0.00	0.00	0.00	0.00
**2A,41, 20-24**	0.17	0.03	0.02	0.00	0.08	0.16	0.00	0.01	0.00	0.00	0.00	0.01	0.01	0.00	0.00	0.00	0.00	0.00	0.00	0.00	0.00
**2A,41, 24-28**	0.66	0.20	0.15	0.00	0.32	0.67	0.03	0.02	0.00	0.00	0.00	0.03	0.05	0.00	0.00	0.00	0.00	0.00	0.00	0.00	0.00
**2A,41, 28-32**	0.05	0.01	0.01	0.00	0.03	0.05	0.00	0.00	0.00	0.00	0.00	0.00	0.00	0.00	0.00	0.00	0.00	0.00	0.00	0.00	0.00

Supplemental Table S17: Average Simpson's Diversity Index for IPL and CL community in each core.

	IPL				CL			
**Site**	**D**	**1-D**	**Max**	**Min**	**D**	**1-D**	**Max**	**Min**
Deep Purple	0.25	0.75	0.83	0.66	0.38	0.62	0.75	0.37
The Hole	0.37	0.63	0.79	0.35	0.40	0.60	0.71	0.43
Holey Ground	0.23	0.77	0.82	0.71	0.41	0.59	0.72	0.30
Crusty White Clams	0.24	0.76	0.83	0.68	0.47	0.53	0.72	0.36
NW 875m Transect	0.41	0.59	0.74	0.40	0.57	0.44	0.64	0.32
Midpoint	0.50	0.50	0.74	0.35	0.62	0.38	0.56	0.28

Supplemental Table S18: Summary of lipid proxies calculated at Site 2A-1. Data is organized downcore by location across the transect.

Sample Name	MI_{CL}	MI_{IPL}	*br*SRI	AR:OH-AR
2A-1,36, 0-2	0.52	0.51	0.72	0.03
2A-1,36, 2-4	0.50	0.40	0.72	0.09
2A-1,36, 4-6	0.54	0.34	0.73	0.09
2A-1,36, 6-8	0.52	0.34	0.73	0.54
2A-1,36, 8-10	0.49	0.38	0.69	0.14
2A-1,36, 10-12	0.54	0.35	0.71	0.04
2A-1,36, 12-14	0.55	0.33	0.73	0.03
2A-1,36, 14-16	0.45	0.44	0.72	0.12
2A-1,36, 16-18	0.47	0.39	0.76	0.07
2A-1,36, 18-20	0.54	0.37	0.74	0.03
2A-1,36, 20-24	0.54	0.38	0.74	0.02
2A-1,36, 24-28	0.51	0.19	0.64	0.08
2A-1,78,0-2	0.95	0.76	0.54	0.00
2A-1,78,2-4	0.83	0.77	0.71	0.00
2A-1,78,4-6	0.89	0.68	0.72	0.01
2A-1,78,6-8	0.84	0.50	0.78	0.03
2A-1,78,8-10	0.81	0.34	0.76	0.01
2A-1,78,10-12	0.73	0.24	0.77	0.07
2A-1,78,12-14	0.54	0.19	0.77	0.05
2A-1,78,14-16	0.30	0.15	0.76	0.06
2A-1,78,16-18	0.38	0.21	0.83	0.06
2A-1,78,18-20	0.25	0.14	0.69	0.04
2A-1,18, 0-2	0.74	0.98	0.61	0.16
2A-1,18, 2-4	0.78	0.97	0.63	0.13
2A-1,18, 4-6	0.94	0.93	0.64	0.09
2A-1,18, 6-8	0.91	0.97	0.64	0.10
2A-1,18, 8-10	0.81	0.87	0.60	0.39
2A-1,18, 10-12	0.88	0.81	0.69	0.07
2A-1,18, 12-14	0.82	0.63	0.60	0.09

2A-1,18, 14-16	0.85	0.63	0.70	0.07
2A-1,18, 16-18	0.71	0.62	0.64	0.19
2A-1,18, 18-20	0.80	0.66	0.72	0.19
2A-1,18, 20-24	0.74	0.57	0.72	0.17
2A-1,29, 0-2	0.42	0.66	0.56	0.22
2A-1,29, 2-4	0.42	0.77	0.57	0.12
2A-1,29, 4-6	0.44	0.86	0.53	0.14
2A-1,29, 6-8	0.47	0.78	0.57	0.18
2A-1,29, 8-10	0.47	0.84	0.43	0.15
2A-1,29, 10-12	0.37	0.92	0.59	0.28
2A-1,29, 12-14	0.42	0.89	0.57	0.16
2A-1,29, 14-16	0.49	0.61	0.55	0.47
2A-1,29, 16-18	0.58	0.53	0.54	0.03
2A-1,29, 18-20	0.56	0.33	0.60	0.16
2A-1,29, 20-24	0.61	0.45	0.66	0.03
2A-1,29, 24-28	0.65	0.37	0.59	0.00
2A-1,29, 28-32	0.49	0.26	0.65	0.14
2A-1,29, 32-38	0.64	0.37	0.65	0.01
2A,66, 0-2	0.30	0.14	0.46	0.00
2A,66, 2-4	0.24	0.16	0.48	0.00
2A,66, 4-6	0.20	0.17	0.50	0.00
2A,66, 6-8	0.26	0.12	0.50	0.00
2A,66, 8-10	0.23	0.12	0.45	0.00
2A,66, 10-12	0.26	0.11	0.47	0.00
2A,66, 12-14	0.21	0.08	0.45	0.00
2A,66, 14-16	0.36	0.11	0.51	0.00
2A,66, 16-18	0.18	0.14	0.59	0.00
2A,66, 18-20	0.22	0.15	0.55	0.00
2A,66, 20-24	0.25	0.14	0.55	0.00
2A,66, 24-28	0.23	0.11	0.57	0.00
2A,66, 28-32	0.23	0.13	0.54	0.00
2A,66, 32-36	0.38	0.10	0.56	0.00

2A,66, 36-40	0.25	0.11	0.56	0.00
2A,41, 0-2	0.29	0.13	0.45	0.00
2A,41, 2-4	0.25	0.16	0.46	0.00
2A,41, 4-6	0.26	0.15	0.47	0.00
2A,41, 6-8	0.27	0.16	0.45	0.00
2A,41, 8-10	0.24	0.13	0.42	0.00
2A,41, 10-12	0.25	0.14	0.44	0.00
2A,41, 12-14	0.26	0.14	0.47	0.00
2A,41, 14-16	0.21	0.11	0.43	0.00
2A,41, 16-18	0.27	0.12	0.41	0.00
2A,41, 18-20	0.24	0.09	0.39	0.00
2A,41, 20-24	0.25	0.11	0.52	0.00
2A,41, 24-28	0.34	0.11	0.53	0.00
2A,41, 28-32	0.26	0.09	0.52	0.00
2A,41, 32-36	0.30	0.09	0.50	0.00
